# Supplementary material for: A Coarse-Grained Biophysical Model of E. coli and Its Application to Perturbation of the rRNA Operon Copy Number
Source: PLoS Comput Biol. 2008 May 2;4(5):e1000038. doi: 10.1371/journal.pcbi.1000038 (PMC2320978; doi:10.1371/journal.pcbi.1000038)
Supplement: Text S1 — Complete Supporting Information (0.56 MB PDF) [file pcbi.1000038.s001.pdf]

A coarse-grained biophysical model of *E. coli* and its application to perturbation of the rRNA operon copy number

Supporting information

Contents:

List of figures and tables………………………………………………………………………. 2

S1 Observational data and further Results……………………………………..………….. 3
S1.1 Cell state and genetic parameters at 1 and 2.5 doub/h……………………..…………. 3
S1.1.1 Reconstruction of the wild-type cell state at 1 and 2.5 doub/h ……………….……. 7
S1.2 Reconstruction of *E. coli* WT genetic parameters at 2 doub/h…………………….….… 9
S1.3 Genetic parameters of *rrn* inactivation strains at 2 doub/h …………………………..… 13
S1.4 Genetic parameters of strains with increased *rrn* gene dosage…………………………….. 19
S1.5 Mean square errors with respect to the Squires data…………………………………..… 20
S1.6 Free RNAp and free ribosome homeostasis…………………………...………..... 22
S1.7 Further predictions……………………………………………………...………….. 24

S2 Model - supplementary information………………………………………………………… 25
S2.1 RNAp autoregulation…………………………………………………………….……… 25
S2.2 Gene concentration……………………………………………………………………… 27
S2.2.1 Growth rate dependence of gene concentration……………………………….…… 27
S2.2.2 The initiation volume………………………………………………………….…… 28
S2.3 Genetic vs. environmental perturbations……………………………………………… 28
S2.4 Crowding………………………………………………………………………………… 30
S2.4.1 Overview of crowding effects ……………………………………………..….. 30
S2.4.2 Calculation of activity coefficients and binding affinities as a function of Φ..….. 33
S2.4.3 Crowding scenarios………………………………………………………………… 36
S2.4.3.1 The transition state limited scenario……………………...…………. 36
S2.4.3.2 The diffusion limited scenario……………………………………………. 37
S2.5 Derivations……………………………………………………………………………. 38
S2.5.1 Approximation of immature pool sizes………………………….....………… 38
S2.5.2 The steady state rate equations……………………………………………………… 40
S2.6 Summary of the CGGR model equations……………………………………..……… 49
S2.6.1 The unconstrained CGGR………………………………………………………… 49
S2.6.2 The constrained CGGR…………………………………………………….. 50
S2.7 mRNA degradation………………………………………………………………………. 51
S2.7.1 mRNA degradation via RNase E…………………………………………………. 51
S2.7.2 mRNA half-life based on the 5' competition model……………………………… 51
S2.7.3 mRNA degradation should not be affected by crowding………………………… 53
S2.7.4 Model assuming nucleolytic inactivation of mRNA……………………………... 54

S3 The simplified model ……………………………………………………………..……….. 55
S3.1 Analytical solution……………………………………………………………………….. 55
S3.2 Approximations made………………………………………………………………… 55

References……………………………………………………………………...…………. 57

List of tables and figures

Table S1 Genetic parameters for *E. coli* growing at 1 and 2.5 doub/h, 37°C 3
Table S2 Cell state and additional parameters for various growth conditions 5
Table S3 Reconstruction of the WT cell state for 1 and 2.5 doub/h, 37°C 8
Table S4 Transcription related parameters for 2 doub/h, 37°C 9
Table S5 Genetic parameters for *E. coli* growing at 2 doub/h, 37°C 12
Table S6 Lineage of the *rrn* inactivation strains 14
Table S7 Genetic parameters for the *rrn* inactivation strains at 2 doub/h, 37°C 17
Table S8 Notation used for derivations 40

Figure S1 Mean square errors with respect to the Squires data 20
Figure S2 Fit for the constrained CGGR model with Hill coefficient h=1 21
Figure S3 Fit for the constrained CGGR model with higher Hill coefficients 21
Figure S4 Free RNAp and free ribosomes with respect to corresponding binding affinities for various crowding scenarios 23
Figure S5 Predictions for bulk protein and ribosome concentrations as a function of the *rrn* operon copy number 24
Figure S6 Breakdown of the ribosome synthesis equation to components for the diffusion limited scenario 24
Figure S7 Gene dosage and gene concentration as a function of growth rate 27
Figure S8 Dependence of binding affinities on the volume fraction Φ for the various crowding scenarios 32
Figure S9 Dependance of activity coeficients on the crowder radius 33
Figure S10 Dependence of activity coefficients on the volume fraction 35
Figure S11 Relation between free ribosome and total ribosome concentration 56

S1. Observational data and further Results

S1.1 Cell state and genetic parameters at 1 and 2.5 doub/h

Table S1. Genetic parameters for *E. coli* growing at 1 and 2.5 doub/h, 37°C. Values not in parentheses are for 2.5 doub/h. Values in parentheses are for 1 doub/h, with the rest of the values being identical to those at 2.5 doub/h. Note that all concentrations have been multiplied by the average cell volume $V_{cell}(\mu_0)$ of a WT cell growing in the given medium, i.e. μ_0=2.5 doub/h (and 1 doub/h). Cell volumes are given in Table S2. Using these units for $K_{m,i}$, $L_{m,i}$ and d_i when solving the equations of state for a genetically perturbed cell generates concentrations (e.g. $n_{RNAp,free}$, $n_{ribo,free}$ etc.) also in these units, i.e. concentration times $V_{cell}(\mu_0)$. Values in bold are estimates (see respective footnotes and main text).

Gene class		Units	r-protein[a]	bulk[b]	*rrn*[c]
m [d]	Map location	MU (min)	see footnote d	66 (417) uniformly distributed genes	C 84.5; D 72.1; G 56.1; H 5.1; B 89.8; E 90.5; A 86.5
$V_i^{\max}$ [e]	Maximum transcription initiation rate	ini/min	33	3.04 (1.71)	110
$U_i^{\max}$ [f]	Maximum translation initiation rate	ini/min	-	**80**	-
$K_{m,i}$ [g]	Promoter-RNAp holoenzyme binding affinity	molec/cell	501 (264)	501 (264)	797 (1440)
$L_{m,i}$ [h]	RBS-30S ribosome subunit binding affinity	molec/cell	-	**6144 (3226)**	-
$T_{1/2,i}^{fun}$ [i]	mRNA half-life	min	-	6.8 **(1.28)**	-
L_i [j]	Gene class length	base pairs	21252	1000	6623
c_p [k]	Peptide chain elongation rate	aa/sec	21 (16)	21 (16)	-
c_i [l]	RNA chain elongation rate	nuc/sec	55 (45)	1.37 (4.09)	85

[a] This class is analogous to the constitutive gene class of [1], where we consolidated all r-protein genes into a single operon (c.f. footnote d). Note that r-protein is assumed to be constitutive as transcription of most r-protein operons is not growth rate regulated [2].
[b] Bulk gene class gene dosage D_{bulk}, maximum transcription initiation rate, V^{max}_{bulk}, and transcription elongation rate, c_{bulk}, are a consolidation of repressable (r) and pause (ps) promoter classes of [1] such that the bulk gene class is mathematically equivalent to the sum of these two promoter classes in terms of transcription and translation (see below).
[c] *rrn* gene class is a consolidation of P1 and P2 *rrn* promoter classes in [1].
[d] To convert from MU (min) (0<MU<100 min) to units relative to oriC (0<m<1) see table 5 in [3]. *rrn* map locations were taken from [4]. Bulk protein genes are assumed to be uniformly distributed across the chromosome: m = (0,1,…, N_{bulk}-1)/N_{bulk}. The number of bulk genes, N_{bulk}, was ascertained using Eq. S7 for the gene dosage: $D_{bulk}(\mu)=\sum_{i=0}^{N_{bulk}-1} 2^{\mu(C(1-m_i)+D)} = 2^{\mu(C+D)}\left(1-2^{-\mu C}\right)/\left(1-2^{-\mu C/N_{bulk}}\right)$, $m_i = i/N_{bulk}$, and solving for N_{bulk}, where $D_{bulk} = D_r + D_{ps}$ and D_r and D_{ps} are the gene dosage of repressable and pause promoter classes respectively, taken from tables 1 and 2 of [1]: D_{bulk}(2.5 doub/h) = 251 and D_{bulk}(1 doub/h) = 771. Gene concentrations $d_i(\mu)$ in Eq. 2 can be calculated using Eq. 3: $d_i(\mu)=\frac{1}{\ln 2 V_{ini}}\sum_j 2^{-m_j^{(i)}C\mu}$, where $\left(V_{ini}\ln 2\right)^{-1} = V_{cell}^{-1}(\mu_0)2^{\mu_0(C+D)}$ (see Eq. S8) is evaluated for a high enough μ_0. For example, for μ_0 = 2.5 doub/h, $\left(V_{ini}\ln 2\right)^{-1} = 5.45(\mu m)^{-3}$ (see Table S2 for WT cell volumes). In practice, for genetic perturbation simulations it is convenient to use concentration in units of concentration·$V_{cell}(\mu_0)$, where μ_0 is chosen to be the growth rate of a WT cell in the given environment. Thus $d_i(\mu)V_{cell}(\mu_0)=2^{\mu_0(C+D)}\sum_j 2^{-m_j^{(i)}C\mu}$. The factor $2^{\mu_0(C+D)}$ is 6.54 for 2.5 doub/h and 2.43 for 1 doub/h (C and D are given in Table S2). The only exception to this rule is in the calculation of gene concentration for the r-protein gene class. Since the 19 operons of r-protein were concatenated on a *single* operon, in order to capture a more realistic growth rate dependence of the gene concentration, the following formula was used: $d_{r-protein}(\mu)=d_{r-protein}(\mu_0)\sum_{j=1}^{19}2^{-m_j^{(r-protein)}C\mu}\Big/\sum_{j=1}^{19}2^{-m_j^{(r-protein)}C\mu_0}$ where $d_{r-protein}(\mu_0)$= $d_{rrn}(\mu_0)$/7, μ_0 = growth rate of WT cell in the given medium, $d_{rrn}(\mu_0)$=$D_{rrn}(\mu_0)/V_{cell}(\mu_0)$ and $D_{rrn}(\mu_0)=\sum_{j=1}^{7}2^{\mu\left(C(1-m^{(rrm)})+D\right)}$. For example, $D_{rrn}(\mu_0$ = 2.5 doub/h) = 36.0 and $D_{rrn}(\mu_0$ = 1.0 doub/h) = 15.1 (also c.f. table 2 of [1] and table 3 of [3]); $D_{r-protein}(\mu_0$ = 2.5 doub/h) = 5.14 and $D_{r-protein}(\mu_0$ = 1.0 doub/h) = 2.16; r-protein map locations are: 74.27, 74.38, 74.15, 74.84, 90.02, 90.05, 71.33, 38.75, 0.45, 20.72, 59.14, 4.09, 95.33, 69.16, 72.77, 49.15, 82.11, 24.71, 83.68 min [2].
[e] Maximum transcription initiation rates for the r-protein and *rrn* gene classes are given by the constitutive and *rrn* promoter classes respectively in tables 1 and 2 of [1]. The maximum transcription initiation rate for the bulk gene class is given by

$V_{bulk}^{\max} = \left(D_r V_r^{\max} + D_{ps} V_{ps}^{\max}\right) / D_{bulk}$ where $V^{max}{}_r$ and $V^{max}{}_{ps}$ are the maximum transcription initiation rates of repressable and pause promoter classes respectively, taken from tables 1 and 2 of [1] and D_{bulk} is defined in footnote d.

[f] The maximum translation initiation rate for the bulk gene class, $U_{bulk}^{\max}$, was chosen to be an arbitrarily value above observed translation initiation rates and below the maximum physical limit set by close packing of ribosomes. See also S1.1.1 and main text.

[g] RNAp holoenzyme-promoter binding affinity for r-protein and for bulk gene classes are given by the constitutive and repressable/pause binding affinities given in tables 1 and 2 of [1]. The binding affinity for the *rrn* gene class, $K_{m,rrn}$, was calculated based on $i_{rrn} = V_{rrn}^{\max} n_{RNAp,free} / \left(n_{RNAp,free} + K_{m,rrn}\right)$, where $n_{RNAp,free}$ is the free RNAp concentration and i_{rrn} is the observed number of initiations per *rrn* operon, given in table 3 of [1]. K_m values are given in units of concentration times the volume of a WT cell in the given medium. To convert to μM: K_m(μM) = K_m(molec per cell)/V_{cell}((μm)^3)/602. V_{cell} is given in Table S2. Note that for constitutive promoters: K_m(1 doub/h; molec/cell) = K_m(2.5 doub/h; molec/cell)V_{cell}(1 doub/h)/V_{cell}(2.5 doub/h).

[h] Ribosome binding affinities were calculated as described in the main text and S1.1.1. To convert to μM use formula given in footnote g. It was assumed that bulk mRNA expression parameters, $U_{bulk}^{\max}$ and $L_{m,bulk}$, are fixed and growth rate independent and were based on estimation at 2.5 doub/h (c.f. S1.1.1). Therefore L_m(1 doub/h; molec/cell)= L_m(2.5 doub/h; molec/cell)V_{cell}(1 doub/h)/V_{cell}(2.5 doub/h).

[i] Since mRNA half-life can alter due to translation-degradation coupling (see main text and S2.7), the actual genetic parameter is not $T_{1/2,i}^{fun}$, but rather the mRNA half-life in the absence of ribosomes, $T_{1/2,i}^{fun,0}$. $T_{1/2,i}^{fun,0}$ is a combination of the Michaelis-Menten parameters of mRNA degradation ($W^{max}{}_i$, $J_{m,i}$) and the mRNA binding affinity of RNase E to its own mRNA, J_1 (c.f. S2.5.2 and S2.7). For mRNA decay via the 5' competition model (Eq. R3 in S2.5.2) this parameter is given by $T_{1/2,i}^{fun,0} = \left[J_{m,i} / \left(J_1 W_i^{\max}\right)\right] \ln 2$ and according to this model it can be calculated from $T_{1/2,i}^{fun,0} = T_{1/2,i}^{fun}(WT)\left(1 + n_{ribo,free}(WT)/L_{m,i}\right)^{-1}$, where $n_{ribo,free}$(WT) is the WT concentration of free ribosomes and $T_{1/2,i}^{fun}(WT)$ is the WT mRNA half-life of the *i*-th gene class (see S2.5.2, S2.6, S2.7 and main text for further details). Determination of $T_{1/2,i}^{fun}$: total intensity of all mRNA in *E. coli* MG1655 in LB broth at 37°C was measured to decay exponentially with a half-life of 6.8 min [5] (the exact growth rate of the culture was not given). This value was taken as the bulk mRNA half-life. This approximation is valid if r-protein half-life is close to the total mRNA half-life. To check this, using data published online (mRNA_half_lives.txt, [5] online), the average mRNA intensity weighed half-life of all genes was 8.6 min (where intensity was approximated by the average difference at t=0, 2-fold method; setting, as the authors, HL>40min to 40min [5]). The average intensity weighted half-life of all r-protein genes was 8.2 min, close to the average mRNA half-life of all genes. For 1 doub/h, $U_{bulk}^{\max}$ and $L_{m,bulk}$ were assumed to be fixed at their value at 2.5 doub/h, and $T_{1/2,i}^{fun}$ was estimated to minimize the error with respect to the wild-type cell state at 1 doub/h (see S1.1.1).

[j] For the r-protein gene class, $L_{r\text{-}protein}$ is taken to be the sum of all r-proteins gene lengths for MG1655. Genes are listed in [6] pp. 60-61. L7 is discarded since it is identical to L12 (only N-acetylated). L26 is discarded since it is identical to S20 and appears only once in the 70S ribosome [6] p. 61. Note that the number of amino acids in a 70S ribosome is slightly higher (=22332/3) since L7/L12 appears in four copies in the final ribosome [6] p. 61. Bulk gene class length is based on tables 1 and 2 of [1] (for comparison, according to [7] the average is 1100 bp). *rrn* gene length is calculated as follows: There are 4566 bp of rRNA in a 70S ribosome (table 1 in [3]); precursor rRNA length is 6000bp (table 1 in [3]); The length of the *rrn* gene class includes transfer RNA (tRNA) coding genes since rRNA and tRNA are stoichiometrically fixed at a ratio of 14:86 at all growth rates (table 1 of [3]; [8]). Although this fixed proportionally appears to be disrupted in perturbed cells [9], we currently do not take this correction into account. Since this gene class includes tRNA transcription and any overhead in the precursor rRNA, the total gene class length is: (total rRNA + total tRNA) + (precursor overhead) = 4566/0.86 + (6000 – 120 – 4566) = 6623bp where 120bp is the average tRNA gene length inside *rrn* operons (which is subtracted because it is already included in the 14% tRNA).

[k] See table 3 in [3]. By redefining c_p for the *i*-th gene class as: $c_{p,i} = c_p / \left(1 + c_p / \left(L_i U_i^{\max}\right)\right)$ one can take into account ribosomes bound to the RBS of the *i*-th gene class mRNA, which was neglected in Eq. 2v. For $U^{max}{}_i$ = 80 ini/min, for example, this correction is quite small (21→20 aa/sec and 16→15.4 aa/sec) but it may be consequential for low values of $U^{max}{}_i$. This correction was included in the simulations for the bulk protein gene class.

[l] r-protein mRNA chain elongation rate and rRNA chain elongation rate are given by the constitutive promoter class mRNA elongation rate and *rrn* promoter class rRNA chain elongation rate respectively in tables 1 and 2 in [1]. Bulk gene class mRNA chain elongation rate is given by $c_{bulk} = D_{bulk} V_{bulk}^{\max}\left(D_r V_r^{\max}/c_r + D_{ps} V_{ps}^{\max}/c_{ps}\right)^{-1}$ where D_{bulk} is defined in footnote d and $V^{max}{}_{bulk}$, $V^{max}{}_r$ and $V^{max}{}_{ps}$ are defined in footnote e and c_r and c_{ps} are the transcription elongation rate for repressable and pause genes respectively defined in tables 1 and 2 of [1]. By redefining c_i to be $c_i' = c_i / \left(1 + c_i / \left(L_i V_i^{\max}\right)\right)$ one can take into account RNAp bound to promoters, which was neglected in Eq. 2iv. This correction is substantial for repressable genes due to their low V^{max}. In the simulations this correction was implemented for the pause and repressable gene classes (c_r and c_{ps}). By correcting c_r and c_{ps} one obtains c_{bulk}=1.36 (3.58) nuc/sec for 2.5 (1.0) doub/h. We note that mRNA chain elongation rate in table 2 of [1] should be 55 nuc/sec and not 45 nuc/sec, due to a misprint.

Table S2. Cell state and additional parameters (in gray) for various growth conditions. Measurements are for *E. coli* at 37°C according to data from [1] table 4 and from [3] tables 2 and 3. Number of molecules per cell are measured as average number per cell, i.e. number per unit of volume of culture divided by the number of cells in that culture, as defined by [3]. To convert to µM: $n(\mu\text{M}) \cong N(\text{molec/cell}) \big/ \left[602 \cdot V_{cell}[\mu m]^3\right]$. Mass and volume of bulk proteins and ribosomes: Bulk protein mass is calculated assuming 108 D/aa (Dalton per amino acid; table 2 in [3]), m_{bulk}=1000 bp/3*108=36 kD. Ribosome mass is calculated to include tRNA, and assuming 324 D/RNA nuc (table 2 in [3]), and with 4566 RNA nuc per 70S ribosome (table 1 in [3]). Thus: m_{ribo}=22332/3*108+4566/0.86*324=2.52MD (where 22332/3 is the number of amino acids present in a 70S ribosome- see Table S1 footnote j). Assuming a macromolecule specific density of 1 ml/g [10] we find that the specific volumes of bulk proteins and ribosomes (including tRNA overhead) are v_{bulk}=5.98 *10^{-8} (µm)^3 and v_{ribo}= 4.18*10^{-6} (µm)^3 respectively.

State variable	Meaning	Units			
μ	Growth rate	doub/h	2.5	2.0	1.0
N_{bulk} [a]	Average number of bulk proteins per cell	molec/cell	$5.76\cdot10^6$	$4.57\cdot10^6$	$2.29\cdot10^6$
N_{RNAp} [c]	Average total number of RNAp core enzymes (mature and immature) per cell	molec/cell	11400	8000	2800
$N_{RNAp,free}$ [b]	Average number of free functional RNAp core enzymes per cell	molec/cell	886	389	144
N_{ribo} [c]	Average total number of ribosomes per cell (mature and immature)	molec/cell	72000	45100	13500
$N_{ribo,free}$ [d]	Average number of free functional ribosomes per cell	molec/cell	4719	4100	1942
V_{cell} [e]	Cell volume	(µm)^3	1.2	0.97	0.63
C [c]	C period	min	42	43	50
D [c]	D period	min	23	24	27

[a] $< N_{bulk} >= (1-\alpha_r-\alpha_p)3\text{P}_c / L_{bulk}$, where P_c is the amount of protein per cell (in amino acid residues) (table 2 in [3]), α_r is the percent of r-protein per total protein (table 3 in [3]), α_p is the percent of RNAp per total protein, (table 3 in [3]) and L_{bulk} is the length of the bulk protein gene class (in base pairs), given in Table S1.

[b] Data for 1 and 2.5 doub/h is taken from [1]. For 2 doub/h see calculation in S1.2. Note that the σ^{70} subunit is present at 20% to 40% of the level of core polymerase [11,12] ($\beta\beta'\alpha_2\sigma^{70}$ is the major form of holoenzyme in exponential growth responsible for transcription initiation of all rRNA, tRNA and most mRNA operons [11]) but it is most likely in excess over free core, and thus newly formed core is expected to be rapidly converted into holoenzyme [12]. This can be seen from the following calculation (also c.f. [12]): by measurement of the β and β' subunit concentration in minicells (minicells are DNA-less portions of bacterial cytoplasm pinched off during aberrant cell divisions in certain bacterial mutants [1]) it has been estimated that the concentration of cytoplasmic β and β' subunits of RNAp is about 17% of the value in whole cells [12]. At 2.5 doub/h the percentage of immature RNAp is about $\left(1-e^{-\alpha\tau_{RNAp}}\right)=8.3\%$ (see Eq. S13) leaving 17%-8.3%=8.7%=991 free core enzymes per cell at this growth rate (also see [1]; the amount of core enzyme is limited by the concentration of β and β' since α subunit is in excess [3]). Since σ^{70} is released prior to the start of RNA chain elongation, the number of σ^{70} bound to promoters has an upper limit which is the number of RNAp molecules bound to the promoter. For 2.5 doub/h this number is estimated to be 216 RNAp molecules (table 2 in [1]). Therefore the number of free σ^{70} units is (20% to 40%)*11400 – 216 = 2064 to 4344 molec/cell, in excess of the number of free core polymerase = 991 molec/cell.

[c] See table 3 in [3]

[d] $N_{ribo,free} = N_{ribo} - N_{ribo,bound} - N_{ribo,imm} = (2^{-\mu\tau_{ribo}} - \beta_r)N_{ribo}$, where β_r is the fraction of active ribosomes (table 3 in [3]), $N_{ribo,bound}$ is the number of ribosomes per cell that are bound to mRNA, τ_{ribo} is the average assembly time of a ribosome (~5 min [3]) and $N_{ribo,imm}$ is the number immature ribosomes per cell (c.f. S2.5.1). Free ribosome concentration at 2.5, 2 and 1 doub/h correspond to 6.5, 7, 5.1 μM respectively. [13] estimated (neglecting nonfunctional ribosome units) an upper bound of 8.5 μM. Also as a comparison, in glucose-amino acid medium, it has been estimated that there are approximately 750 free ribosomes [14]. Note that a free 30S subunit can associate with a free 50S subunit to form a 70S subunit, and only free 30S subunits can initiate translation [6] pp. 203-204. Therefore free 70S ribosomes can in principle sequester free 30S units. However such sequestering is limited by the initiation factor IF3. IF3, binding of which – along with IF1 and IF2 – is a perquisite for subsequent attachment of the 30S subunit to mRNA and fMet-$tRNA_f$, prevents association of the 30S-IF3 complex with the free 50S subunit [6] p. 203, 206. It has been proposed that IF3 (in fact all three factors) are present at high enough concentration to saturate the free 30S subunits [15]. To see how this may be so we note the following: At 2.5 doub/h there is approximately one IF3 molecule per five ribosomes (and this ratio is more or less growth rate independent [16]). At 2.5 doub/h this amounts to 72000/5=14400 IF3 molec/cell. From this we should subtract the number of IF3 molecules bound to mRNA. The number of IF3 molecules bound to mRNA can be estimated as follows: If p is the probability that an mRNA RBS is occupied, then the number of RBSs that engage 30S subunits would be $p \cdot M_{tot}$, where M_{tot} is the total number of mRNA molecules per cell. Since a ribosome occupies the RBS for a time U^{-1}, where U is the average translation initiation rate of total mRNA, and since an IF3 molecule is ejected before peptide chain elongation commences [6] after some average time $T<U^{-1}$, then the average number of IF3 molecules bound at any given time to any mRNA would be $N_{IF3,bound}$, $\sim U \cdot T \cdot p \cdot M_{tot}$, and where U(2.5 doub/h) = 45 ini/min ([1] figure 3d), and $T \sim 30^{-1}$ sec ([6] p.208). $M_{tot} = T_{1/2}^{chem} / \ln 2 \cdot r_{mRNA}$ where r_{mRNA} is the mRNA synthesis rate. At 2.5 doub/h $r_{mRNA}=23.4 \cdot 10^5$ *nucl/min/cell/1000bp=39 mRNA/sec/cell* [3] table 3 (assuming 1000 bp per mRNA). For $T_{1/2}^{chem} \approx 6.8$ min (Table S1), $M_{tot} \approx 23000$ molec/cell and for p~0.5 we have $N_{IF3,bound} \sim 300$ per cell. Therefore the number of free IF3 molecules is ~14400-300=14100 per cell > free 30S subunits per cell ≈ 4700. This noted, it has also been estimated that *in vivo* there are about 8% 70S single (free) ribosomes present [17].

[e] Data for 1 and 2.5 doub/h is taken from [1]. For 2 doub/h volume was interpolated based on the method proposed in [1]: V_{cell}(2 doub/h)=V_M(2 doub/h)*M_C(2 doub/h) where V_M is volume per mass and M_C is mass per cell (in OD_{460} per cell) from [3] table 2. V_M, which changes only moderately with growth rate, was linearly interpolated for 2 doub/h given that V_M(μ=1.33 doub/h)= 0.32 mm^3 per OD_{460} and V_M(μ=2.14 doub/h)=0.25 mm^3 per OD_{460} [1].

S1.1.1 Reconstruction of the wild-type cell state at 1 and 2.5 doub/h

The equation of state are consistent with expected cell state at 2.5 doub/h and prediction of bulk mRNA translation initiation is within the observed range.

The cell state (n_{ribo} , $n_{ribo,free}$, n_{bulk}, n_{RNAp}, $n_{RNAp,free}$ and μ) at 2.5 doub/h is given in Table S2. Note that $n_{ribo,free}$ was estimated based on known fraction of active ribosomes in the cell and an estimate of the number of immature ribosomes (Eq. S13) and is within reasonable agreement with reported estimates (see Table S2 footnote d). As mentioned in the main text, most of the genetic parameters estimated for 2.5 doub/h are based on the genetic parameters of [1] chosen to fit observations of *transcription* in the cell. The only unknown genetic parameters that must be estimated are bulk gene class translation related parameters: $U_{bulk}^{\max}$, $L_{m,bulk}$ and c_{ribo}[1]. To estimate these parameters we shall reverse the problem: given the WT cell state in a rich environment, we will attempt to estimate the unknown genetic parameters. $U_{bulk}^{\max}$ has an upper physical bound of ~115 ini/min (=c_p/d_{min}), where $d_{min} \approx 11$ codons [18] is the minimal distance between two translating ribosomes set to the diameter of a ribosome. Therefore for a given $U_{bulk}^{\max}$ (≤ 115 ini/min), and assuming an arbitrary value for n_0[2], we searched for the optimal $L_{m,bulk}$ and c_{ribo} that minimized the mean square error between the calculated cell state and the given WT cell state. Next we solved the equations of state numerically for the best estimate. The error in estimation of the WT cell state for various values of $U_{bulk}^{\max}$ are given in Table S3 along with the optimal $L_{m,bulk}$ and estimated (arbitrary) cost, c_{ribo}. We find that $U_{bulk} \approx 34$ ini/min. This value can be compared to the average translation initiation rate of *lacZ* ~19 ini/min [18,19] or the average translation initiation rate of total mRNA at 2.5 doub/h, which is ~45 ini/min (c.f. [8] figure 3d). The latter value is expected to be higher than the value obtained here due to the contribution of r-protein mRNAs that are thought to have an efficient RBS [8]. We found that $L_{m,bulk} = 1 \div 10\ \mu$M, depending on the value chosen for $U_{bulk}^{\max}$. It is interesting to note that *in vitro* measurements of 30S-mRNA binding affinities are of the order of 0.1 μM [13], although conclusions based on comparison of these two figures would be tentative since we are not comparing the same mRNAs. Regarding the error in estimation, we note that since it is really the product $T_{1/2,bulk}^{fun} U_{bulk}$ that matters in the equations, any uncertainty in $T_{1/2,bulk}^{fun}$ will be translated into U_{bulk}.

[1] c_{ribo} does not constrain this solution, since for a given WT n_{ribo} and n_{bulk} and a given (small enough) n_0, there is always a c_{ribo} which will satisfy the cost-criterion equation.

[2] At 2 doub/h this parameter was determined by fitting to the experimental data of Squires.

Testing the model at 1 doub/h. Reversing the problem once more, given the WT cell state in a poor medium we can predict the genetic parameters for a poor medium. We will assume that the bulk mRNA is constitutive with fixed expression parameters, using the values obtained for 2.5 doub/h. We will also assume that the concentration n_0 is growth rate independent. We find that in order to reproduce the cell state at 1 doub/h we must allow flexibility in $T^{fun}_{1/2,bulk}$ [3]. The optimal $T^{fun}_{1/2,bulk}$ that minimizes the mean square error with respect to the WT cell state at 1 doub/h are given in Table S3. We see that according to this model, the average functional half-life of bulk mRNA in poor media is reduced by a factor of ~5.3 with respect to its value in rich media. Given the mRNA synthesis rate in the cell [3], this predicts ~1750 mRNAs per cell not very far from a measured value of 1380 at 1.5 doub/h [20]. The reduction in mRNA half-life relative to the rich medium may be due to the fact that different genetic networks are switch on in a poor vs. rich environment. It is also possible that the expectation values assumed for $U^{\max}_{bulk}$ and $L_{m,bulk}$ will drift. Note that we predict $U_{bulk} \approx 30$ ini/min that can be compared with the average rate for total mRNA in *E. coli* at 1 doub/h which is ~20 ini/min.

Table S3. Reconstruction of the WT cell state for 1 and 2.5 doub/h, 37°C. Variables denoted in bold were allowed to vary in order to minimize the MSE with respect to the WT cell state. The error is cell state was not greater than 6%. All concentrations are given in molec/cell, where V_{cell} is 1.2 and 0.63 (μm)^3 at 2.5 and 1 doub/h respectively (Table S2). n_0=2.77 mM. The nonlinear least squares problem was solved using the Levenberg-Marquardt method implemented in Matlab 7. See main text and S1.1.1 for further explanations.

$U^{\max}_{bulk}$ (ini/min)	$L_{m,bulk}$ ($\cdot 10^2$)	c_{ribo}	$T^{fun}_{1/2,bulk}$ (min)	U_{bulk} (ini/min)	μ doub/h	n_{bulk} ($\cdot 10^6$)	n_{ribo} ($\cdot 10^4$)	n_{RNAp} ($\cdot 10^3$)	$n_{RNAP,free}$	$n_{ribo,free}$ ($\cdot 10^3$)
40	**9.57 (1.3 μM)**	**49**	6.8	33	2.5	**5.60**	**7.33**	11.4	**916**	**4.72**
80	**61.4 (8.5 μM)**	**53**	6.8	35	2.5	**5.85**	**7.33**	114	**916**	**4.72**
40	5.03 (1.3 μM)	**85**	**1.2**	32	1	**2.15**	**1.30**	2.8	**143**	**1.94**
80	32.3 (8.5 μM)	**90**	**1.3**	30	1	**2.23**	**1.30**	2.8	**143**	**1.94**

[3] Similar results can be shown if the mRNA half-life is assumed to be constant (=6.8 min) and $L_{m,bulk}$ is allowed to vary.

S1.2 Reconstruction of *E. coli* WT genetic parameters at 2 doub/h

To obtain the genetic parameters of WT *E. coli* at 2 doub/h, we first need to reconstruct the genetic parameters as defined by [1] for WT *E. coli* at 37°C growing at μ=2 doub/h following the logic of those authors. Using these genetic parameters one can calculate the genetic parameters for the gene classes defined in this report. Bulk mRNA half-life was set to 6.8 min (Table S1). Finally $L_{m,bulk}$ and c_{ribo} were estimated (given an arbitrary value of n_0) so that the mean square error with respect to observed WT cell state at 2 doub/h (Table S2) was minimized (implementing the same procedure used to estimate $L_{m,bulk}$ and c_{ribo} for 2.5 doub/h – see main text and S1.1.1). The final genetic parameters obtained at 2 doub/h reflect many different parameters measured or estimated for WT *E. coli* at 2 doub/h, including: promoter strengths of the different gene classes, mRNA and rRNA synthesis rate, cell state, percent of inactive RNAp, average translation initiation rate of total mRNA, mRNA and rRNA elongation rates, tRNA/rRNA ratio, percent of translating ribosomes, RNAp and ribosome assembly times, the C period and *E. coli*'s genome (gene map locations and gene lengths). In order to estimate the genetic parameters at 2 doub/h we first have to reproduce the transcription related parameters as defined by [1] for 2 doub/h based on the rational of [1]. These parameters are given in Table S4.

Table S4. Transcription related parameters for 2 doub/h, 37°C. Transcription related parameters as defined in [1], obtained here for 2 doub/h. Values in bold were estimated. See S1.2 for further explanations.

Genetic parameter	Units	P1 [a]	P2 [a]	constitutive [a]	repressable [a]	pause [a]
$D_i = V_{cell}(2\text{ doub/h}) \cdot d_i$	average copies per cell	27 [b]	27 [b]	40.4 [c]	**408.4** [d]	161.8 [e]
$V_i^{\max}$	ini/min	110 [f]	110 [f]	33 [g]	1.5 [h]	3.3 [i]
c_i	nuc/sec	85 [j]	85 [j]	52 [j]	52 [j]	**0.89** [d]
$K_{m,i}$	molec/cell	1240 [l]	2531 [k]	405 [k]	405 [k]	405 [k]
L_i	base pairs	6000 [m]	6000 [m]	2000 [m]	1000 [m]	1000 [m]

[a] Promoter classes as defined by [1].
[b] The average number of copies of the *rrn* operon per cell, $D_{rrn}(\mu)$, is given by Eq. S7: $D_{rrn}(\mu) = \sum_{j=1}^{7} 2^{\mu\left(C(1-m_j^{(rrn)})+D\right)}$, where $m_j^{(rrn)}$ are the *rrn* operon map locations given in Table S1.
[c] According to [1], $[P_{constitutive}]=1.5[P_{rrn}]$, in order to fit to (1) transcription of all r-proteins and (2) mRNA synthesis rate. This is consistent with the length of the r-protein gene class given in Table S1: the total DNA per chromosome associated with this gene class is roughly 40.4/(27/7=gene dosage per gene)*(2000 bp) = 20,948 = total length of constitutive class coding genes, compared with $L_{r\text{-}protein}$= 21252 (Table S1).
[d] Estimated value - see S1.2.
[e] According to [1], based on known fractions of intermittently inactive RNAp in the cell $[P_{pause}] = 6[P_{rrn}]$.
[f] Estimated in [8]. Assumed to be growth rate independent [1].
[g] As measured for the spc ribosomal promoter, which is a representative promoter for this class (see [1]).
[h] As measured for β-lactamase promoter, which is taken to be a representative promoter for this class (see [1]).
[i] Maximum initiation rate was set to be ten fold lower than the V^{max} for the constitutive gene class, assuming pause genes are blocked 90% of the time [1].
[j] See table 3 in [3].
[k] Constitutive promoter binding affinities scale according to cell volume with respect to their values at 2.5 doub/h and are taken from [1]. Volumes are given in Table S2.
[l] Taken from table 5 of [21]: K_{P1}:K_{P2} at (2.14 doub/h) = 0.49:1, i.e. 1240=0.49*2531.
[m] See tables 1 and 2 in [1].

There are two remaining genetic parameters that need to be fixed: The average number of repressable promoters per cell, $D_{repressable}$, and mRNA chain elongation rate of pause genes c_{pause}. To find the best solution we searched for $D_{repressable}$ and c_{pause} that while satisfying the RNAp conservation equation (Eq. S4) minimized the error in estimation of rRNA synthesis rate r_{rRNA} (Eq. S2) and mRNA synthesis rate r_{mRNA} (Eq. S3). r_{rRNA}=0.86*r_s(μ=2 doub/h), where r_s is the stable rRNA synthesis rate (see footnote c in table 3 of [1]). n_{RNAp} is given in Table S2 and $n_{RNAp,free}$ was a variable that was solved for. sRNA and mRNA synthesis rates are given in table 3 of [3], and equations S1-S4 are given in [1]. Note that all concentrations were multiplied by the average cell volume at 2.0 doub/h so that concentration is given in units of molec per cell. Minimization was preformed using Matlab's nonlinear least squares optimization routine that implements the trust-region reflective Newton method.

$$V_i = p_i V_i^{\max}\left(\frac{1}{1+K_{m,i}/n_{RNAp,free}}\right)$$
$$V_i' = V_i / p_i \tag{S1}$$
$$p_i \triangleq \begin{cases} 1 & \text{i=1,3,4,5} \\ p_{ocl} & \text{i=2} \end{cases} \quad \text{where } p_{ocl} = e^x, x = -70/(5100/V_1' - 50)$$

$$r_{rRNA} \equiv r_s = \sum_{i=1}^{2} D_i L_i V_i \tag{S2}$$

$$r_{mRNA} \equiv r_m = \sum_{i=3}^{5} D_i L_i V_i \tag{S3}$$

$$n_{RNAp} = n_{RNAp,free} + n_{RNAp,immature} + \sum_{i=1}^{5} D_i\left(1+\frac{L_i}{c_i}V_i^{\max}\right)\left(\frac{1}{1+K_{m,i}/n_{RNAp,free}}\right) \tag{S4}$$
$$\text{where } n_{RNAp,immature} = n_{RNAp}(2^{t/\tau}-1)$$

Where i=1 P1; i=2 P2; i=3 constitutive; i=4 repressable; i=5 pause. To check our estimate we calculated the number of active (bound and transcribing) RNAp molecules per cell (Eq. S5) and the *rrn* P1-P2 initiation rate (Eq. S6). Experimental values are given for these parameters in table 3 of [3].

$$R_{b,elong} \triangleq \sum_{i=1}^{4} D_i \frac{L_i}{c_i} V_i^{\max}\left(\frac{1}{1+K_{m,i}/n_{RNAp,free}}\right) \tag{S5}$$

$$i_{rrn} \equiv V_1 + V_2 \tag{S6}$$

where Eq. S5 and S6 are from [1]. The results for this optimization are given below:

```
cpause=0.888 nuc/sec
Drepressable=408.44 genes/cell
NRNAp,free =389 molec/cell
```

Compare to experiment:

```
rRNAsynthRate= 5.7104e+006 vs. observed=5.7104e+006. Error=3.21e-010 %
mRNAsynthRate = 1.87e+006 vs. observed=1.87e+006. Error=2.476e-010 %
```

Additional experimental information:

```
BoundElongRNAp = 1814.2 vs. observed=1929. Error=-5.96 %
i_rrn=35.3017 vs. observed=39. Error=10.48 %
# of pausing RNAp per gene = 30.86
Percent inactive RNAp=61.41 %
```

The estimated c_{pause}(2 doub/h)=0.89 nuc/sec is slightly lower than the estimated value of [1] for 1 doub/h (c_{pause}=1 nuc/sec) and for 2.5 doub/h (c_{pause}=1.3 nuc/sec). However, repeating this algorithm for 1 doub/h yields also c_{pause}(1 doub/h)=0.89 nuc/sec and with much lower errors for mRNA and rRNA synthesis rates (essentially 0) compared with the respective errors reported in table 3 of [1] for 1 doub/h: (1.09-0.99)/0.99=10.1% for stable RNA synthesis rate and (0.99-0.91)/0.91=8.9% for mRNA synthesis rate. The error in predicting active RNAp and *rrn* initiation rate is 6% and 10.5% respectively, compared, for example, with the respective errors of (0.3-0.29)/0.3=3.3% and (58-50)/58=13% obtained by [1] for 2.5 doub/h.

The following table summarizes the CGGR genetic parameters at 2 doub/h.

Table S5. Genetic parameters for *E. coli* growing at 2 doub/h, 37°C. See also footnotes in Table S1 and S1.2 for further explanations.

Gene class		Units	r-protein	bulk	*rrn*
m [h]	Map location	MU (min)	see footnote	191 uniformly distributed genes	see footnote
$V_i^{\max}$	Maximum transcription initiation rate	ini/min	33 [a]	2.01 [d]	110 [a]
$U_i^{\max}$	Maximum translation initiation rate	ini/min	-	80 [a]	-
$K_{m,i}$	Promoter-RNAp holoenzyme binding affinity	molec/cell	405 [c]	405 [c]	708 [i]
$L_{m,i}$	RBS-30S ribosome subunit binding affinity	molec/cell	-	13261 [g]	-
$T_{1/2,i}^{fun}$	mRNA half-life	min	-	6.8 [e]	-
L_i	Gene class length	base pairs	21252 [a]	1000 [a]	6623 [a]
c_p	Peptide chain elongation rate	aa/sec	20 [b]	20 [b]	-
c_i	RNA chain elongation rate	nuc/sec	52 [b]	1.87 [f]	85 [b]

[a] See footnote e Table S1. r-protein and *rrn* maximum transcription initiation rates are given in Table S4.

[b] See table 3 in [3]. By redefining c_p to include 30S subunits bound to the RBS we obtain 20→19.1 aa/sec. Also see footnote k in Table S1.

[c] K_m(2 doub/h; molec/cell) = K_m(2.5 doub/h; molec/cell)V_{cell}(2 doub/h)/V_{cell}(2.5 doub/h), where values for K_m and V_{cell} are taken from Tables S1 and S2 respectively.

[d] V^{max} for the bulk promoter is calculated according to data from Table S4- see footnote e in Table S1 for formula.

[e] Based on total mRNA half-life measurement for LB broth at 37°C [5] (see Table S1 footnote i).

[f] Bulk mRNA chain elongation rate, c_{bulk}, was calculated according to data from Table S4- see footnote l in Table S1 for formula. By redefining c_r and c_{ps} to include RNAp bound to the promoter we obtain c_{bulk}=1.78 nuc/sec. See footnote l, Table S1.

[g] 30S ribosome subunit binding affinity was estimated by finding the c_{ribo} and $L_{m,bulk}$ that minimize the mean square error between the predicted and observed WT cell state at 2 doub/h (Table S2), given n_0 = 2.80*10^6 molec/WT cell (see S1.1.1 for example at 2.5 doub/h). The estimated cost was $c_{ribo} \approx 38$ bulk protein per ribosome. n_0 was chosen so that the predicted cost is the cost that gives the best fit for the data of Asai et al.. See main text for further explanations regarding c_{ribo}.

[h] r-protein and *rrn* map locations are given in Table S1. The number of bulk genes was calculated as explained in footnote d of Table S1 (with D_r and D_{ps} for the calculation of D_{bulk} given in Table S4). Gene concentrations are calculated according to the formulae given in Table S1 footnote d with μ_0=2.0 doub/h, D_{rrn} (2 doub/h) = 27 copies per cell (Table S4), $D_{r\text{-}protein}$(2 doub/h) = 27/7 copies per cell (c.f. Table S4) and D_{bulk}(2 doub/h) ≅ 571 copies per cell.

[i] The binding affinity for the *rrn* gene class, $K_{m,rrn}$, was calculated as explained in Table S1 footnote g, where free RNAp concentration, $n_{RNAp,free}$, is given in Table S2 and i_{rrn}, the number of initiations per *rrn* operon for 2 doub/h, is given in table 3 of [1].

WT genetic parameters were obtained for 2 doub/h

Most wild-type parameters have been obtained from measurements at either 2 doub/h (the growth rate of the WT strain) or at growth rates close to this value. The remaining parameters have been calculated for 2 doub/h (e.g. gene concentrations are calculated based on the Cooper-Helmstetter equation for 2 doub/h, and the bulk protein binding affinity is calculated based on the cell state measured at 2 doub/h). mRNA half-life was measured for cells grown in LB broth at 37°C [5] (the same conditions as the inactivation strains) but the exact growth rate was specified. We note however that since only the multiplication of the bulk mRNA half-life and the bulk translation rate appear in the model, any possible error was somewhat compensated by solving for the WT binding affinity leading to the translation rate needed to reproduce the cell state at 2 doub/h. Therefore the genetic parameters in Table S5 should be applicable to a wild-type cell growing at 2 doub/h.

S1.3 Genetic parameters of *rrn* inactivation strains at 2 doub/h

For each inactivation strain we define one gene class representing all functional *rrn* operons and a separate gene class for each inactivated operon. The first task is therefore to determine the genetic makeup of each mutant.

<u>Linage of *rrn* inactivation strains</u>

In most rRNA inactivation instances, the rRNA promoter was left intact but a large part of the rRNA (and tRNA) coding region was removed. The exception is *rrnE* (in Δ2-Δ6) which was completely deleted. A promoterless *cat* gene which expresses chloramphenicol acetyl transferase (product being 219 aa, ca. 24kD) was introduced into one of the rRNA operons (depending on the strain) in the Δ1-Δ6 strains. *lacZ* (product being 1025 aa, ca. 111kD) was also expressed by *rrnG* in Δ5-Δ6.

We assumed that all *rrn* operons have the same promoter constants. In support of this assumption it has been shown that the promoter activity is more or less correlated with distance from the origin (since copies closer to oriC have a higher copy number and thus have a higher level of expression) with the exception of *rrnB* and *rrnH* which give lower expression than expected [2,22]. The table below traces the lineage of all seven inactivation strains used in the experiment and indicates the length of each inactivated *rrn* operon that was transcribed, and in cases where the inactivated operon expressed a protein (*cat* or *lacZ* insertions), the length of the translated portion is also specified.

Table S6. Lineage of the *rrn* inactivation strains. The following table traces the lineage of all *rrn* inactivation strains and indicates the length of each inactivated *rrn* operon that was transcribed, and in cases where the inactivated operon expressed a protein (*cat* or *lacZ* insertions), the length of the translated portion is also specified. A *Δn* strain indicates that *n* operons were inactivated. See S1.3 for further details.

Δ0 = *rrn*$^+$ =TA563	source: ΔTX11 from [23]

Δ1 = ΔAc = TA566	
**A transcription**: **2147bp** (with P_1P_2-CAT fusion) [4]. **translation**: cat$^+$: **657bp**	source : P_1P_2-CAT fusion BAG1 in [4][5]

Δ2 = ΔEAc = TA567	
**E** Completely deleted	source TXΔ11 [23]. See also fig. 1 in [26]
**A transcription**: **2147bp** (with P_1P_2-CAT fusion; see remarks in Δ1) . **translation**: cat$^+$: **657bp**	source : P_1P_2-CAT fusion BAG1 in [4]

Δ3 = ΔEBAc = TA568	
**E** Completely deleted	source TXΔ11 [23]. See also fig. 1 in [26]
**B transcription**: 2287(PCR:16S-23S)+119(5S) [6] =**2406 bp** [7]	source: pMA101, SalI-SalI deletion [22]
**A transcription**: **2147bp** (with P_1P_2-CAT fusion; see remarks in Δ1) . **translation**: cat$^+$: **657bp**	source : P_1P_2-CAT fusion BAG1 in [4]

Δ4 = ΔEBHAc = TA430	
**E** Completely deleted	source TXΔ11 [23]. See also fig. 1 in [26]
**B transcription**: 2287(PCR:16S-23S)+119(5S)=**2406 bp** (see remarks in Δ3)	source: pMA101, SalI-SalI deletion [22]
**H transcription**: 1290(PCR:16S-23S) + 248(5S+distal tRNA) = **1538 bp** [8]	source: pMA103, SacII-SacII deletion [22]
**A transcription**: **2147bp** (with P_1P_2-CAT fusion; see remarks in Δ1) . **translation**: cat$^+$: **657bp**	source : P_1P_2-CAT fusion BAG1 in [4]

Δ5 = ΔEBHGzAc = TA476	
**E** Completely deleted	source TXΔ11 [23]. See also fig. 1 in [26]
**B transcription**: 2287(PCR:16S-23S)+119(5S)=**2406 bp** (see remarks in Δ3)	source: pMA101, SalI-SalI deletion [22]
**H transcription**: 1290(PCR:16S-23S) + 248(5S+distal tRNA) = **1538 bp** (see remark in Δ4)	source: pMA103, SacII-SacII deletion [22]
**G transcription**: 2928(16S-23S) + 3075(*lacZ*$^+$)+212(5S) = **6215** [9]. **translation**: *lacZ*$^+$ **3075**	source pNY30, smaI-HpaI deletion in [22]
**A transcription**: **2147bp** (with P_1P_2-CAT fusion; see remarks in Δ1) . **translation**: cat$^+$: **657bp**	source : P_1P_2-CAT fusion BAG1 in [4]

Δ6 = ΔEBHGzADc = TA516	
**E** Completely deleted	source TXΔ11 [23]. See also fig. 1 in [26]
**B transcription**: 2287(PCR:16S-23S)+119(5S)=**2406 bp** (see remarks in Δ3)	source: pMA101, SalI-SalI deletion [22]
**H transcription**: 1290(PCR:16S-23S) + 248(5S+distal tRNA) = **1538 bp** (see remark in Δ4)	source: pMA103, SacII-SacII deletion [22]
**G transcription**: 2928 (16S-23S) + 3075(*lacZ*$^+$)+212(5S) = **6215** (see remark in Δ5) . **translation**: *lacZ*$^+$ **3075**	source pNY30, smaI-HpaI deletion in [22]
**A transcription**: 1290 (*rrnH* PCR:16S-23S) + 213(5S) = **1503bp**[10]	source: pNY34, SacII-SacII deletion [22]
**D transcription**: **2147bp** (with P_1P_2-CAT fusion) . **translation**: cat$^+$: **657bp**	source: W1485ΔD in [9][11]

---

4 Measured from figure 1 in [4].

5 Linage is traced as follows:

(i) BAG1 from [4] is designated CC164 in [9] and the A was inserted into the final strain.

(ii) A originated from the A1 strain of [4].

(iii) A1 was taken from [22] where it was designated W1485ΔA. W1485ΔA was formed by cutting *rrnA* at the first smaI restriction enzyme site (see figure 1 in [9]) and attaching to it a promoterless CAT gene from plasmid pKK232-8 [24]. pKK232-8 was also cut at smaI ([22] p. 4184, [24] p. 157). This is the first smaI site (see figure 1 in [9]) since according to [4] the CAT was inserted at position 612 bp from the start of 16S. According to figure 1 of [9] the first smaI is located at 605 bp position (assuming WT 16S-23S is 4719 bp [9]). This confirms that the scaling for this figure is correct.

(iv) pKK232-8 was designed by [24] and they used a CAT cassette based on [25].

(v) In [25] it is explained that CAT are derivatives of the Cm^r gene of the bacterial transposon Tn9 and contains the complete CAT polypeptide coding sequence. According to [25] this is 660 bp. This is confirmed in NCBI site for pKK232-8 (=657bp). Next we need to ascertain the length of the transcribed segment. The actual length of the CAT cassette in pKK232-8 is longer then 660 bp since (a) the coding region actually taken in [25] was 780bp and (b) pHH232-8 was cut at smaI which is upstream of the CAT cassette (figure 7 in [24]). Since the exact location of smaI was uncertain, the length of the transcribed gene was estimated from figure 1 in [4].

6 Measured from end of 23S

7 PCR length from [9] + 5S length taken from the NCBI site**.**

8 PCR length from [9] + 5S+tRNA length taken from the NCBI site.

9 Assuming a length of 4876 bp for the 16S-23S segment of *rrnG* (NCBI site) and from scaling of figure 1 in [9]. The first smaI was chosen, as described in figure 1 of [26]. LacZ length is from EcoCyc site, 5S length is from the NCBI site.

10 Assumed to be like *rrnH* PCR fragment since the 16S-23S sequence length of *rrnA* and *rrnH* are both 4892 bp and the same restriction enzymes were used; 5S length was taken from the NCBI site.

11 W1485ΔD in [9] was obtained by the same procedure as W1485ΔA described above: a promotorless CAT gene was inserted into the first smaI site at 612 bp; (see Δ1).

Modeling of the *rrn* inactivation strains

Each inactivation strain was modeled separately based on the genetic makeup of that strain, changing the genetic nature of the inactivated operons while "core parameters" remain the same. Below we explain what these core parameters are and why they were considered fixed in the inactivation strains. Since ppGpp and NTP concentrations are constant for the inactivation strains [4,27], the Michaelis-Menten parameters for rRNA transcription should not be altered [8]. r-protein Michaelis-Menten transcription parameters are constitutive [2] and therefore should also remain constant. Since the growth medium is fixed, we do not expect a significant shift in the pattern of bulk genes being expressed. Also, since the concentration of ppGpp is constant, ppGpp induced pausing [1], which would affect the pause gene class (Table S4) and thus the bulk gene class, is not expected to occur. Thus bulk protein parameters are expected to be more or less fixed as well. Parameters which directly depend on growth rate, and will change in the inactivation strains, like gene concentration (Eq. 3), are taken into account by the model. The effect of translation-degradation coupling on mRNA half-lives (i.e. increased mRNA half-life when the ribosome binding site (RBS) is occupied thereby preventing binding of the endonuclease - [28,29]) was taken into account in the kinetic model by assuming that only mRNA with a vacant RBS can be degraded (c.f. S2.7). The elongation rate for non rRNA genes has been shown to be constant in the inactivation strains [4]. The rRNA elongation rate was shown to be constant for strains with increased rRNA gene dosage [30] and increases in the inactivation strains [4]. For simplicity, in Fig. 2 and Fig. 3 the rRNA elongation rate was assumed to be constant. For Fig. 4, relevant to the discussion of the feedback response, rRNA elongation rate was assumed to increase linearly for the inactivation strains so as to match measured values for this parameter [4]. The C period and peptide chain elongation rates, appearing in Eq. 3 and Eq. 2v respectively, were also assumed to be constants. The change in the C period between 2 doub/h and 1 doub/h ($\approx$growth rate of the Δ6 strain) corresponds to a <10% error in gene concentration according to Eq. 3 for m=1 (and the difference decreases for smaller values of m). It has also been suggested that the C period, as well as the D period, are "regulated such that their values are relatively independent of the cell environment" [31]. The peptide chain elongation reduces by 20% when going from a rich medium (2 doub/h) to a poorer medium (1 doub/h). Although it is not entirely understood why this happens, it has been suggested that this is due to the fact the in a poor environment, peptide chain elongation becomes limited by the extent of tRNA charging [3], and therefore this effect is not expected to occur when the medium is unlimited and is fixed. As for the *lac*Z and *cat* genes that were inserted into some of the inactivated operons, *lac*Z translation initiation rate is approximately growth rate independent ([8], figure 4.2c) and was estimated based on measurements at 1.25 doub/h, 37°C [19] (see below). *lacZ* binding affinity was

estimated based on this value. *lacZ* mRNA half-life was also measured for these conditions [19]. *cat* translation parameters were assumed to be identical to those of *lac*Z. *cat* mRNA half-life depends on the growth medium but apparently not on the growth rate [32,33] and its value was taken from a measurement by [4] for LB glucose medium at 37°C.

<u>*cat* and *lacZ* half-life, translation initiation frequency and costs</u>

cat mRNA half-life was measured to be 1.75 min (presumably at 2.49 doub/h LB glucose medium 37°C) [4]. This half-life depends on the growth medium but apparently not on growth rate [32,33]. *lac*Z mRNA half-life is 1.5 min at 1.25 doub/h 37°C [19] (also functional half-life 1.3 min in M9 + .4% glucose 37°C [34]).

U_{lacZ}= 18.75 ini/min measured at 1.25 doub/h 37°C [19] and is approximately growth rate independent ([3] figure 2c). Assuming $U_{lacZ}^{\max} = 80$ ini/min then at 2 doub/h:

$L_{m,lacZ} = n_{ribo,free}\left(\frac{U_{lacZ}^{\max}}{U_{lacZ}} - 1\right) = 4100\left(\frac{80}{18.75} - 1\right) = 13393$ molec/cell (similar to the bulk RBS binding affinity)

cat translation parameters $U_{cat}^{\max}$ and $L_{m,cat}$ were assumed to be identical to *lacZ* parameters.

For the unconstrained Φ model, the cost of *lac*Z and *cat* were assumed to scale according to the mass of their product relative to the ribosome cost, including contribution of precursor rRNA. For example, $c_{lacZ} = c_{ribo} \cdot m_{lacZ} / m_{ribo\ with\ overhead}$.

<u>Genetic parameters of the seven *rrn* inactivation strains</u>

For each inactivation strain, the map locations for the functioning *rrn* operons that comprise the *rrn* gene class were updated. In addition, we defined a new gene class for every *rrn* operon that was inactivated. The following set of tables summarizes the genetic parameters for the seven inactivation strains. The gray boxes indicate the fields that have been changed from one strain to the next. Note that we distinguish between the length of a gene class that is transcribed and the length of a gene class that is translated. r-protein and bulk gene class genetic parameters remain the same for all strains.

Table S7. **Genetic parameters for the *rrn* inactivation strains at 2 doub/h, 37°C.** Strain names are indicated in bold. A *Δn* strain indicates that *n rrn* operons were inactivated. The table for the Δ0 (i.e. wild-type) strain is simply Table S5. Changes with respect to the previous strain are denoted in gray. See S1.3 for further details.

Δ0 = ΔTX11

**Gene class**		**Units**	**r-protein**	**bulk**	***rrn***
m	Map location	MU (min)	see Table S5		C(84.5), D(72.1), G(56.1), H(5.1), B(89.8), E(90.5), A(86.5)
V_i^{max}	Maximum transcription initiation rate	ini/min	33	2.01	110
U_i^{max}	Maximum translation initiation rate	ini/min	-	80	-
$K_{m,i}$	Promoter-RNAp holoenzyme binding affinity	molec/cell	405	405	708
$L_{m,i}$	RBS-30S ribosome subunit binding affinity	molec/cell	-	13261	-
$T_{1/2,i}^{fun}$	mRNA half-life	min	-	6.8	-
L_i transcr.	Gene class length transcribed	base pairs	21252	1000	6623
L_i transl.	Gene class length translated	base pairs	21252	1000	0
c_p	Peptide chain elongation rate	aa/sec	20	20	-
c_i	RNA chain elongation rate	nuc/sec	52	1.87	85

Δ1 = ΔAc = TA566

**Gene class**	***rrn***	**Load 1**
m	C(84.5), D(72.1), G(56.1), H(5.1), B(89.8), E(90.5)	A(86.5)
V_i^{max}	110	110
U_i^{max}	-	80
$K_{m,i}$	708	708
$L_{m,i}$	-	13261
$T_{1/2,i}^{fun}$	-	1.75
L_i transcr.	6623	2147
L_i transl.	0	657
c_p	-	20
c_i	85	85

Gene class		*rrn*	Load 1	Load 2
m		C(84.5), D(72.1), G(56.1), H(5.1), B(89.8)	A(86.5)	E(90.5)
$V_i^{\max}$		110	110	110
$U_i^{\max}$		-	80	-
$K_{m,i}$	**Δ2 = ΔEAc = TA567**	708	708	708
$L_{m,i}$		-	13261	-
$T_{1/2,i}^{fun}$		-	1.75	-
L_i transcr.		6623	2147	0
L_i transl.		0	657	0
c_p		-	20	-
c_i		85	85	85

Gene class		*rrn*	Load 1	Load 2	Load 3
m		C(84.5), D(72.1), G(56.1), H(5.1)	A(86.5)	E(90.5)	B(89.8)
$V_i^{\max}$		110	110	110	110
$U_i^{\max}$		-	80	-	-
$K_{m,i}$	**Δ3 = ΔEBAc = TA568**	708	708	708	708
$L_{m,i}$		-	13261	-	-
$T_{1/2,i}^{fun}$		-	1.75	-	-
L_i transcr.		6623	2147	0	2406
L_i transl.		0	657	0	0
c_p		-	20	-	-
c_i		85	85	85	85

Gene class		*rrn*	Load 1	Load 2	Load 3	Load 4
m		C(84.5), D(72.1), G(56.1)	A(86.5)	E(90.5)	B(89.8)	H(5.1)
$V_i^{\max}$		110	110	110	110	110
$U_i^{\max}$		-	80	-	-	-
$K_{m,i}$	**Δ4 = ΔEBHAc = TA430**	708	708	708	708	708
$L_{m,i}$		-	13261	-	-	-
$T_{1/2,i}^{fun}$		-	1.75	-	-	-
L_i transcr.		6623	2147	0	2406	1538
L_i transl.		0	657	0	0	0
c_p		-	20	-	-	-
c_i		85	85	85	85	85

Gene class		*rrn*	Load 1	Load 2	Load 3	Load 4	Load 5
m	**Δ5 = ΔEBHGzAc = TA476**	C(84.5), D(72.1)	A(86.5)	E(90.5)	B(89.8)	H(5.1)	G(56.1)
$V_i^{\max}$		110	110	110	110	110	110
$U_i^{\max}$		-	80	-	-	-	80
$K_{m,i}$		708	708	708	708	708	708
$L_{m,i}$		-	13261	-	-	-	13393
$T_{1/2,i}^{fun}$		-	1.75	-	-	-	1.5
L_i transcr.		6623	2147	0	2406	1538	6215
L_i transl.		0	657	0	0	0	3075
c_p		-	20	-	-	-	20
c_i		85	85	85	85	85	85

Gene class		*rrn*	Load 1	Load 2	Load 3	Load 4	Load 5	Load 6
m	**Δ6 = ΔEBHGzADc = TA516**	C(84.5)	A(86.5)	E(90.5)	B(89.8)	H(5.1)	G(56.1)	D(72.1)
$V_i^{\max}$		110	110	110	110	110	110	110
$U_i^{\max}$		-	-	-	-	-	80	80
$K_{m,i}$		708	708	708	708	708	708	708
$L_{m,i}$		-	-	-	-	-	13393	13261
$T_{1/2,i}^{fun}$		-	-	-	-	-	1.5	1.75
L_i transcr.		6623	1503	0	2406	1538	6215	2147
L_i transl.		0	0	0	0	0	3075	657
c_p		-	-	-	-	-	20	20
c_i		85	85	85	85	85	85	85

S1.4 Genetic parameters of strains with increased *rrn* gene dosage

When *rrn* gene dosage was increased beyond seven copies per chromosome, additional *rrn* operons were added at the origin (m=0). All other genetic parameters were assumed to be identical to those of the WT strain (Table S5). A discussion of why these genetic parameters can be assumed to remain unchanged is given in S1.3.

S1.5 Mean square errors with respect to the Squires data

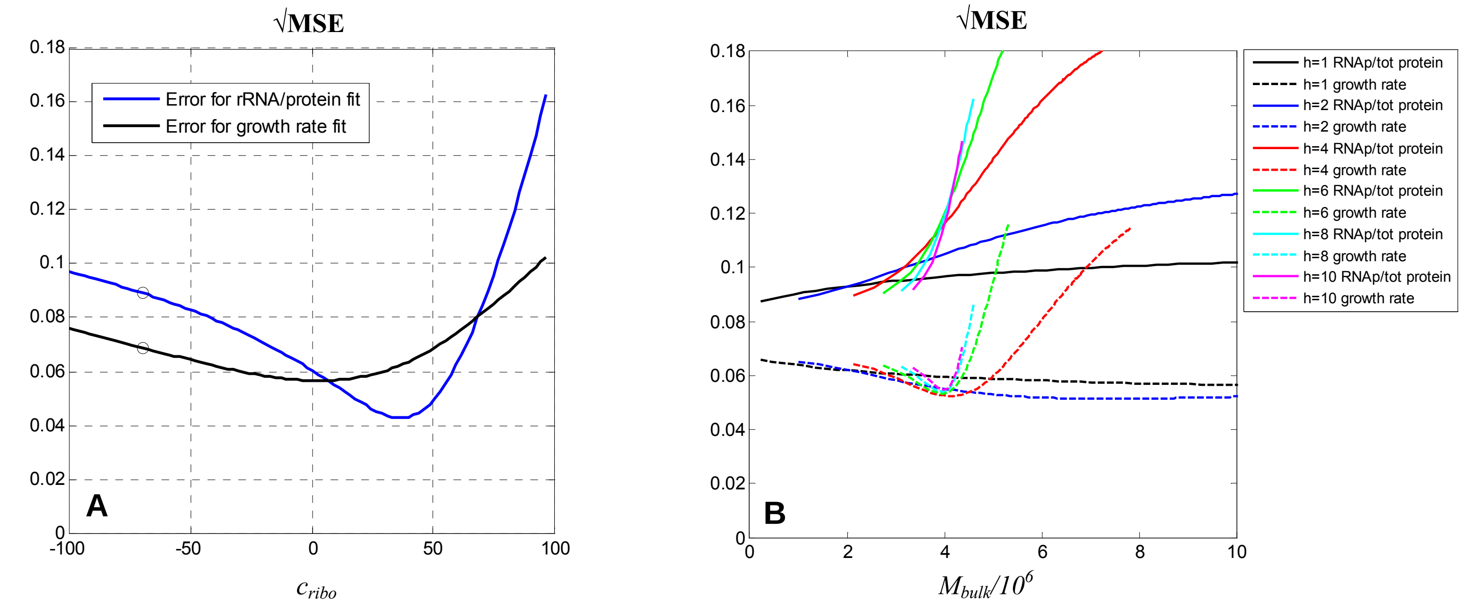

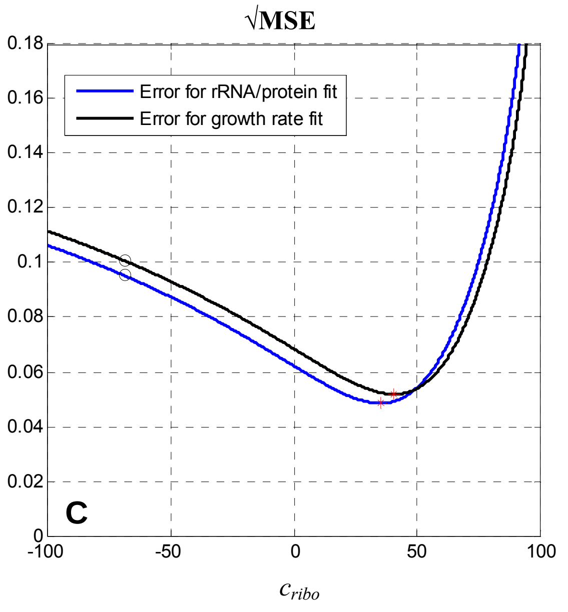

Figure S1. Mean square errors with respect to the Squires data. (A) Unconstrained CGGR MSE. Square root of the mean square error (MSE) as a function of c_{ribo} in estimation of the growth rate and the rRNA to total protein ratio measured by Asai et al. [9]. This graph was computed as follows: for a given n_0, optimal $L_{m,bulk}$ and c_{ribo} that minimize the square error between an estimated WT cell state and the observed WT cell state were obtained (see S1.1.1). Next, for those optimal $L_{m,bulk}$ and c_{ribo} values, the growth rate curve and the rRNA/total protein curve were calculated for the various *rrn* inactivation strains (c.f. S1.3) and the MSEs were calculated between these two curves and the data points, yielding two errors for a given n_0 (or equivalently c_{ribo}). Next, n_0 is increased and the process is repeated. The minimum MSE for the rRNA to total protein ratio (which displayed more sensitivity to c_{ribo} than the growth rate) was obtained for c_{ribo}=37.6 (n_0=2.8·10^6 molec/WT cell). Circles mark the cost for which Φ would be fixed in an unconstrained CGGR model (i.e. when $c_i = -v_i / v_{bulk}$, which is equivalent to the constrained CGGR model with *h=0*). **(B) Constrained CGGR MSE.** Square root of the MSE in estimation of the growth rate and the rRNA to total protein ratio as a function of M_{bulk} and the Hill coefficient *h*, for a model where Φ is assumed to be fixed, and $c_p = c_p^{\max} \big/ \left[1+\left(M_{bulk}/n_{bulk}\right)^h\right]$. This graph was computed as follows: for a given $c_p^{\max}$ and *h*, optimal $L_{m,bulk}$ and M_{bulk} that minimize the square error between the estimated WT cell state and the observed WT cell state were obtained. Note that this square error included the error between the estimated WT c_p and the observed WT value of c_p at 2 doub/h (20 aa/sec). The error in prediction of the WT cell state was on the order of a few percent (data not shown). Next, for those optimal $L_{m,bulk}$ and M_{bulk} values, the growth rate curve and the rRNA/total protein curve were calculated for the various *rrn* inactivation strains and the MSE was calculated between these curves and the data points. Next, $c_p^{\max}$ is increased and the process repeated. The minimum Hill coefficient to yield a solution that did not diverge in growth rate for high *rrn* copy numbers was *h*=2 (see e.g. Fig. S2 for fit with *h*=1). For *h*=2, M_{bulk} was chosen to minimize the growth rate error yielding: M_{bulk}=7.4·10^6 molec/WT cell ($c_p^{\max} = 73$ aa/sec). Solutions that minimized the rRNA/total protein MSE (corresponding to the minimum possible value for $c_p^{\max}$, i.e. $\cong 21$ aa/sec) diverged in growth rate for copy numbers greater than 7 (see Fig. S3). In addition, the MSE did not improve for higher Hill coefficients, as shown. Note that the minimization procedure in (A) and (B) are equivalent if we map $M_{bulk} \leftrightarrow c_{ribo}$, $c_p^{\max} \leftrightarrow n_0$. **(C) Simplified 3-state model MSE.** Square root of the MSE in estimation of the growth rate and the rRNA to total protein ratio as a function of c_{ribo} for the simplified model. Stars indicate minima. Circles indicate the same as in (A). The minima almost coincide and were obtained for $c_{ribo} \simeq 38.2 \pm 2.8$. In both (A) and (B), as in Fig. 2 to Fig. 4, $U_{bulk}^{\max}$ was set to 80 ini/min and the rRNA chain elongation rate, c_{rrn}, was assumed to be constant.

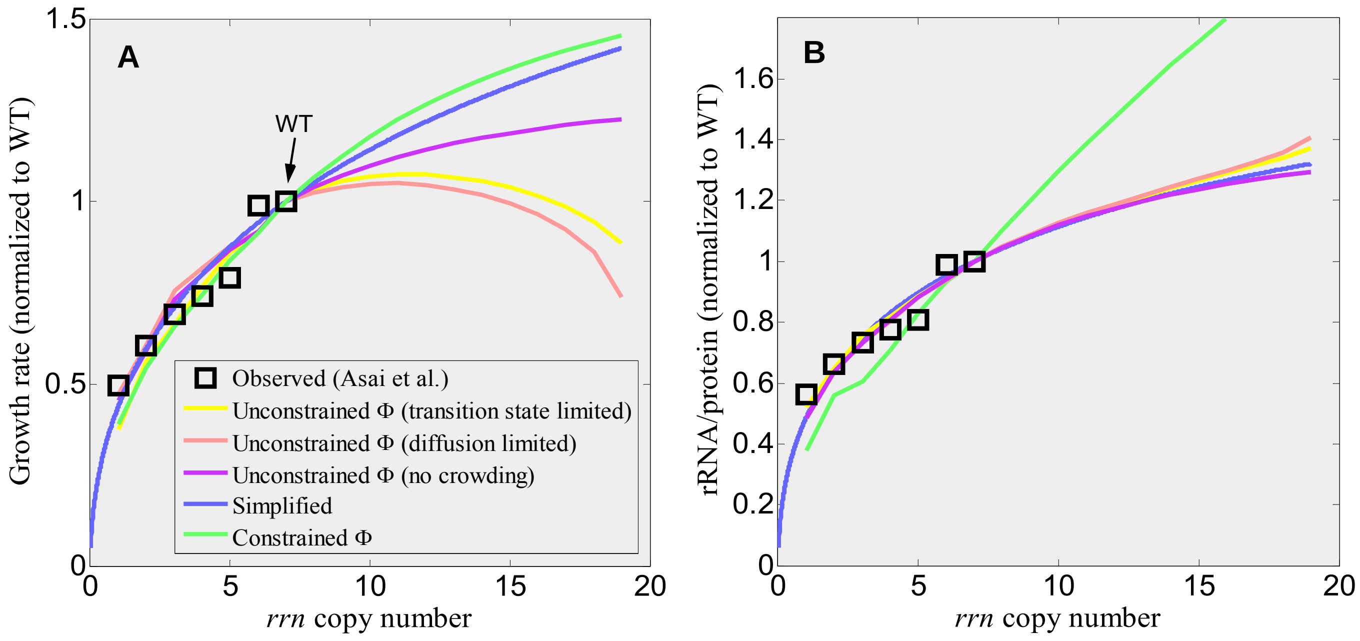

Figure S2. Fit for the constrained CGGR model with Hill coefficient *h*=1. Comparison of the constrained CGGR model with Hill coefficient *h*=1 to **(A)** growth rate measurements and **(B)** rRNA to total protein ratio measurements of Asai et al [9]. M_{bulk} was chosen such that the product of growth rate error and rRNA to total protein error was minimal, yielding $M_{bulk} = 5.7 \cdot 10^6$ molec/WT cell (c_p^{max} =45 aa/sec). For MSE see Fig. S1B. Note that for *h*=1, growth rate diverges with copy number. rRNA chain elongation rate, c_{rrn}, was assumed to be constant in this simulation.

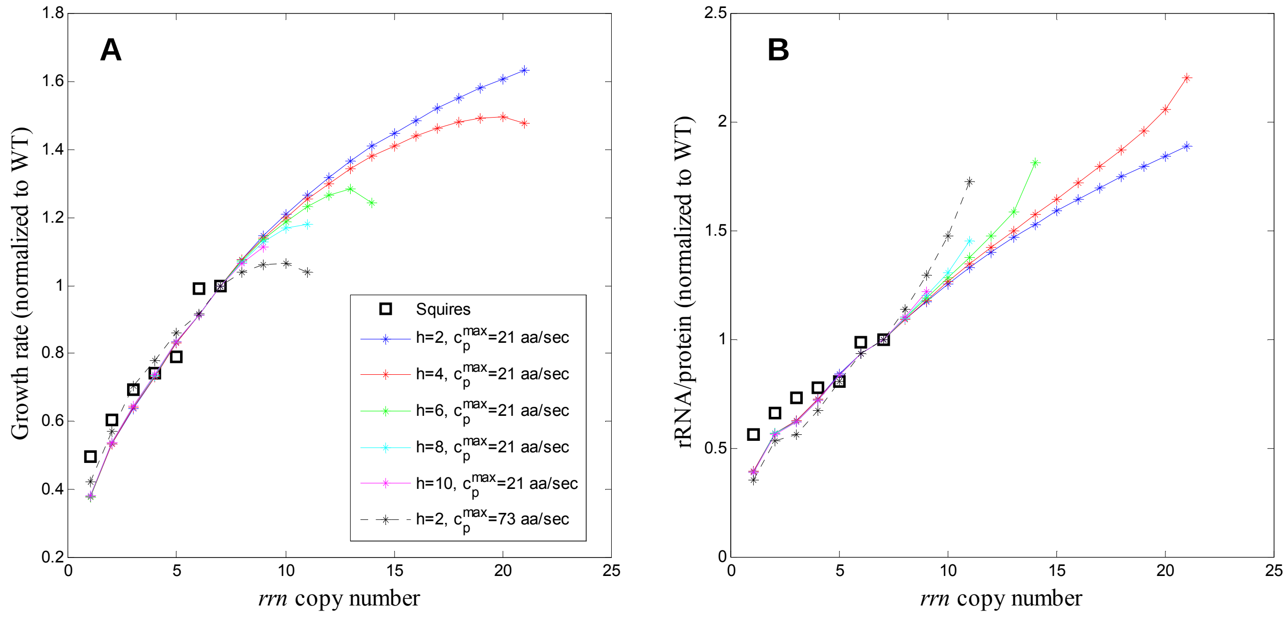

Figure S3. Fit for the constrained CGGR model with higher Hill coefficients. Comparison of the constrained CGGR model with Hill coefficients of 2, 4, 6, 8 and 10 to **(A)** growth rate measurements and **(B)** rRNA to total protein ratio measurements of Asai et al [9]. We show the *h*=2 case for both c_p^{max} =73 aa/sec (M_{bulk}=7.4·10^6 molec/WT cell; as in Fig. 2) and c_p^{max} =21 aa/sec. For all other cases, c_p^{max} was set to 21 aa/sec and corresponds to the minimum possible value for M_{bulk}., a value that according to Fig. S1B minimizes the MSE for the rRNA to total protein ratio. This figure demonstrates that all solutions with c_p^{max} =21 aa/sec diverge in growth rate for *rrn* copy numbers greater that 7. Higher Hill coefficients (>10) appear to be numerically unstable or insolvable for high copy numbers. Legend to both figures is given in (A).

S1.6 Free RNAp and free ribosome homeostasis

The fact that predictions of the unconstrained CGGR model are independent of the exact crowding scenario chosen is due to a self-adjusting mechanism where free RNAp and free ribosomes change in a way that tends to compensate for changes in the binding affinities. Figure S4A illustrates this point for transcription binding affinities in the transition state limited scenario (assumed to be optimal at the WT Φ). As the rRNA copy number is reduced, the macromolecular volume fraction, Φ, decreases, causing binding affinities to weaken. However, an increase in free RNAp compensates for this reduction. Since it is the *ratio* free RNAp with respect to the binding affinity that determines the transcription rate, the observed transcription rate is relatively unchanged, as can be seen by comparing to a solution where binding affinities were assumed to be independent of Φ. This conclusion gives a physical interpretation to the robustness of transcription related observables to changes in RNAp binding affinities noted by Bremer et al. [1]. The degree to which free RNAp can be increased is probably limited, and at some point it is expected that the negative transcriptional regulation circuit of *rpoBC* will kick in. A similar effect is observed for the diffusion limited model and for ribosome binding affinities as well (Fig. S4B-D).

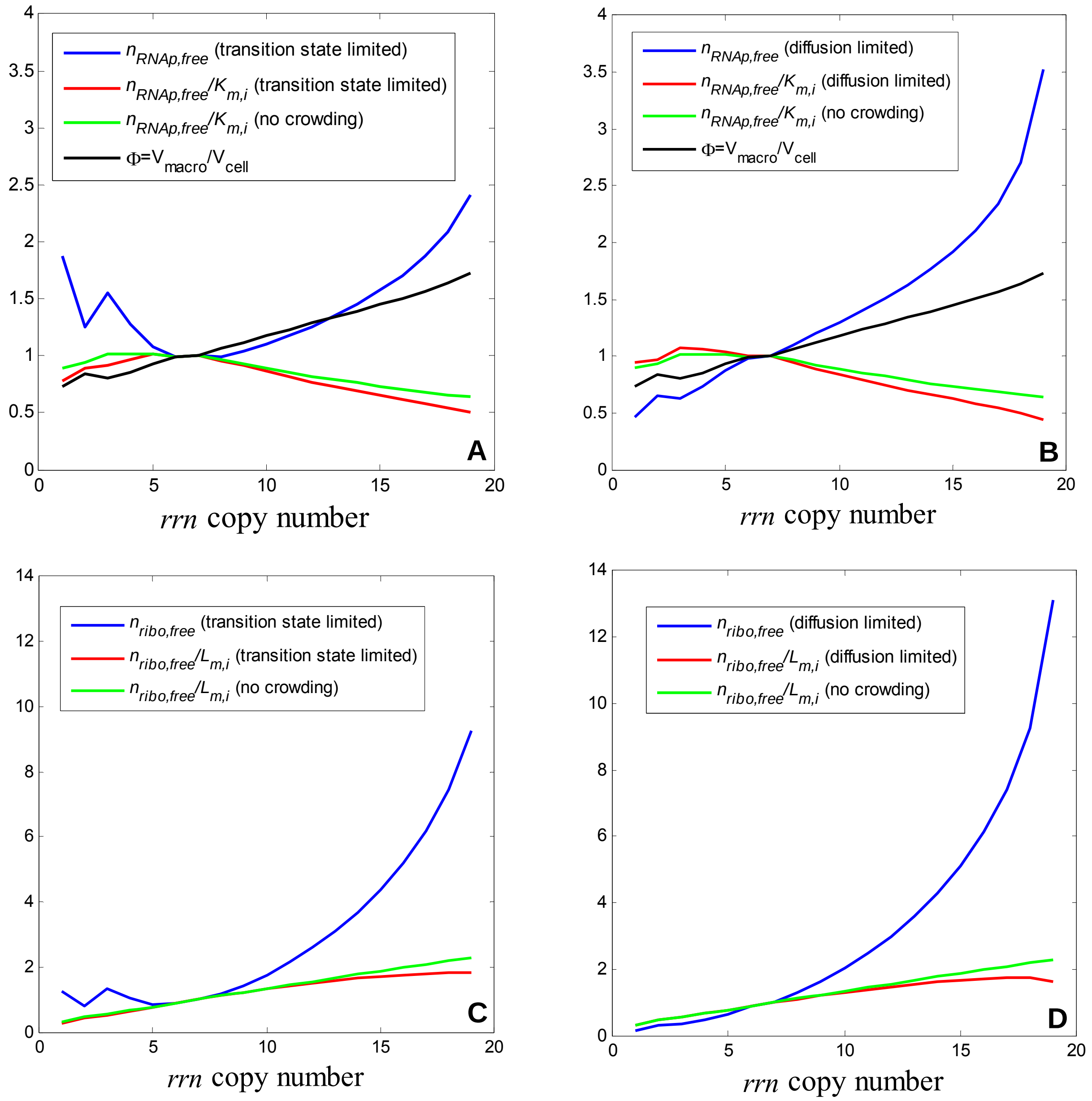

Figure S4. Free RNAp and free ribosomes with respect to corresponding binding affinities for various crowding scenarios. (A) Model prediction for $n_{RNAp,free}$, $n_{RNAp,\ free}/K_{m,i}$ and Φ for the transition state limited and no crowding scenarios as a function of the *rrn* operon copy number. In the no crowding scenario the plots for $n_{RNAp,free}$ and $n_{RNAp,\ free}/K_{m,i}$ coincide. **(B)** Same as (A) but for the diffusion limited scenario. **(C)** Model prediction for $n_{ribo,free}$ and $n_{ribo,free}/L_{m,i}$ for the transition state limited and no crowding scenarios as a function of the *rrn* operon copy number. **(D)** Same as (C) but for the diffusion limited scenario. All curves are normalized to WT values at copy number 7. Note that in the diffusion limited scenario, when rRNA operons are inactivated, free RNAp concentration actually decreases. The reasons for this are that first, although the rRNA operons are inactivated, they continue to be partly transcribed (c.f. S1.3). Second, as rRNA operons are inactivated, growth rate is reduced (Fig. 2A), which tends to slightly increase gene concentrations via Eq. 3 (c.f. Fig. S7B). Finally, there is the contribution of increased transcription initiation. When rRNA operons are increased beyond seven copies per chromosome, free RNAp concentration increases mainly because transcription initiation is reduced due to diminished binding affinities. See main text and S1.6 for further explanations.

S1.7 Further predictions

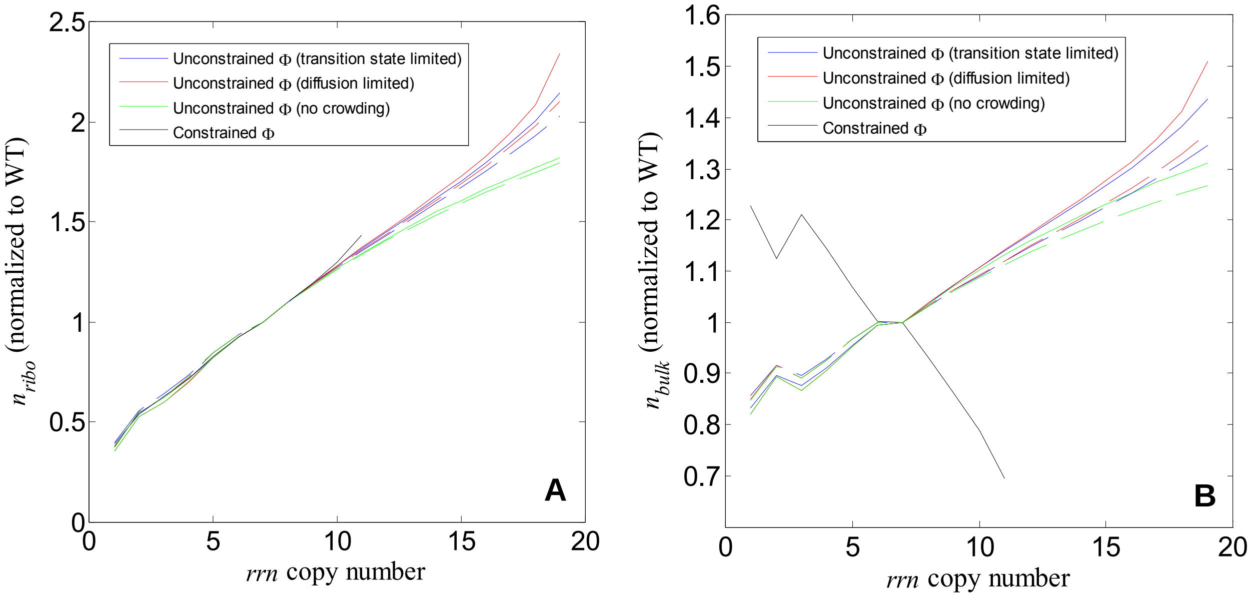

Figure S5. Predictions for bulk protein and ribosome concentrations as a function of the *rrn* operon copy number. (A) Total concentration of ribosomes (ribosomes per unit volume) in the constrained and unconstrained CGGR models as a function of the *rrn* operon copy number. **(B)** Concentration of bulk protein (proteins per unit volume) in the constrained and unconstrained CGGR models as a function of the *rrn* operon copy number. Solid lines are for fixed *rrn* chain elongation rate, c_{rrn}=const, and dashed lines are for c_{rrn} ≠const, as described in the main text. All curves are normalized to WT cell state values (at copy number = 7).

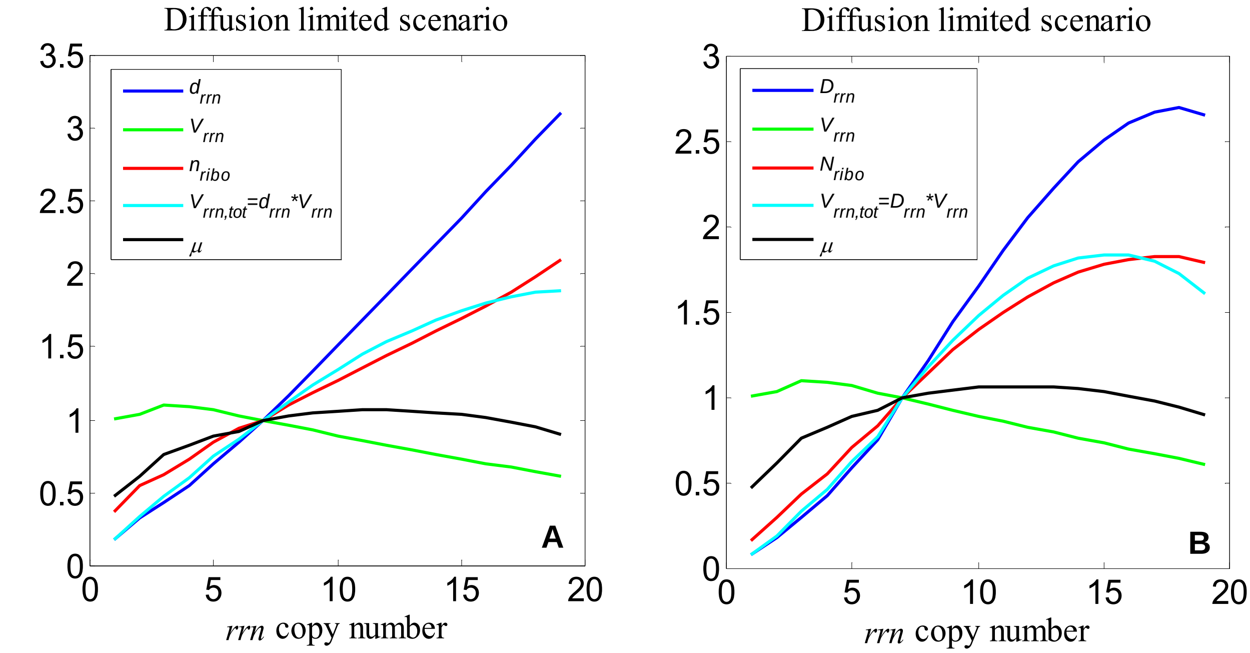

Figure S6. Breakdown of the ribosome synthesis equation to components for the diffusion limited scenario. (A) Variables in units of concentration. d_{rrn} - *rrn* gene concentration (total rRNA operon copy number per unit volume); V_{rrn} - *rrn* initiation rate per operon (init/min/operon); n_{ribo} - ribosome concentration (ribosomes per unit volume), and μ - growth rate. These parameters are tied by Eq. 2iii: $\alpha = d_{rrn} \cdot V_{rrn} / n_{ribo}$. **(B) Variables in units of molec/cell.** D_{rrn} - *rrn* gene dosage (total rRNA operon copy number per cell); N_{ribo} - number of ribosomes per cell. These parameters are tied by Eq. 2iii: $\alpha = D_{rrn} \cdot V_{rrn} / N_{ribo}$. This simulation is for the diffusion limited scenario assuming that the rRNA chain elongation rate, c_{rrn}, is variable, as described in the main text. All curves are normalized to WT cell state values (at copy number = 7).

S2. Model - supplementary information

S2.1 RNAp autoregulation

Functional RNAp (the holoenzyme) is composed of a core enzyme unit ($\beta\beta'\alpha_2$) associated with a σ subunit [11]. The amount of RNAp in the cell is limited by the amount of β and β' subunits, transcribed from the *rpoBC* operon [3,11]. Once the core enzyme is assembled, it is expected to be rapidly converted into holoenzyme due to excess of free σ over free core [12] (see also Table S2 footnote b).

The autoregulation of *rpoBC* is rather complex involving both free functional holoenzyme and core subunits [11], possibly operating on different timescales (see below). In the present model though, we assumed to a first order approximation an ideal negative autoregulation [35] that fixes the total concentration of RNAp at some growth rate independent value. In what follows we attempt to give the rational behind this assumption.

rpoBC is cotranscribed with genes for the ribosomal proteins L10 and L7/12 and is negatively autoregulated both at a transcriptional and at a translation level [3,11]. Although the details of these mechanisms are still being elucidated [1], the immerging picture [11] is that transcription is negatively autoregulated by free holoenzyme and translation is negatively autoregulated by the immature enzyme (β or β'), where the former may be regulated by ppGpp [3]. To be more specific, transcription of *rpoBC* is negatively autoregulated by upstream L10 and L12 promoters [11,36-38] apparently via the holoenzyme (and not a specific subunit) [11,37] and is possibly regulated by ppGpp [3]. In addition there is a read-through attenuator located upstream of *rpoBC* which also negatively autoregulates transcription of *rpoBC* via free RNAp [1,36-42], particularly during times of physiological stress [38]. There may also be a weak promoter just downstream of this attenuator [1,43]. Translation of *rpoBC* is also negatively autoregulated [11,38,39,44,45] by β or β' alone [11,38], presumably regulating excess levels of these subunits when these subunits are not incorporated into the core enzyme [11]. It has also been suggested that ppGpp may have some function in regulating the level of free RNAp in the cell [1].

As explained in the previous paragraph, the translation regulation mechanism regulates free β and β' subunits. Since these subunits are present in the pool of immature holoenzyme, it may be that the function of the translational regulation is to regulate the pool of immature holoenzyme, keeping it more or less at a fixed concentration. Since the pool of immature RNAp is proportional to the total

concentration of RNAp (cf. Eq. 2iv) (with a mild dependence on growth rate), it seems plausible that the translational regulation aims to keep the concentration of total RNAp constant. In principle, to determine the overall regulation of *rpoBC*, one would need to quantify the response function of both transcription and translation regulation levels and solve the dynamic coupling between the two. For the purpose of this model though we will assume that in a rich environment, the translation control mechanism is the dominate one and the concentration of total RNAp is therefore fixed.

This rather crude approximation can be partly supported by the following facts: In experiments that manipulate the level of the β and/or β' subunits the translational control mechanism response is stronger than the transcriptional control mechanism response [11,36,39] indicating that when the translational control circuit is insufficient to meet the requirements of β and β' subunits, transcriptional regulation may also be activated [39]. This can possibly be understood by noting that the translational loop can respond on a faster time scale than the transcriptional loop. Second, in a rich environment one expects ppGpp levels to be small and fixed [4], thus ppGpp should have little or no effect on the transcriptional regulation of upstream promoters. Finally, according to [1] free RNAp concentration changes more significantly than total RNAp concentration as a function of growth rate (albeit at different environments). Yet despite our assumption we keep in mind that significant changes in free RNAp concentration may also incur a response of the *rpoBC* transcriptional control mechanism and moderate these changes.

S2.2 Gene concentration

S2.2.1 Growth rate dependence of gene concentration

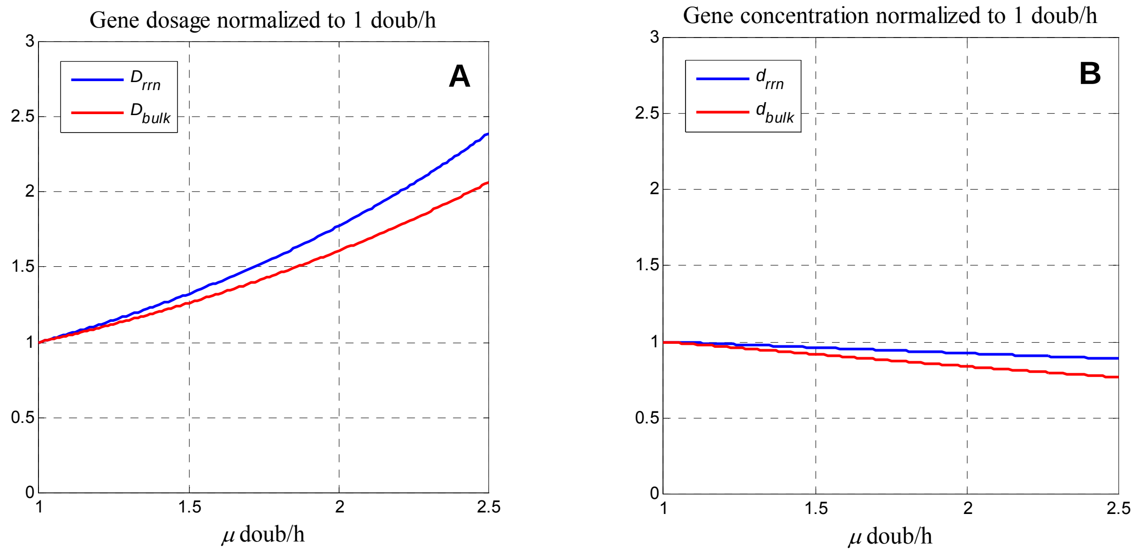

Figure S7. Gene dosage and gene concentration as a function of growth rate. (A) Gene dosage and **(B)** gene concentration for the *rrn* gene class and bulk gene class. C and D periods were interpolated based on data from table 2 of [3] as a second order polynomial in μ^{-1}. For this simulation we assumed that 66 evenly distributed bulk genes are expressed (c.f. map locations in Table S1). The initiation volume, V_{ini}, was assumed to be fixed [46-48]. See also main text and S2.2 for further explanations.

For reference, we quote the formula for the average number of genes per cell in an exponential culture (i.e. gene dosage) ([49], [3] table 5):

$$D_m(\mu) = 2^{\mu(C(1-m)+D)} \qquad \text{(S7)}$$

where C is the C period (the time required to replicate the chromosome, ~40 min), D is the D period (the time between termination of a round of replication and the following cell division, ~20 min) and m is the genetic map location ($0 \le m \le 1$). The formula for the average cell volume in an exponential culture is given by [50]:

$$V_{cell}(\mu) = V_{ini} 2^{\mu(C+D)} \ln 2 \qquad \text{(S8)}$$

Where V_{ini} is the initiation volume, defined as the ratio of the cell volume at the time of replication initiation and the number of origins per cell at that time. Eq. 3 is given by the ratio of these two expressions: $d_m(\mu) = D_m(\mu)/V_{cell}(\mu)$.

S2.2.2 The initiation volume

Experimental observations [3,47,48] and recent theoretical considerations relating to the replication initiation mechanism in *E. coli* [40,46] suggest that the initiation volume can be regarded as fixed and independent of genetic perturbations. We briefly consider the facts that support this conclusion. Experimentally, the initiation volume of *E. coli* is observed to be largely constant above a given growth rate (for example, for *E. coli* B/r this is true for μ above about 1 doub/h [47]). This appears to be intimately related to the replication initiation mechanism. Recent advances in the study of the replication initiation mechanism in *E. coli* have led to more detailed models of replication initiation ([40,46] and references quoted therein) that support the notion that the initiation volume should be fixed and largely growth rate independent [46]. Based on those models we showed that the initiation volume is inversely proportional to the total concentration of DnaA (the protein that regulates replication initiation) and other constants that at least to a first order approximation are growth rate independent. DnaA is negatively autoregulated ([46,51,52] and references quoted therein) and has been observed to be growth rate independent [53][12]. Although the mechanism of negative autoregulation is quite complex, for the present discussion we assume that it is ideal [35] and that its function is to simply keep the concentration of DnaA constant [46]. This suggests that the initiation volume is fixed presumably due to the negative autoregulation of DnaA and should be independent of genetic perturbations, unless these perturbations specifically target the replication initiation system. A more detailed discussion of these findings will be given elsewhere (Tadmor & Tlusty, in preparation).

S2.3 Genetic vs. environmental perturbations

The goal of the CGGR model is to calculate the cell state for genetically perturbed cells in a constant environment. Since these perturbations necessarily affect the growth rate, we need to clarify the how genetic parameters can be affected by growth rate. This clarification is especially important since in the literature the term "growth rate dependence" is often used in reference to the cell response to a change in medium composition (e.g. tables 2 and 3 in [3]). Thus we must distinguish between dependence arising from changes in the environment and a dependence arising from other factors.

The genetic parameters (defined in Table S1) can be coupled directly to the growth rate μ, coupled indirectly to μ or uncoupled. Promoter concentration (d_i) is coupled directly to μ due to the

[12] Specifically in *E. coli* B/r, DnaA concentration displays a slight growth rate dependence that is opposite to the growth rate dependence of the initiation mass [47,53]. This result is expected based on the inverse relation derived between the initiation volume and the concentration of DnaA.

replication initiation mechanism (Eq. 3). Indirect coupling can arise in response to genetic perturbations or environmental perturbations. The origin of the former coupling can be due, for example, to possible crowding effects (e.g. in the unconstrained CGGR model) which may affect for example binding affinities $K_{m,i}$, $L_{m,i}$ (e.g. Eq. S11 or S12) and in the relatively weak translation-degradation coupling modulating functional mRNA half-life, $T_{1/2,i}^{fun}$ (c.f. Eq. S15). Environmental coupling is not relevant for the situation we are considering because the environment is considered to be fixed (see below). For a discussion on how genetic parameters and constants can possibly be affected by perturbation of the *rrn* operon copy number refer to S1.3.

To see how environmental coupling could possibly affect the genetic parameters, we consider the case of cells in exponential growth at 37°C and we analyze possible effects of changing the nutrient composition on genetic parameters. Possible effects can include the following: (i) The number of bulk genes expressed will change. For example, cells growing in an amino acid poor medium will "switch on" genetic networks responsible for synthesis of amino acids, vitamins, cofactors, stress related proteins etc. [54] thus *increasing* the number of bulk genes being expressed [1,54]. Another aspect of this coupling is that the genetic parameters of the bulk protein gene class are essentially ensemble averages of the many different mRNAs being expressed. As the growth medium changes, the expression pattern of bulk genes changes, and expectation values may drift. In addition, gene expression of certain genes may be affected by changes in the nutrient composition of the environment (e.g. the *cat* gene [32]) and therefore the Michaelis-Menten parameters and half-life of individual genes may shift, thereby affecting the ensemble averages. (ii) mRNA half-life may be influenced by the growth medium, if, for example, the growth medium has an effect on the frequency of ribosome loading [55]. This is a result of the translation-degradation coupling [28] (iii) The *rrn* promoter equilibrium constant K_m is thought to be subject to regulation via ppGpp [8]. It has been suggested that the objective of this regulation is to keep c_p at its maximal value [8], although other theories postulate that *in vivo*, c_p is below its maximal value [56]. (iv) The peptide chain elongation rate c_p exhibits a certain growth rate dependence (see Table S1/table 3 in [3]). (v) mRNA chain elongation also exhibits a certain growth rate dependence [1,3]. mRNA transcription is affected by pausing of RNAp and is thought at least in part to be ppGpp dependent [1]. (vi) The level of RNAp may also be modulated by ppGpp [1,3,8].

S2.4 Crowding

S2.4.1 Overview of crowding effects

Crowding may have many consequences on biological processes *in vivo* such as increasing/decreasing reaction rates [57-62], subdiffuison [63,64], effects on protein folding and protein assembly [65-67], aggregation stimulation [67,68], non-steric interactions and the response of the cell osmotic changes [69-72]. We give a brief review of how crowding can affect a simple association reaction.

<u>Crowding effects on association reactions:</u>

It is expected that in the limit of high Φ all association reactions (such as holoenzyme-promoter association or 30S-RBS association) become diffusion limited [60]. In the limit of low Φ, the forward reaction rates of transition state limited association reactions are expected to increase due to volume exclusion effects or decrease if the reaction is diffusion limited [60]. Thus, the overall forward reaction rate for transition state limited reactions is expected to exhibit a maximum [57-61]. Yet the possibility may also exist that increased crowding (due to, e.g., genetic perturbations) will increase the activity of free ions such as K^+, which tend to weaken binding affinities [69-73] and possibly even invoke an osmotic response. Nevertheless, it has been proposed that the overall forward rate k_f for the basic association reaction $A+B \rightleftarrows AB^* \rightarrow C$ is approximated by [57-61]:

$$\left(k_f\right)^{-1} \simeq \left(k_{ts}^0 \Gamma\right)^{-1} + \left(k_{enc}^0 e^{-g\Phi}\right)^{-1} \qquad \text{(S9)}$$

where k_{ts}^0, k_{enc}^0 and g are constants, Γ is the nonideality factor given by $\Gamma = \gamma_A \gamma_B / \gamma_{AB^*}$, γ_i is the activity coefficient of component i, and AB^* is the transition state complex. The first term in Eq. S9 has a thermodynamic origin that reflects how entropy is affected by crowding and is appropriate when the reaction is transition state limited. For steric interaction γ_i>1 [74] and specifically for DNA-protein type interactions (where B would be the DNA) we expect that $\Gamma \approx \gamma_A > 1$ [10,69]. Note that the activity coefficient increases non-linearly with the radius of the tracer molecule due to an increase in excluded volume (and therefore a decrease in available volume)[13]: $\gamma_i \equiv V_{tot} / V_{available,i}$ where V_{tot} denotes total volume, and $V_{available,i}$ denotes the volume available for species i [60]. The second term has a hydrodynamic origin that reflects the decrease of the diffusion constant with increased crowding, and is appropriate for a diffusion limited type reaction [57]. Since all reactions become

[13] Since the 30S subunit is larger than the holoenzyme (840 kD vs. 435 kD) it is expected that the nonideality factor associated with it will be greater (see Fig. S8A).

diffusion limited at a high enough Φ [60], reactions that are transition state limited at low Φ are expected to exhibit a maximum in k_f[14]. If the dissociation constant is not greatly affected by crowding [74] then the equilibrium constant (in units of concentration) should exhibit a minimum.

Experimental reports on the effects of crowding are ample. The equilibrium constant of reactions has been observed to increase by up to 30 fold under crowded conditions and the forward rates of some protein-DNA interactions have also been observed to be greatly increased [62,75-77]. It has also been proposed that the equilibrium constant for the LacI-operator and RNAp-promoter interaction is increased due to crowding, rendering such reactions robust to environmental perturbations [10,69]. Effects of crowding on diffusion have been observed *in vitro* [63,78,79] and were modeled as stretched exponentials. *In vivo* diffusion coefficients inside the cytoplasm of *E. coli* appear to be greatly reduced compared to *in vitro* diffusion in non-crowded solutions [80]. In addition, a bimodal distribution exhibiting a maximum has also been observed *in vitro* for the forward rates [60,76].

Although many research efforts have been devoted to the subject of crowding (see e.g. [57,59,74,81-83] and recent reviews: [60,84,85]), prediction of crowding effects on biochemical processes *in vivo* remains challenging and implications are still being elucidated. For this reason we considered two different crowding scenarios:

(1) The transition state limited scenario, where it is assumed that holoenzyme-promoter and 30S-RBS binding affinities have been evolutionary tuned to be maximal at the macromolecular volume fraction of WT *E. coli* growing at 2 doub/h. The WT macromolecular volume fraction at this growth rate was measured to be Φ=0.34 [10]. In the limit of high Φ, binding affinities were assumed to decay exponentially with Φ [57,59,79] in accordance with Eq. S9.

(2) The diffusion limited scenario, where it is assumed that all binding affinities decay exponentially.

(3) For reference we also considered the (nonphysical) "no crowding" scenario: all binding affinities were assumed to be independent of Φ.

[14] de Smit et al. [13] proposed that *in vitro* 30S-mRNA association may be diffusion rate limited since *in vitro* measured on rates are of the order of the diffusion limit. Yet the diffusion limit may actually be increased if the effective target size for the 30S subunit increases substantially as a result of 30S sliding on the DNA in the process of target (RBS) location, an effect suggested in [13].

For (1) and (2), holoenzyme and 30S activities were calculated using an approximate statistical mechanics model of a hard sphere fluid mixture (water molecules + background crowders) based on Scaled Particle Theory (SPT) implementing Berg's method [82,83] (discussed next).

Fig. S8 shows the dependence of binding affinities on the volume fraction Φ for the transition state limited crowding scenario (Fig. S8A) and for the diffusion limited crowding scenario (Fig. S8B).

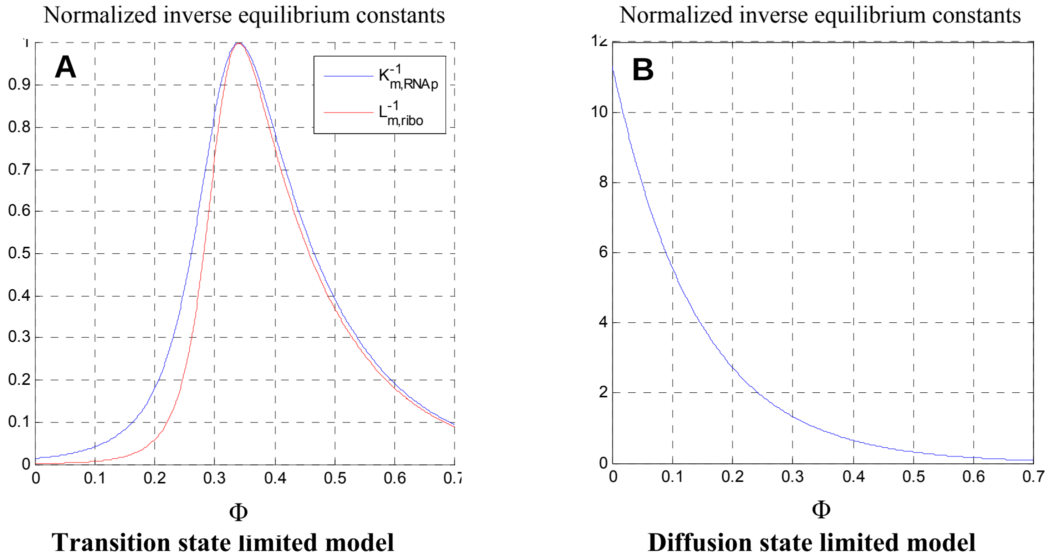

Figure S8. Dependence of binding affinities on the volume fraction Φ for the various crowding scenarios. **(A)** Normalized inverse equilibrium constants, K_m^{-1} and L_m^{-1} (in units of 1/M), for the RNAp holoenzyme (radius 5.57nm) and the 30S ribosome subunit (radius 6.92 nm) respectively, in the transition state limited model. The water molecule radius was taken to be 0.138 nm [82] and the radius of the background crowding agent was taken to be 3.06 nm [10]. **(B)** Normalized K_m^{-1} and L_m^{-1} for the diffusion limited model (curves overlap). All curves were normalized to values at the WT volume fraction of Φ=0.34. See S2.4.3 for more details.

S2.4.2 Calculation of activity coefficients and binding affinities as a function of Φ

Minton, one of the pioneers of the field of crowding, suggested early on to use Scaled Particle Theory (SPT) to calculate activity coefficients for biologically related environments [59,74]. Minton's γ however diverge as $R_e \to 0$ (for fixed Φ), where R_e is the radius of the background crowding molecule. Berg suggested that in a hard-sphere description one would expect $\gamma \to 1$ when R_e approaches the size of the solvent molecules [82]. This contradiction is due to the fact in Minton's hard sphere description, the background water molecules and small cosolvents were treated as a continuum [85]. In a model proposed by Berg [82], the solvent was treated as a separate component in a hard-sphere mixture. Berg's description insures that the activity coefficient of a tracer $\gamma_{tracer}(R_e{=}R_{water}){=}1$ for any R_{tracer}. This remedied the divergence problem and also predicted considerably lower values for γ (Fig. S9). Guttman et al. compared Berg's and Minton's approaches for calculating γ. Those authors concluded that Berg's description gives a better fit to experimental data, as Berg himself suggested [82,83]. Guttman et al. also proposed a more precise way of determining the volume occupation of water Φ_W than Berg's approximation – see below (Fig. S9 and S10).

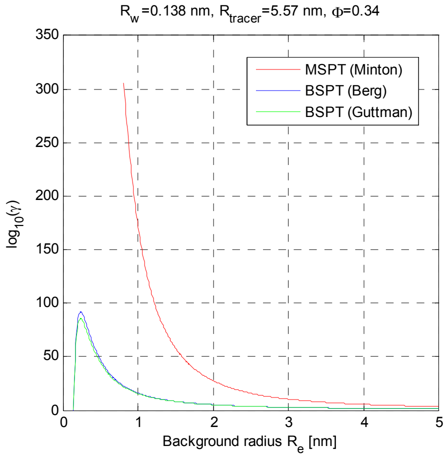

Figure S9. Dependance of activity coeficients on the crowder radius. SPT calculation of activity coeffiecients based on Minton (red), Berg (blue) and Guttmann et al. (green).

<u>Method of Berg [82]:</u>

Next we summarize the formulae used to calculate the activity coefficients via Berg and Guttman et al..

The activity coefficient for the *i*-th species is given by

$$\ln \gamma_i = g(a,b,\Phi) - g(a,b,0) \quad \text{(S10)}$$

where $g(a,b,\Phi)$ is the reversible work required to create a cavity of size R_i in a hard-sphere mixture (water molecules + background crowders).

$$g(a,b,\Phi)/k_BT \triangleq -\ln(1-S_3)+\frac{6S_2}{1-S_3}R_i+\left(\frac{12S_1}{1-S_3}+\frac{18S_2^2}{(1-S_3)^2}\right)R_i^2+\frac{4\pi}{3}R_i^3P$$

where $a \triangleq R_e/R_W$, $b \triangleq R_i/R_e$ and:

$S_1 \triangleq \frac{1}{4}\left(a^2\Phi_W+\Phi\right)R_e^{-2}$

$S_2 \triangleq \frac{1}{2}\left(a\Phi_W+\Phi\right)R_e^{-1}$

$S_3 \triangleq \Phi_W+\Phi$

R_W is the radius of a water molecule = 0.138 nm [82].

R_e is the radius of a background crowding agent = 3.06 nm [10].

R_i is the tracer molecule radius (5.57 nm for RNApσ or 6.92 nm for the 30S ribosome subunit– see estimates below).

Φ_W is the volume fraction of water molecules and is given (to first order in Φ) by

$\Phi_W(a,\Phi)=\Phi_W^0\left[1-\Phi-\Phi f(a)\right]$

$\Phi_W^0=\Phi_W(a,\Phi=0)=0.363$ is the volume occupancy of the solvent molecules in pure solvent [82].

$$f(a)\triangleq\frac{3\left(1-\Phi_W^0\right)}{\left[a(1+2\Phi_W^0)\right]^2}\left\{a\left(1+2\Phi_W^0\right)+(1-\Phi_W^0)\left[1+\frac{1-\Phi_W^0}{3a\Phi_W^0}\right]\right\}$$

The pressure term in g (fourth term) cancels in the subtraction in Eq. S10 since it is assumed that $P\left(\Phi_W,\Phi\right)=P\left(\Phi_W^0,0\right)$ (the pressure is assumed to be constant).

Method of Guttman et al. [83]

Guttman et al. suggested that an exact solution for Φ_W can be obtained by solving for Φ_W directly:

$$P\left(\Phi,\Phi_W\right)\triangleq\frac{6}{\pi}\left(\frac{S_0}{1-S_3}+\frac{3S_1S_2}{(1-S_3)^2}+\frac{3S_2^3}{(1-S_3)^3}\right)$$

where $S_0 \triangleq \frac{1}{8}\left(a^3\Phi_W+\Phi\right)R_e^{-3}$

and from the formula

$P\left(\Phi_W,\Phi\right)\overset{!}{=}P\left(\Phi_W^0,0\right)\rightarrow$ slove for Φ_W.

Since the equilibrium constants in the transition state limited and diffusion limited scenarios are dominated by the exponential decay at high Φ, the difference between the methods in estimating the equilibrium constants was negligible, and Berg's method is computationally less intensive.

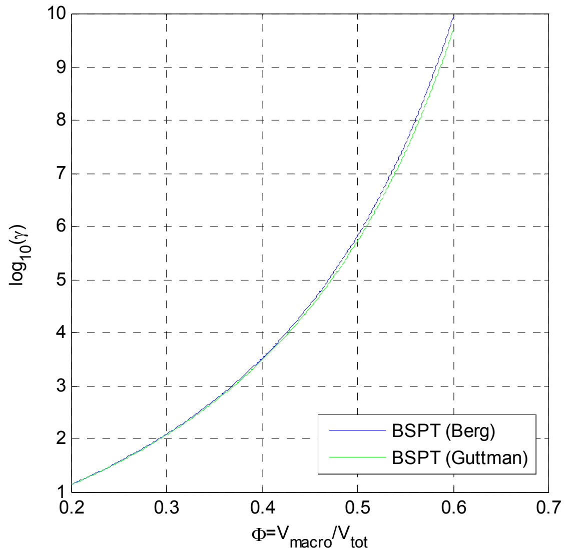

Figure S10. Dependence of activity coefficients on the volume fraction. Difference in γ as calculated by Berg [82] and Guttman et al. [83]. At Φ=0.45 the difference is only ca. 16% and at Φ=0.5 the difference is ca. 26%. R_w=0.138 nm, R_i=5.57 nm, R_e=3.06 nm. Berg also notes that his approximation works well up to $\Phi \cong 0.5$.

Finally we note that a more elaborate model of crowding than the one presented here may represent the background species not as spheres with a single effective radius, but as a combination of radii appropriate for the different gene class products (ribosomes, bulk proteins, load genes etc.). Such a background may prove to be a more dynamic and truer to reality background than the background used here.

S2.4.3 Crowding scenarios

S2.4.3.1 The transition state limited scenario

The binding affinities for this scenario were calculated based on the following model:

$$
\begin{aligned}
K_{m,i} &= \frac{1}{K^0_{chem,i}}\frac{1}{\gamma_{E\sigma^{70}}} + \frac{1}{K^0_{diff,i}} e^{g_{E\sigma 70}\Phi} \\
&\text{where} \quad \left.\frac{\partial K_{m,i}}{\partial \Phi}\right|_{\Phi=0.34} = 0, \quad K_{m,i}(\Phi = 0.34) = K^0_{m,i} \\
L_{m,i} &= \frac{1}{L^0_{chem,i}}\frac{1}{\gamma_{30S}} + \frac{1}{L^0_{diff,i}} e^{g_{30S}\Phi} \\
&\text{where} \quad \left.\frac{\partial L_{m,i}}{\partial \Phi}\right|_{\Phi=0.34} = 0, \quad L_{m,i}(\Phi = 0.34) = L^0_{m,i}
\end{aligned}
\qquad \text{(S11)}
$$

where $E\sigma^{70}$ is the RNAp holoenzyme (with a σ^{70} subunit)[15] and 30S is the 30S ribosome subunit. $K^0_{m,i}, L^0_{m,i}$ are the estimated WT *in vivo* binding affinities (Table S5). The two conditions following the definition of $K_{m,i}$ and $L_{m,i}$ are used to determine $K^0_{chem,i}, K^0_{diff,i}$ and $L^0_{chem,i}, L^0_{diff,i}$ respectively. We assumed that $g_{E\sigma} = g_{70S} = 7.13$[16]. $\gamma_{E\sigma}, \gamma_{30S}$ were calculated according to Eq. S10 and the method of Berg for $R_{E\sigma}$=5.57 nm and R_{30S}=6.92 nm[17]. Φ for WT *E. coli* at 2 doub/h has been estimated to be 0.34, assuming the specific macromolecule volume of v=1 g/ml [10].

A plot of normalized K_m^{-1} and L_m^{-1} vs. Φ, is given in Fig. S8A, based on Eq. S9, assuming $K_m \sim k_f^{-1}$, $\Gamma \approx \gamma_A$ (A=holoenzyme/30S) and $g = 7.13$. We find that $K_m(0)/K_m(0.34)$=73 and $L_m(0)/L_m(0.34)$=683, which is plausible compared to experimental values. For example, for the nick translation reaction for *E. coli* via Pol I, K_{eq} was increased by a factor of 30 [75] and here the holoenzyme (435 kD) and 30S subunit (840 kD) are more massive than Pol I (103 kD).

---

15 This is the major form of holoenzyme during exponential growth [11]..

16 The decay coefficient g was calculated based on the observation that D/D_0 is independent of the tracer size [79] and the fact that for GFP *in vivo* D/D_0~7.7/87 =exp(-0.34g) [80], thus g~7.

17 $R_{E\sigma}$=5.57 nm and R_{30S}=6.92 nm were determined from the molar masses of the macromolecules and assuming a specific volume of v=1 g/ml [10], as described in [81]. The masses are given by:

$M_{E\sigma} = \frac{4}{3}\pi R^3_{E\sigma} v = \frac{1}{3}(108 \text{ Dalton/aa})(\beta\beta' = 8258 \text{ bp} + \alpha_2 = 2*990 \text{ bp} + \sigma_{70} = 1839 \text{ bp}) = 0.435 \text{ MDalton}$ and

$M_{30S} = \frac{4}{3}\pi R^3_{30S} v = \text{30S r-protein+16S rRNA} = \frac{1}{3}(108 \text{ Dalton/aa})(9327 \text{ bp}) + (324 \text{ Dalton/RNA nuc})(1542 \text{ nuc}) = 0.835 \text{ MDalton}$ [6].

S2.4.3.2 The diffusion limited scenario

The binding affinities for this scenario were calculated based on the following model:

$$\begin{aligned} K_{m,i} &= \frac{1}{K^0_{diff,i}} e^{g_{E\sigma 70}\Phi} \quad \text{where} \quad K_{m,i}(\Phi = 0.34) = K^0_{m,i} \\ L_{m,i} &= \frac{1}{L^0_{diff,i}} e^{g_{30S}\Phi} \quad \text{where} \quad L_{m,i}(\Phi = 0.34) = L^0_{m,i} \end{aligned} \tag{S12}$$

Assuming as in Eq. S11 that $g_{E\sigma} = g_{70S} = 7.13$. See Fig. S8B.

In the simulations presented in main text we assumed the same crowding scenario for all gene classes. We note that for the transition state limited model this assumption is problematic for the *cat* protein since it is not native to *E. coli* and thus has not been optimized by evolution for the WT *E. coli* cytoplasm.

S2.5 Derivations

S2.5.1 Approximation of immature pool sizes

Both RNAp holoenzyme and ribosomes are multi-complex structures that have finite assembly times (note the delay boxes in Fig. 1A). In the case of ribosomes, there is also the time required for rRNA and tRNA maturation. Therefore a finite fraction of the total units that is synthesized is sequestered in pools of immature complexes. We have estimated this fraction using time delay equations. Assuming that the synthesis rate of the limiting constituents of these complexes (β/β’ for RNAp and rRNA for ribosomes) grows approximately exponentially with time (see below), then the fraction of immature complexes is given by (see derivation below):

$$\gamma \triangleq \frac{N_{imm}(t)}{N_{tot}(t)} = \frac{N_{imm}(t)}{N_{imm}(t)+N_{func}(t)} = 1-e^{-\alpha\tau} \tag{S13}$$

where $N_{imm}(t)$ is the number of immature molecules per cell, $N_{func}(t)$ is the number of assembled and functional complexes per cell and τ is the assembly time. Thus, when $\tau \to 0$ then $N_{imm} \to 0$ (i.e. assembly is instantaneous, pools do not accumulate) and when $\tau \to \infty$ (the product is never assembled) $N_{imm} \to N_{func}$ (i.e. entire product is always in the immature state). Note that γ is time independent. The assembly time for RNAp and ribosomes is approximately 3 min [1] and 5 min [3] respectively. Therefore at 2.5 doub/h, ~8% and ~13% of total RNAp and ribosome respectively are sequestered in immature pools. It has been suggested in [1] that $\gamma = e^{\alpha\tau}-1$. In the limit $\tau \ll T_{cycle}$ (T_{cycle}=log2/α) both solutions agree to first order: $\gamma = \alpha\tau$, but for $\tau \to \infty$, the solution of [1] diverges which physically is nonsensical.

Derivation

Given that it takes on average τ minutes to assemble a multi-complex, designated as X, we wish to find the percentage of X that is immature.

Let $N_{imm}(t)$ be the number of immature X per cell, $N_{func}(t)$ be the number of functional units of X and let $b(t)$ be the synthesis rate (molecules per min) of the limiting constituent in the assembly of X (for RNAp holoenzyme this would be the β and β’ subunits and for ribosomes this would be rRNA).

The size of immature pool is given by the following delay equations:

$$\begin{aligned} &(i) \quad \dot{N}_{imm} = b(t) - b(t-\tau) \\ &(ii) \quad \dot{N}_{func} = b(t-\tau) \end{aligned} \tag{S14}$$

Eq. S14-i implies that the limiting constituent of X (e.g. β and β') leave the pool after τ minutes at the rate at which they were formed and become products (X) in Eq. S14-ii. The immature pool of X is replenished at the current rate of synthesis of the limiting constituent of X: *b(t)*. At steady state exponential growth, we shall assume that the synthesis rate *b(t)* is given by $b(t) = b_o e^{\alpha t}$ where α= μ log(2) and μ is the doubling time. For β and β' this approximation is consistent with our approximation that the total concentration of RNAp (and hence β and β' subunits) is constant, thus β and β' must accumulate exponentially in the cell. For rRNA, *rrn* genes are scattered at various locations in the chromosome (albeit not uniformly) therefore gene dosage increases steadily with time, which we approximate as an exponential increase. Since the concentration of free RNAp is also relatively constant in time (due to periodic boundary conditions) this implies that synthesis rate of rRNA (=$D_{rrn}V_{rrn}$) can be roughly approximated to be exponential in time.

Imposing the boundary conditions

$$N_{imm}(t = T_{cycle} \equiv \mu^{-1}) = 2N_{imm}(0)$$
$$N_{func}(t = T_{cycle} \equiv \mu^{-1}) = 2N_{func}(0)$$

we find that

$$N_{imm}(t) = \frac{b_0}{\alpha}\left(1 - e^{-\alpha\tau}\right)e^{\alpha t}$$
$$N_{func}(t) = \frac{b_0}{\alpha}e^{\alpha(t-\tau)}$$

Therefore the percent of immature X is simply

$$\gamma = \frac{N_{imm}(t)}{N_{imm}(t) + N_{func}(t)} = 1 - e^{-\alpha\tau}$$

S2.5.2 The steady state rate equations

Table S8 summarizes the notation used in the reaction equations (R1)-(R3) and in the following derivation.

Index (i)	Gene class
1	Bulk
2	r-protein
3	sRNA = rRNA + tRNA
4	RNAp

Table S8. Notation used for derivations. Definition of notation used for reaction equations (R1), (R2) and (R3) and for the kinetic rate equations derived below.

Reaction equations	
P	free functional RNAp (holoenzyme)
R	free functional ribosome 30S subunit/ functional ribosome
$RNase$	free RNase E
$Protease$	free Protease
M_i∘$RNase$	RNase E bound to 5' end of i-th gene class mRNA
d_i	i-th promoter
Pd_i	RNAp-promoter complex for i-th gene class
$P\mathbf{M}_i$	RNAp-nascent mRNA(=$\mathbf{M}_i$) complex for i-th gene class
M_i	ribosome binding site (RBS) for i-th gene class mRNA
RM_i	30S-RBS complex for mRNA of i-th gene class
$R\mathbf{X}_i$	ribosome-nascent peptide chain(=$\mathbf{X}_i$) complex for product of i-th gene class
X_i	protein of i-th gene class<br>X_1 = B (bulk protein); X_2 = r-protein; X_4 = RNAp
Genetic Parameters and associated variables	
$V^{max}{}_i$	maximum rate of transcription initiation of the i-th gene class [8]
$U^{max}{}_i$	maximum rate of translation initiation of the i-th gene class mRNA [13]
$W^{max}{}_i$	maximum rate of degradation of the i-th gene class mRNA once RNase E is bound to the 5' end of the mRNA
$t_{c,i}$	average time to transcribe gene class i (=L_i/c_i), where c_i is the RNA chain elongation rate of the i-th gene class
$t_{l,i}$	average time to translate gene class i (=$L_i/3c_p$), where c_p is the peptide chain elongation rate
$K_{m,i}$	RNAp(holoenzyme)-promoter equilibrium constant
$L_{m,i}$	30S-RBS equilibrium constant
$J_{m,i}$	RNase E – 5' end of i-th gene class mRNA equilibrium constant
J_1	RNase E – 5' end of RNase E mRNA equilibrium constant
$\tau_{fun,i}$	functional lifetime of mRNA ($T^{fun}_{1/2,i} = \tau_{fun,i} \ln 2$)
$\tau_{chem,i}$	chemical lifetime of mRNA ($T^{chem}_{1/2,i} = \tau_{chem,i} \ln 2$)
γ_i	degradation rate of protein i
d_i	gene concentration of i-th gene class
$m_j^{(i)}$	map location of the j-th gene in i-th gene class
State variables	
[P], $n_{RNAp,free}$	concentration of free functional RNAp
[R], $n_{ribo,free}$	concentration of free functional ribosome 30S subunits
P^{tot}, n_{RNAp}	concentration of total RNAp
R^{tot}, n_{ribo}	concentration of total ribosomes
[B]	concentration of bulk protein
1/μ	doubling time of cell, α=μlog(2)=cell dilution rate
Other parameters/variables	
c_{ribo}	production cost of one ribosome (in units of # of bulk proteins)
n_0	minimum cell density
C	C period
V_{ini}	initiation volume
v_i	Volume of species i
Φ	macromolecule volume fraction = $V_{macromolecule}/V_{cell}$

Reaction equations

The three basic reaction taking place in the cell are: (R1) stable RNA (ribosome) synthesis, (R2) bulk protein synthesis, (R3) mRNA degradation (see also Fig. 1). Superscript symbols represent incoming reactants and subscript symbols represent outgoing products.

(R1) sRNA (ribosome) synthesis

$$\underbrace{[P]+[d_{rrn}] \overset{K_{m,rrn}}{\rightleftarrows} [Pd_{rrn}] \xrightarrow{V_{rrn}^{\max}} [P\mathbf{M}_{rrn}]_{+[d_{rrn}]}}_{\text{transcription initiation}} \underbrace{\xrightarrow{1/t_{c,rrn}} [R]_{+[P]}}_{\text{rRNA+tRNA elongation}}$$

(R2) Bulk protein synthesis

$$\underbrace{[P]+[d_{bulk}] \overset{K_{m,bulk}}{\rightleftarrows} [Pd_{bulk}] \xrightarrow{V_{bulk}^{\max}} [P\mathbf{M}_{\mathbf{bulk}}]_{+[d_{bulk}]}}_{\text{transcription initiation}} \underbrace{\xrightarrow{1/t_{c,bulk}} [M_{bulk}]_{+[P]}}_{\text{mRNA elongation}} \Rightarrow$$

$$\underbrace{[M_{bulk}]^{+[R]} \overset{L_{m,bulk}}{\rightleftarrows} [RM_{bulk}] \xrightarrow{U_i^{\max}} [R\mathbf{B}]_{+[M_{bulk}]}}_{\text{translation initaiton}} \underbrace{\xrightarrow{1/t_{l,bulk}} [R]+[B]}_{\text{peptide chain elongation}} \underbrace{{}^{+[\text{Protease}]} \xrightarrow{\gamma_{bulk}} [\text{protease}]+\text{decay fragments}}_{\text{protein degradtion}}$$

(R3) mRNA decay via the 5' competition model

$$[M_{bulk}]+[\text{RNase E}] \overset{J_{m,bulk}}{\rightleftarrows} [M_{bulk}\text{RNase E}] \xrightarrow{W_{bulk}^{\max}} [\text{RNase E}] + \text{decay fragments}$$

Notation is as follows: P is the free functional RNAp holoenzyme, R is the free functional 30S ribosome subunit or functional ribosome[18], B is the bulk protein. d_i and M_i represent the free promoters and free RBSs respectively (and also the final rRNA or mRNA product) belonging to the i-th gene class. $\boldsymbol{PM_i}$ denotes the transcribing complex of gene class i, $\boldsymbol{RB}$ denotes the translating complex for bulk protein. All symbols represent concentrations.

Conservation equations:

(B.1) $P^{tot}=[P]+\sum_{i=1}^{4}[Pd_i]+\sum_{i=1}^{4}[P\mathbf{M}_i]=[P]+\sum_{i=1}^{4}(1+t_{c,i}V_i^{\max})[Pd_i]\simeq[P]+\sum_{i=1}^{4}t_{c,i}V_i^{\max}[Pd_i]$ (total RNAp concentration)

(B.2) $R^{tot}=[R]+\sum_{i=1}^{3}[RM_i]+\sum_{i=1}^{3}[R\mathbf{X}_i]=[R]+\sum_{i=1}^{3}(1+t_{l,i}U_i^{\max})[RM_i]\simeq[R]+\sum_{i=1}^{3}t_{l,i}U_i^{\max}[RM_i]$ (total ribosome concentration)

(B.3) $d_i^{tot}=[d_i]+[Pd_i]$ (total promoter concentration)

(B.4) $M_i^{tot}=[M_i]+[M_i\circ\text{RNase}]+[RM_i]=[M_i\varnothing]+[RM_i]$ (total mRNA concentration)
$\varnothing\equiv$ with or without RNase E

[18] In this notation there is no distinction between a 30S subunit and a functional ribosome. This is because functional ribosomes are assumed to dissociate *in vivo* into the 30S and 50S subunits due to high concentrations of IF3 that prevents 30S-50S association (see Table S2 footnote d). Thus [R] is both the free 30S subunit and the product of rRNA + r-protein assembly.

Where in (B.1) and (B.2) we neglect RNAp and ribosome 30S subunit bound to the promoter (this can be included by redefining c_i and c_p - see footnotes k and l in Table S1).

Rate equations:

(B.5) $\frac{d[P\mathbf{M}_i]}{dt} = V_i^{\max}[Pd_i] - \left(\frac{1}{t_{c,i}} + \alpha\right)[P\mathbf{M}_i]$

(B.6) $\frac{d[Pd_i]}{dt} = k_i[P][d_i] - (k_{-i} + V_i^{\max} + \alpha)[Pd_i]$

(B.7) $\frac{d[R\mathbf{X}_i]}{dt} = U_i^{\max}[RM_i] - \left(\frac{1}{t_{l,i}} + \alpha\right)[R\mathbf{X}_i]$

(B.8) $\frac{d[X_i]}{dt} = \frac{1}{t_{l,i}}[R\mathbf{X}_\mathbf{i}] - (\alpha + \gamma_i)[X_i]$

$$\frac{d[M_i]}{dt} = \frac{1}{t_{c,i}}[P\mathbf{M}_i] - \alpha[M_i] + \left(U_i^{\max} + l_{-i}\right)[RM_i] - l_i[M_i][R] - j_1[M_i][\text{RNase}] + j_{-1}[M_i \circ \text{RNase}]$$

(B.9) $\frac{d[M_i \circ \text{RNase}]}{dt} = j_1[M_i][\text{RNase}] - \left(W_i^{\max} + j_{-1}\right)[M_i \circ \text{RNase}] - \alpha[M_i \circ \text{RNase}]$

(B.10) $\frac{d[M_i\varnothing]}{dt} \equiv \frac{d[M_i]}{dt} + \frac{d[M_i \circ \text{RNase}]}{dt} = \frac{1}{t_{c,i}}[P\mathbf{M}_i] - \alpha[M_i\varnothing] + \left(U_i^{\max} + l_{-i}\right)[RM_i] - l_i[M_i][R] - W_i^{\max}[M_i \circ \text{RNase}]$

(B.11) $\frac{d[RM_i]}{dt} = l_i[R][M_i] - (l_{-i} + U_i^{\max} + \alpha)[RM_i]$

(B.12) $\frac{dM_i^{tot}}{dt} = \frac{d[M_i\varnothing]}{dt} + \frac{d[RM_i]}{dt} = \frac{1}{t_{c,i}}[P\mathbf{M}_i] - \alpha M_i^{tot} - W_i^{\max}[M_i \circ \text{RNase}]$

(B.13) $\frac{d[R]}{dt} = \frac{1}{t_{c,rrn}}[P\mathbf{M}_{rrn}] + \sum_{i=1,2,4}\left[\frac{1}{t_{l,i}}[R\mathbf{X}_i] + l_{-i}[RL_i] - l_i[M_i][R]\right] - \alpha[R]$

We will assume that no appreciable protein degradation occurs on a cell cycle time scale, i.e. $\alpha_i >> \gamma_i$ [66] in (B.8).

Adiabatic approximations

From (B.6):

$$\frac{d[Pd_i]}{dt} = k_i[P][d_i] - \Gamma[Pd_i]$$
$$\Gamma = k_{-i} + V_i^{\max} + \alpha$$

Assuming $[d_i],[P]$ change on a timescale $>>1/\Gamma$ (we assume they change on a cell cycle timescale), we can solve this equation under the adiabatic approximation:

$$[Pd_i]=\frac{1}{\Gamma}k_i[P][d_i](1-e^{-\Gamma t})$$

and for timescales > 1/Γ

$$[Pd_i]\simeq\frac{1}{\Gamma}k_i[P][d_i]$$

where 1/Γ is governed by the fastest rate. To estimate Γ we note that $K_{m,bulk}$=0.7 μM [1], $V^{max}{}_{bulk}$= 3 ini/min [1] and $k_{-1,\ bulk}$ is set by the diffusion limit: $k_{1,bulk}$~10^9 (M sec)$^{-1}$ [86] then $1/\Gamma \sim 1/k_{-1,\mathrm{bulk}} \sim \mathrm{msec}$.

Therefore

(B.14) $[Pd_i]^{SS}\simeq\frac{1}{K_{m,i}}[P][d_i]$

Where $K_{m,i}\equiv\left(k_{-i}+V_i^{\max}+\alpha\right)/k_i\cong\left(k_{-i}+V_i^{\max}\right)/k_i$

Next

$$\frac{d[RM_i]}{dt}=l_i[R][M_i]-(l_{-i}+U_i^{\max}+\alpha)[RM_i]$$

$$\Sigma\equiv l_{-i}+U_i^{\max}+\alpha$$

A similar adiabatic assumption is made on [RM$_i$]. Assuming [R] and [M$_i$] vary on a timescale >>1/Σ (again, we assume they change on a cell cycle timescale), then on a timescale > 1/ Σ we have from (B.11):

(B.15) $[RM_i]^{SS}\simeq\frac{1}{L_{m,i}}[R][M_i]$

Where $L_{m,i}\equiv\left(l_{-i}+U_i^{\max}+\alpha\right)/l_i\cong\left(l_{-i}+U_i^{\max}\right)/l_i$

and as before, 1/ Σ is governed by the fastest rate. To estimate Σ we note that $L_{m,bulk}$~7 μM (Table S3), $U^{max}{}_{bulk}$~100 ini/min (Table S1) and $l_{-1,bulk}$ is set by the diffusion limit: $l_{1,bulk}$~10^9 (M sec)$^{-1}$ [86] then $1/\Sigma \sim 1/l_{-1,bulk} \sim \mathrm{msec}$.

[Pd$_i$] assumes pseudo-steady state on a timescale > ~1 msec, and assuming it changes on a timescale >>1/t$_{c,i}$ ~ minutes then we have on a timescale > ~1/t$_{c,i}$ pseudo-steady state conditions for (B.5):

(B.16) $[P\mathbf{M}_i]\simeq t_{c,i}V_i^{\max}[Pd_i]$

Similarly, [RM$_i$] assumes pseudo-steady state on a timescale > ~1msec, and assuming it changes on a timescale >>1/t$_{l,i}$ <1 min then we have on a timescale > 1/t$_{l,i}$ pseudo-steady state conditions for (B.7):

(B.17) $[R\mathbf{X}_i]\simeq t_{l,i}U_i^{\max}[RM_i]$

Decay of mRNA

We would like to show that M_i^{tot} decays exponentially and obtain an expression for the chemical half-life for this mRNA.

$$\frac{dM_i^{tot}}{dt}=\frac{d[M_i\varnothing]}{dt}+\frac{d[RM_i]}{dt}=\frac{1}{t_{c,i}}[P\mathbf{M}_i]-\alpha M_i^{tot}-W_i^{\max}[M_i\circ\text{RNase}]$$

Assuming RNase complexes $[M_i\circ\text{RNase}]$ reach pseudo steady state in (B.9) we have

$$[M_i\circ\text{RNase}]=\frac{1}{J_{m,i}}[M_i][\text{RNase}]$$

$$J_{m,i}=\left(j_{-i}+W_i^{\max}\right)/j_i$$

Thus

$$[M_i]=\frac{J_{m,i}}{[\text{RNase}]}[M_i\circ\text{RNase}]$$

$$M_i^{tot}=[M_i]+[RM_i]+[M_i\circ\text{RNase}]=[M_i]+\frac{1}{L_{m,i}}[R][M_i]+[M_i\circ\text{RNase}]=\frac{J_{m,i}}{[\text{RNase}]}[M_i\circ\text{RNase}]\left(1+\frac{[R]}{L_{m,i}}\right)+[M_i\circ\text{RNase}]$$

$$M_i^{tot}=[M_i\circ\text{RNase}]\left[\frac{J_{m,i}}{[\text{RNase}]}\left(1+\frac{[R]}{L_{m,i}}\right)+1\right]\Rightarrow$$

$$[M_i\circ\text{RNase}]=\left[\frac{J_{m,i}}{[\text{RNase}]}\left(1+\frac{[R]}{L_{m,i}}\right)+1\right]^{-1}M_i^{tot}$$

Inserting this result into (B.12):

$$\frac{dM_i^{tot}}{dt}=\frac{1}{t_{c,i}}[P\mathbf{M}_i]-\alpha M_i^{tot}-W_i^{\max}[M_i\circ\text{RNase}]$$

we obtain

$$\frac{dM_i^{tot}}{dt}=\frac{1}{t_{c,i}}[P\mathbf{M}_i]-\left(\alpha+\frac{1}{\tau_{chem,i}}\right)M_i^{tot}\cong\frac{1}{t_{c,i}}[P\mathbf{M}_i]-\frac{1}{\tau_{chem,i}}M_i^{tot}$$

Where $\tau_{chem,i}$ is the effective chemical life-time of the mRNA:

$$\text{(B.18)}\quad \tau_{chem,i}\equiv\frac{1}{W_i^{\max}}\left[\frac{J_{m,i}}{[\text{RNase}]}\left(1+\frac{[R]}{L_{m,i}}\right)+1\right]=\frac{1}{W_i^{\max}}\left[\frac{J_{m,i}}{J_1}\left(1+\frac{[R]}{L_{m,i}}\right)+1\right]$$

where in the last equality we used the fact the RNase E is negatively autoregulated (see main text and S2.7).

[P**M**i] assumes pseudo steady state on a time scale of ~min. Assuming [P**M**i] changes on a timescale >> $\tau_{chem,i}\sim$ min then M_i^{tot} reaches pseudo steady state. Therefore we can assume (B.12) reaches pseudo steady state:

(B.19) $[M_i \circ \text{RNase}] \simeq \frac{1}{W_i^{\max} t_{c,i}}[P\mathbf{M}_i] \simeq \frac{V_i^{\max}}{W_i^{\max}}[Pd_i]$

or

(B.20) $[M_i] = \frac{J_{m,i}\frac{V_i^{\max}}{W_i^{\max}}[Pd_i]}{[\text{RNase}]}$

$$[RM_i] \approx \frac{1}{L_{m,i}}[R][M_i] = \frac{[R]}{L_{m,i}} \frac{J_{m,i}\frac{V_i^{\max}}{W_i^{\max}}[Pd_i]}{[\text{RNase}]}$$

$$[R\mathbf{X}_i] \simeq t_{l,i}U_i^{\max}[RM_i] = t_{l,i}U_i^{\max}\frac{[R]}{L_{m,i}} \frac{J_{m,i}\frac{V_i^{\max}}{W_i^{\max}}[Pd_i]}{[\text{RNase}]}$$

Derivation of Michaelis-Menten kinetics

Using the promoter conservation equation (B.3):

$$\left.\begin{array}{l} [Pd_i]^{SS} = \frac{1}{K_{m,i}}[P][d_i] \\ d_i^{tot} = [d_i] + [Pd_i] \end{array}\right\} \Rightarrow$$

(B.21) $[Pd_i]^{SS} = \frac{d_i^{tot}[P]}{[P] + L_{m,i}} \equiv d_i^{tot}\theta_{k,i}$

Total mRNA

Note that the total mRNA concentration of gene class i is given by

$$M_i^{tot} = \frac{\tau_{chem,i}}{t_{c,i}}[P\mathbf{M}_i] = \frac{\tau_{chem,i}}{t_{c,i}}\left(V_i^{\max} t_{c,i}[Pd_i]\right) = \tau_{chem,i}V_i^{\max} d_i^{tot}\theta_{k,i}$$

Total ribosomes

$$\frac{dR^{tot}}{dt} = \frac{d[R]}{dt} + \sum_{i=1,2,4}\left(\frac{d[RM_i]}{dt} + \frac{d[R\mathbf{X}_i]}{dt}\right)$$

$$\frac{d[R\mathbf{X}_i]}{dt} = U_i^{\max}[RM_i] - \left(\frac{1}{t_{l,i}} + \alpha\right)[R\mathbf{X}_i]$$

$$\frac{d[RM_i]}{dt} = l_i[R][M_i] - (l_{-i} + U_i^{\max} + \alpha)[RM_i]$$

$$\frac{d[R]}{dt} = \frac{1}{t_{c,rrn}}[P\mathbf{M}_{rrn}] + \sum_{i=1,2,4}\left[\frac{1}{t_{l,i}}[R\mathbf{X}_i] + l_{-i}[RM_i] - l_i[M_i][R]\right] - \alpha[R]$$

$$\frac{dR^{tot}}{dt} = \frac{1}{t_{c,rrn}}[P\mathbf{M}_{rrn}] - \alpha R^{tot}$$

Next, from (B.16) and (B.21) and the equation just derived we find that

(B.22) $$\frac{dR^{tot}}{dt} \simeq V_{rrn}^{\max} d_{rrn}^{tot} \theta_{k,rrn} - \alpha R^{tot}$$

Total bulk concentration

$$\frac{d[X_i]}{dt} = \frac{1}{t_{l,i}}[R\mathbf{X_i}] - (\alpha + \gamma_i)[X_i]$$

and for i=1: $\frac{d[B]}{dt} = \frac{1}{t_{l,bulk}}[R\mathbf{B}] - \alpha[B]$

Assuming pseudo steady state value for [R**B**] we find that

(B.23) $$\frac{d[B]}{dt} = U_{bulk}^{\max} \frac{[R]}{L_{m,bulk}} \frac{J_{m,bulk}}{J_1} \frac{V_{bulk}^{\max}}{W_{bulk}^{\max}} d_{bulk}^{tot} \theta_{k,bulk} - \alpha[B]$$

Where we assumed [RNase]=J_1 due to negative autoregulation of RNase E.

Note that the equation above can be written as (multiplying and dividing by $[R] + L_{D,bulk}$)

$$\frac{d[B]}{dt} = \left[\frac{1}{W_{bulk}^{\max}} \left(1 + \frac{[R]}{L_{m,bulk}} \right) \frac{J_{m,bulk}}{J_1} \right] U_{bulk}^{\max} V_{bulk}^{\max} d_{bulk}^{tot} \theta_{k,bulk} \theta_{l,bulk} - \alpha[B] = \tau_{fun,bulk} U_{bulk}^{\max} V_{bulk}^{\max} d_{bulk}^{tot} \theta_{k,bulk} \theta_{l,bulk} - \alpha[B]$$

where

(B.24) $$\tau_{fun,bulk} \equiv \frac{1}{W_{bulk}^{\max}} \frac{J_{D,bulk}}{J_1} \left(1 + \frac{[R]}{L_{D,bulk}} \right)$$

We identify $\tau_{fun,bulk}$ as the functional half-life (divided by ln 2) of the mRNA, and $U_{bulk}^{\max} \theta_{l,bulk}$ as the translation initiation rate. Therefore $\tau_{fun,bulk} U_{bulk}^{\max} \theta_{l,bulk}$ will be the average number of proteins synthesized from a single mRNA, and $V_{bulk}^{\max} \theta_{l,bulk}$ the mRNA synthesis rate. See S2.7 for further comments.

Conservation equations revisited

The conservation equation for RNAp at steady state is given by (c.f. Eq. B.1):

$$P^{tot} \simeq [P] + \sum_{i=1}^{4} t_{c,i} V_i^{\max} [Pd_i] \approx [P] + \sum_{i=1}^{4} t_{c,i} V_i^{\max} [Pd_i]^{SS}$$

This equation can now be written as

(B.25) $$P^{tot} \simeq [P] + \sum_{i=1}^{3} t_{c,i} V_i^{\max} d_i^{tot} \theta_{k,i}$$

Where we neglect the contribution of RNAp (see main text).

The conservation equation for ribosomes at steady state is given by (c.f. Eq. B.2):

$$R^{tot} \simeq [R] + \sum_{i=1,2,4} t_{l,i} U_i^{\max} [RM_i] \approx [R] + \sum_{i=1,2,4} t_{l,i} U_i^{\max} [RM_i]^{SS}$$

Now since r-protein production rate is regulated so that it matches rRNA production rate, we have

$$\text{r-protein production rate} = \frac{1}{t_{l,2}} [R \circ \text{r-protein}] = U_{r-protein}^{\max} [RM_{r-protein}] \overset{!}{=} \text{rRNA production rate} = \frac{1}{t_{c,rrn}} [PM_{rrn}] = V_{rrn}^{\max} d_{rrn}^{tot} \theta_{k,rrn} \Rightarrow$$

$$t_{l,2} U_{r-protein}^{\max} [RM_{r-protein}] \overset{!}{=} t_{l,protein} V_{rrn}^{\max} d_{rrn}^{tot} \theta_{k,rrn}$$

Where this equality is satisfied by virtue of the regulation of r-protein translation initiation rate and half-life [2,8]. Therefore we obtain the following conservation equation for ribosomes:

$$\text{(B.26)} \quad R^{tot} \simeq [R] + \sum_{i=1,4} t_{l,i} U_i^{\max} \frac{[R]}{L_{m,i}} \frac{J_{m,i}}{J_1} \frac{V_i^{\max}}{W_i^{\max}} d_i^{tot} \theta_{k,i} + t_{l,r-protein} V_{rrn}^{\max} d_{rrn}^{tot} \theta_{k,rrn}$$

Final rate equations

$$\text{(B.27)} \quad \begin{aligned} &P^{tot} \approx \text{const} \\ &\frac{d[B]}{dt} = U_{bulk}^{\max} \frac{[R]}{L_{m,bulk}} \left(\frac{J_{m,bulk}}{J_1} \frac{V_{bulk}^{\max}}{W_{bulk}^{\max}} \right) d_{bulk}^{tot} \theta_{k,bulk} - \alpha [B] \\ &\frac{dR^{tot}}{dt} = V_{rrn}^{\max} d_{rrn}^{tot} \theta_{k,rrn} - \alpha R^{tot} \\ &P^{tot} \simeq [P] + \sum_{i=1}^{3} t_{c,i} V_i^{\max} d_i^{tot} \theta_{k,i} \\ &R^{tot} \simeq [R] + \sum_{i=1,4} t_{l,i} U_i^{\max} \frac{[R]}{L_{m,i}} \frac{J_{m,i}}{J_1} \frac{V_i^{\max}}{W_i^{\max}} d_i^{tot} \theta_{k,i} + t_{l,r-protein} V_{rrn}^{\max} d_{rrn}^{tot} \theta_{k,rrn} \end{aligned}$$

Steady state equations (d/dt=0)

$$P^{tot} \approx \text{const}$$

$$[B] = \frac{1}{\alpha} U_{bulk}^{\max} \frac{[R]}{L_{m,i}} \left(\frac{J_{m,bulk}}{J_1} \frac{V_{bulk}^{\max}}{W_{bulk}^{\max}} \right) d_{bulk}^{tot} \theta_{k,bulk}$$

$$R^{tot} = \frac{1}{\alpha} V_{rrn}^{\max} d_{rrn}^{tot} \theta_{k,rrn}$$

$$P^{tot} \simeq [P] + \sum_{i=1}^{3} t_{c,i} V_i^{\max} d_i^{tot} \theta_{k,i}$$

$$R^{tot} \simeq [R] + \alpha \left(t_{l,RNAp} P^{tot} + t_{l,bulk} B + t_{l,r-protein} R^{tot} \right)$$

S2.6 Summary of the CGGR model equations

S2.6.1 The unconstrained CGGR

Neg. auto reg. of RNAp	(1)	$n_{RNAp} \approx \text{const}$
Bulk synthesis	(2)	$n_{bulk} = \frac{1}{\alpha} V_{bulk} \tilde{U}_{bulk} d_{bulk} \tau^0_{bulk,fun}$
Load genes synthesis	(3)	$n_{load\ i} = \frac{1}{\alpha} V_{load\ i} \tilde{U}_{load\ i} d_{load\ i} \tau^0_{load\ i,fun}$
Ribosome synthesis	(4)	$n_{ribo} = \frac{1}{\alpha} V_{rrn} d_{rrn}$
RNAp conservation	(5)	$n_{RNAp} = n_{RNAp,free} + t_{c,bulk} d_{bulk} V_{bulk} + t_{c,r-protein} d_{r-protein} V_{r-protein} + t_{c,rrn} V_{rrn} d_{rrn} + \sum_i t_{c,load\ i} V_{load\ i} d_{load\ i} + (1 - e^{-\alpha \tau_{RNAp}}) n_{RNAp}$
Ribosome conservation	(6)	$n_{ribo} = n_{ribo,free} + \alpha \left(t_{l,bulk} n_{bulk} + t_{l,r-prot} n_{ribo} + \sum_i t_{l,load\ i} n_{load\ i} \right) + (1 - e^{-\alpha \tau_{ribo}}) n_{ribo}$
Cost criterion	(7)	$n_{bulk} = n_0 + c_{ribo} n_{ribo} + \sum_i c_{load\ i} n_{load\ i}$

Cooper-Helmstetter model assuming constant initiation volume:

$d_i(\mu) = \frac{1}{\ln 2 V_{ini}} \sum_j 2^{-m_j^{(i)} C\mu}$ (V_{ini},C=const; see Eq. 3)

and where

$(\ln 2 V_{ini})^{-1} \equiv \text{constant} = 2^{\mu_0 (C+D)} / V_{cell}(\mu_0)$, $\mu_0 = WT$ growth rate (see Table S1 footnote d)

$\tau^0_{i,fun} \equiv \frac{1}{W_i^{\max}} \frac{J_{m,i}}{J_1} = \text{constant} = \tau_{fun,i}(WT) \left(1 + \frac{n_{ribo,free}(WT)}{L_{m,i}} \right)^{-1}$ (see Eq. S15/B.24)

$K_{m,i} = K_{m,i}(\Phi), L_{m,i} = L_{m,i}(\Phi)$ (see Eq. S11 or S12)

where

$$\Phi \equiv v_{ribo} n_{ribo} + v_{bulk} n_{bulk} + \sum_i v_{load\ i} n_{load\ i}$$

$$V_i = V_i^{\max} \frac{n_{RNAp,free}}{n_{RNAp,free} + K_{m,i}}; \quad \tilde{U}_i = U_i^{\max} \frac{n_{ribo,free}}{L_{m,i}}$$

For a list of notation used see Table S8.

For convenience all concentrations were multiplied by the WT cell volume, $V_{cell}(\mu_0)$. In the simulation, $V_{cell}(\mu_0)$ was defined to be the effective volume for which Φ(WT)=0.34.

S2.6.2 The constrained CGGR

Neg. auto reg. of RNAp	(1)	$n_{RNAp} \approx \text{const}$
Bulk synthesis	(2)	$n_{bulk} = \frac{1}{\alpha} V_{bulk} \tilde{U}_{bulk} d_{bulk} \tau^0_{bulk,fun}$
Load genes synthesis	(3)	$n_{load\ i} = \frac{1}{\alpha} V_{load\ i} \tilde{U}_{load\ i} d_{load\ i} \tau^0_{i\ load,fun}$
Ribosome synthesis	(4)	$n_{ribo} = \frac{1}{\alpha} V_{rrn} d_{rrn}$
RNAp conservation	(5)	$n_{RNAp} = n_{RNAp,free} + t_{c,bulk} d_{bulk} V_{bulk} + t_{c,r-protein} d_{r-protein} V_{r-protein} + t_{c,rrn} V_{rrn} d_{rrn} + \sum_i t_{c,load\ i} V_{load\ i} d_{load\ i} + (1 - e^{-\alpha \tau_{RNAp}}) n_{RNAp}$
Ribosome conservation	(6)	$n_{ribo} = n_{ribo,free} + \alpha \left(t_{l,bulk} n_{bulk} + t_{l,r-prot} n_{ribo} + \sum_i t_{l,load\ i} n_{load\ i} \right) + (1 - e^{-\alpha \tau_{ribo}}) n_{ribo}$
Constant macro. molec. volume fraction	(7)	$\Phi \equiv v_{ribo} n_{ribo} + v_{bulk} n_{bulk} + \sum_i v_{load\ i} n_{load\ i} = \text{const}$

Cooper-Helmstetter model assuming constant initiation volume:

$d_i(\mu) = \frac{1}{\ln 2 V_{ini}} \sum_j 2^{-m_j^{(i)} C \mu}$ (V_{ini},C=const; see Eq. 3)

and where

$(\ln 2 V_{ini})^{-1} \equiv \text{constant} = 2^{\mu_0 (C+D)} / V_{cell}(\mu_0)$, $\mu_0 = WT$ growth rate (see also Table S1 footnote d)

$\tau^0_{i,fun} \equiv \frac{1}{W^{\max}_{bulk}} \frac{J_{m,i}}{J_1} = \text{constant} = \tau_{fun,i}(WT) \left(1 + \frac{n_{ribo,free}(WT)}{L_{m,i}} \right)^{-1}$ (see Eq. S15/B.24)

$c_p = c_p^{\max} \Big/ \left[1 + \left(M_{bulk} / n_{bulk} \right)^n \right]$ (implicit in $t_{l,i}$ in Eq. 6 above)

$V_i = V_i^{\max} \frac{n_{RNAp,free}}{n_{RNAp,free} + K_{m,i}}$; $\tilde{U}_i = U_i^{\max} \frac{n_{ribo,free}}{L_{m,i}}$

Since the equations of state for both models are a set of coupled nonlinear equations, in principle more than one solution may exist (which may or may not be biologically relevant). Therefore the initial guess for the WT genome configuration (and also WT+1) was in the proximity of the WT cell state. After the WT genome configuration was solved, strains with increasing or decreasing *rrn* copy number were solved. For each new strain, the solution for the previous strain (with a copy number closer by 1 to the WT copy number) was used as an initial guess. The equations were solved numerically with the trust-region dogleg method implemented in Matlab 7.

S2.7 mRNA degradation

S2.7.1 mRNA degradation via RNase E

mRNA degradation of most mRNA in *E. coli* is thought to be initiated by RNase E [87,88], which is generally considered to be the partially or fully rate-determining step in the degradation process [87-92]. This process is assumed to follow Michaelis-Menten like kinetics [93,94]. It is thought that ribosomes bound to RBS protect the mRNA from degradation by preventing RNase E from binding to the 5' end or cleaving the mRNA (see S2.7.2; for recent reviews see [28,29,55]). An internal entry path for RNase E - not via 5' end- is thought to be a less efficient [88]. In the 5' competition model presented in Fig. 1 and Eq. R3 in S2.5.2, ribosome protection is achieved by assuming that RNase E competes with 30S subunits in binding to the RBS [29,55,95] functionally inactivating it when it binds [55] (therefore the RNase in Eq. R3 binds only to M_i and not RM_i). Another possibility is that mRNA becomes functionally inactive only upon cleavage, suggesting that most mRNA are nucleolytically inactivated as advocated in [29]. We also analyzed this scenario and found no fundamental difference (see S2.7.4). rRNA and tRNA molecules are protected from RNase E and remain intact for several generations [88]. Note that in the scheme presented in (R1)-(R3) (c.f. S2.5.2) we made the approximation that once a peptide chain is initiated it will continue to elongate until the full peptide chain is formed, i.e. there is no degradation term for R**B**.

S2.7.2 mRNA half-life based on the 5' competition model

By writing out the full kinetic reactions in Fig. 1B one can obtain an expression for the functional and chemical half-life of bulk mRNA. The full derivation is carried out in S2.5.2 and here we will only interpret these results.

By defining U_{bulk} according to Michaelis-Menten kinetics in Eq. 1, we can identify in the expression for bulk protein synthesis (Eq. 2ii) the functional lifetime of bulk mRNA, $\tau_{fun,bulk} = T_{1/2,bulk}^{fun} / \ln 2$, such that the protein synthesis rate is given by $V_{bulk} U_{bulk} d_{bulk} \tau_{fun,bulk}$ (c.f. Eq. B.24 in S2.5.2). In S2.5.2 we also obtain an expression for the chemical lifetime $\tau_{chem,bulk}$ of bulk mRNA, defined as the degradation rate of total bulk mRNA (Eq. B.18). Generalizing these expressions for the *i*-th gene class we obtain the following expressions

$$\tau_{fun,i} = \frac{1}{W_i^{\max}} \frac{J_{m,i}}{J_1} \left(1 + \frac{n_{ribo,free}}{L_{m,i}} \right) \equiv \tau_{fun,i}^0 \left(1 + \frac{n_{ribo,free}}{L_{m,i}} \right) < \tau_{chem,i} = \frac{1}{W_i^{\max}} \left[\frac{J_{m,i}}{J_1} \left(1 + \frac{n_{ribo,free}}{L_{m,i}} \right) + 1 \right] \quad \text{(S15)}$$

Where we defined $\tau^{0}_{fun,i} \equiv J_{m,i} \big/ \left(J_1 W_i^{\max}\right)$ and with J_1 is given by

$$J_1 \equiv n_{\text{RNase E,free}} = n_{\text{RNase E}} - n_{\text{RNase E,bound}} \tag{S16}$$

and where $J_{m,i}$ is the binding affinity of RNase E to the i-th gene class mRNA and $W_i^{\max}$ is the maximal Michaelis-Menten rate of degradation of the i-th gene class mRNA (see Fig. 1/Eq. R3). Thus, mRNA half-life increases with the concentration of free ribosomes. As expected, the functional half-life is shorter than the chemical half-life [29]. Before presenting experimental support for Eq. S15, we pause to further discuss this equation. It has been proposed that RNase E is negatively autoregulated [90,96,97] such that it adjusts its expression to that of its substrates, thus limiting the pool of free RNase E [96]. In the present model we assume that that this autoregulation is ideal [86] and therefore the free pool of RNase E ($n_{\text{RNase E,free}}$) is held fixed at some value of J_1. J_1 can be interpreted as the binding affinity of RNase E to its own mRNA. Since J_1 is fixed, the true genetic parameter is not $\tau_{fun,i}$, but rather $\tau^{0}_{fun,i}$ ($T^{fun,o}_{1/2,i} \big/ \ln 2$ in the main text). Therefore we can express the protein synthesis rate as $V_i \, \tilde{U}_i \, d_i \tau^{0}_{fun,i}$, where $\tilde{U}_i = U_i^{\max} \dfrac{n_{ribo,free}}{L_{m,i}}$. Thus at steady state $n_i = \dfrac{1}{\alpha} V_i \, \tilde{U}_i \, d_i \tau^{0}_{i,fun}$ (c.f. S2.6.1).

Note that had we taken the mRNA half-life to be a constant, then one would find that $n_i \sim n_{ribo,free} \big/ \left(n_{ribo,free} + L_{m,i}\right)$ as would be expected from a Michaelis-Menten type interaction. However the expression above reveals that $\tau_{fun,i} \sim \left(n_{ribo,free} + L_{m,i}\right) \big/ L_{m,i}$ and therefore $n_i \sim n_{ribo,free} \big/ L_{m,i}$ that diverges from the previous result when $n_{ribo,free} \gtrsim L_{m,i}$. This result suggests that due to the degradation-translation coupling, within the limits of this model, protein synthesis is linear in the concentration of free ribosomes. Of course as the concentration of free ribosomes increases, half-life increases until at some point the adiabatic approximation should fail.

Equation S15 predicts that bulk mRNA half-life depends on the RBS binding affinity $L_{m,bulk}$ but does not depend explicitly on the translation initiation frequency (e.g. it does not depend on $U_{bulk}^{\max}$). In addition, mRNA half-life increases with the degree of RBS occupancy. This is in agreement with experimental observation examining the influence of RBS mutations on RNA decay [28]. These

studies suggest that mutations to the RBS that cause interference with ribosome binding, lead to instability of the mRNA, while mutations that improve the RBS prolong mRNA longevity (see discussion in [28]), just as Eq. S15 predicts. Furthermore, it is thought that ribosomes mainly protect mRNA from cleavage by binding to the RBS as opposed to conferring steric protection across the entire mRNA [28]. By binding to the RBS, ribosomes are thought to prevent access of RNase E to certain features at the 5' end that control access to internal RNase E cleavage sites [28]. To demonstrate this, it has been shown that "mutating the initiation codon so as to reduce the frequency of translation initiation (and thereby increase ribosomal spacing) without significantly diminishing the ribosome-binding affinity of the RBS and its average occupancy by ribosomes" leads to "more often than not … only a modest decrease in mRNA half-life" [28]. Based on these findings the authors conclude that "a high degree of RBS occupancy by ribosomes is usually more important for mRNA longevity than is close spacing of translating ribosomes" [28] (also c.f. references quoted therein). This is in agreement with Eq. S15 since the mRNA half-life depends on the RBS affinity but does not depend explicitly on the translation initiation frequency. If $p_{\mathrm{RBS\ bound}} = 1/(1 + L_{m,i}/n_{ribo,free})$ is the probability that a RBS is occupied, then the rate of functional inactivation (from Eq. S15) can be expressed as $1/\tau_{fun,i} = \left(1/\tau^0_{i,fun}\right) p_{\mathrm{RBS\ vacant}}$ where $p_{\mathrm{RBS\ vacant}} = 1 - p_{\mathrm{RBS\ bound}}$, and $1/\tau^0_{i,fun}$ is the functional (maximal) inactivation rate of mRNA in the absence of ribosomes.

S2.7.3 mRNA degradation should not be affected by crowding

One may suspect that crowding would affect the binding of RNase E (118 kD [98]) to mRNA and modulate the degradation rate. However, this does not appear to be the case. The equilibrium constant of RNase E binding to the *i*-th gene class mRNA, $J_{m,i}$, appearing in Eq. S15, is divided by the equilibrium constant of RNase E binding to it's own mRNA, J_1. Since both these constants should be affected similarly by crowding, the mutual effects of crowding should tend to cancel out, thanks to the negative autoregulation of RNase E.

S2.7.4 Model assuming nucleolytic inactivation of mRNA

In this degradation model we assume that RNase E bound mRNA is still functionally active, and that inactivation occurs only upon degradation, i.e. functional inactivation occurs upon physical inactivation. Thus Eq. R.2 in S2.5.2 is replaced with the following reaction

$$[P]+[d_{bulk}] \underset{k_{-1,bulk}}{\overset{k_{1,bulk}}{\rightleftharpoons}} [Pd_{bulk}] \xrightarrow{V_{bulk}^{\max}} [P\mathbf{M_{bulk}}]_{+[d_{bulk}]} \xrightarrow{1/t_{c,,bulk}} [M_{bulk}\varnothing]_{+[P]} \Rightarrow$$

$$[M_{bulk}\varnothing]^{+[R]} \underset{l_{-1,bulk}}{\overset{l_{1,bulk}}{\rightleftharpoons}} [RM_{bulk}\varnothing] \xrightarrow{U_i^{\max}} [R\mathbf{X_{bulk}}]_{+[M_{bulk}\varnothing]} \xrightarrow{1/t_{l,bulk}} [R]+[X_{bulk}]^{+[\text{Protease}]} \xrightarrow{\gamma_{bulk}} [\text{Protease}]+\text{decay fragments}$$

Where we defined

$\varnothing$ ≡ with or without RNase E

Following a similar derivation to the one presented above it can be shown that for this scheme

$$\tau_{chem,i} = \tau_{fun,i} = \frac{1}{W_i^{\max}}\left(1+\frac{[R]}{L_{m,i}}\right)\left(1+\frac{J_{m,i}}{J_1}\right)$$

As expected the chemical and functional half-lives are identical [29]. As in Eq. S15, the half-life depends on $J_{m,i}/J_1$, which is a constant (expected to be independent of Φ, c.f. S2.7.3), thus there is no fundamental difference between these two models.

S3 The simplified model

S3.1 Analytical solution

The analytical solution to the simplified 3-state model (Eq. 7) is given in Eq. S17.

$$
\begin{aligned}
&(i)\quad \mu = \frac{|c_{ribo}|\, g_{rrn}}{2n_0}\left(\sqrt{1+\frac{\gamma_{bulk}}{g_{rrn}c_{ribo}^2}} - sign\left(c_{ribo}\right)\right) \\
&(ii)\quad n_{ribo} = \frac{2n_0}{|c_{ribo}|}\left(\sqrt{1+\frac{\gamma_{bulk}}{g_{rrn}c_{ribo}^2}} - sign\left(c_{ribo}\right)\right)^{-1} \qquad\qquad (S17) \\
&(iii)\quad n_{bulk} = n_0\left[1+2\cdot\left(-1+sign\left(c_{ribo}\right)\sqrt{1+\frac{\gamma_{bulk}}{g_{rrn}c_{ribo}^2}}\right)^{-1}\right]
\end{aligned}
$$

where $\gamma_{bulk} \equiv 4n_0 g_{bulk}$.

S3.2 Approximations made

In the case of the *rrn* inactivation strains, where crowding effects are moderate and Φ<0.34, the following simplifying assumptions can be introduced: (i) Crowding effects can be disregarded due to the free RNAp/free ribosome self-adjusting mechanism (Fig. S4A) (ii) $n_{RNAp,free}/K_{m,i} \approx \text{const}$ (Fig. S4A) (iii) $n_{ribo,free}/L_{m,bulk} \propto n_{ribo}$ (and thus $n_{bulk} \sim n_{ribo}$) (Fig. S11) (iv) $d_i \approx \text{const}$ (Fig. S7B); (iv) *lacZ* and *cat* expression are neglected (together they constitute at most ~10% of the bulk protein). (iv) Immature pools, which are about 10% of total complex (c.f. S2.5.1), are neglected. Under these approximations, Eq. 2 reduces to Eq. 7.

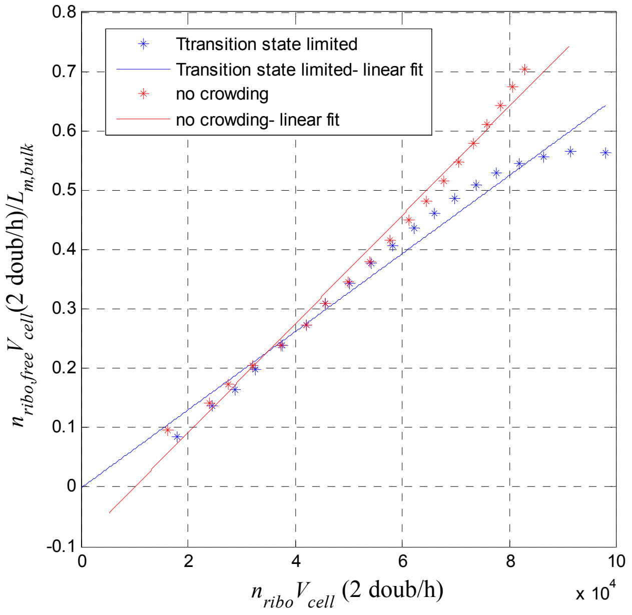

Figure S11. Relation between free ribosome and total ribosome concentration. $n_{ribo,free}/L_{m,bulk}$ vs. n_{ribo} for the unconstrained CGGR in the transition state limited scenario and the no crowding scenario, based on the simulation of *rrn* inactivation strains of Asai et al. [9]. *rrn* copy number was varied between 1 and 19. Concentrations were multiplied by the cell volume at 2 doub/h.

References

1. Bremer H, Dennis P, Ehrenberg M (2003) Free RNA polymerase and modeling global transcription in Escherichia coli. Biochimie 85: 597-609.
2. Keener J, Nomura M (1996) Regulation of ribosome synthesis. In: Neidhardt FC, Curtiss RIII, Ingraham JL, Lin ECC, Low KB et al., editors. Escherichia coli and Salmonella typhimurium: cellular and molecular biology. 2nd ed. Washington, D.C: ASM Press. pp. 1417–1431.
3. Bremer H, Dennis PP (1996) Modulation of Chemical Composition and Other Parameters of the Cell by Growth Rate. In: Neidhardt FC, Curtiss RIII, Ingraham JL, Lin ECC, Low KB et al., editors. Escherichia coli and Salmonella typhimurium: cellular and molecular biology. 2nd ed. Washington, D.C: ASM Press. pp. 1553-1569.
4. Condon C, French S, Squires C, Squires CL (1993) Depletion of Functional Ribosomal-Rna Operons in Escherichia-Coli Causes Increased Expression of the Remaining Intact Copies. Embo Journal 12: 4305-4315.
5. Selinger DW, Saxena RM, Cheung KJ, Church GM, Rosenow C (2003) Global RNA half-life analysis in Escherichia coli reveals positional patterns of transcript degradation. Genome Research 13: 216-223.
6. Darge W (2004) The Ribosome: Protein Manufacturer, An Introduction Ribosomen Verlag.
7. Neidhardt FC, Ingraham JL, Schaechter M (1990) Physiology of the Bacterial Cell: A Molecular Approach: Sinauer Associates Inc.
8. Dennis PP, Ehrenberg M, Bremer H (2004) Control of rRNA synthesis in Escherichia coli: a systems biology approach. Microbiol Mol Biol Rev 68: 639-668.
9. Asai T, Condon C, Voulgaris J, Zaporojets D, Shen BH, et al. (1999) Construction and initial characterization of Escherichia coli strains with few or no intact chromosomal rRNA operons. Journal of Bacteriology 181: 3803-3809.
10. Zimmerman SB, Trach SO (1991) Estimation of Macromolecule Concentrations and Excluded Volume Effects for the Cytoplasm of Escherichia-Coli. Journal of Molecular Biology 222: 599-620.
11. Dykxhoorn DM, StPierre R, Linn T (1996) Synthesis of the beta and beta' subunits of Escherichia coli RNA polymerase is autogenously regulated in vivo by both transcriptional and translational mechanisms. Molecular Microbiology 19: 483-493.
12. Shepherd N, Dennis P, Bremer H (2001) Cytoplasmic RNA polymerase in Escherichia coli. Journal of Bacteriology 183: 2527-2534.
13. de Smit MH, Duin Jv (2003) Ribosomes on standby: a prelude to translational (re)initiation. In: Lapointe J, Brakier-Gingras L, editors. Translation mechanisms. 1 edition ed. Georgetown, TX: Landes Bioscience. pp. 298-321.
14. Vind J, Sorensen MA, Rasmussen MD, Pedersen S (1993) Synthesis of Proteins in Escherichia-Coli Is Limited by the Concentration of Free Ribosomes - Expression from Reporter Genes Does Not Always Reflect Functional Messenger-Rna Levels. Journal of Molecular Biology 231: 678-688.
15. Grunberg-Manago M (1996) Regulation of the expression of aminoacyl-tRNA synthetases and translation factors. In: Neidhardt FC, Curtiss RIII, Ingraham JL, Lin ECC, Low KB et al., editors. Escherichia coli and Salmonella typhimurium: cellular and molecular biology. 2nd ed. Washington, D.C: ASM Press. pp. 1432-1457.
16. Condon C, Liveris D, Squires C, Schwartz I, Squires CL (1995) Ribosomal-Rna Operon Multiplicity in Escherichia-Coli and the Physiological Implications of Rrn Inactivation. Journal of Bacteriology 177: 4152-4156.
17. Forchham.J, Lindahl L (1971) Growth Rate of Polypeptide Chains as a Function of Cell Growth Rate in a Mutant of Escherichia-Coli 15. Journal of Molecular Biology 55: 563-&.

18. Sorensen MA, Pedersen S (1991) Absolute in vivo translation rates of individual codons in Escherichia coli. The two glutamic acid codons GAA and GAG are translated with a threefold difference in rate. J Mol Biol 222: 265-280.
19. Kennell D, Riezman H (1977) Transcription and Translation Initiation Frequencies of Escherichia-Coli Lac Operon. Journal of Molecular Biology 114: 1-21.
20. Neidhardt FC, Umbarger HE (1996) Chemical Composition of Escherichia coli In: Neidhardt FC, Curtiss RIII, Ingraham JL, Lin ECC, Low KB et al., editors. Escherichia coli and Salmonella typhimurium: cellular and molecular biology. 2nd ed. Washington, D.C: ASM Press. pp. 13-16.
21. Zhang X, Dennis P, Ehrenberg M, Bremer H (2002) Kinetic properties of rrn promoters in Escherichia coli. Biochimie 84: 981-996.
22. Condon C, Philips J, Fu ZY, Squires C, Squires CL (1992) Comparison of the Expression of the 7 Ribosomal-Rna Operons in Escherichia-Coli. Embo Journal 11: 4175-4185.
23. Ellwood M, Nomura M (1980) Deletion of a Ribosomal Ribonucleic-Acid Operon in Escherichia-Coli. Journal of Bacteriology 143: 1077-1080.
24. Brosius J (1984) Plasmid Vectors for the Selection of Promoters. Gene 27: 151-160.
25. Close TJ, Rodriguez RL (1982) Construction and Characterization of the Chloramphenicol-Resistance Gene Cartridge - a New Approach to the Transcriptional Mapping of Extrachromosomal Elements. Gene 20: 305-316.
26. Asai T, Zaporojets D, Squires C, Squires CL (1999) An Escherichia coli strain with all chromosomal rRNA operons inactivated: Complete exchange of rRNA genes between bacteria. PNAS 96: 1971-1976.
27. Voulgaris J, Pokholok D, Holmes WM, Squires C, Squires CL (2000) The feedback response of Escherichia coli rRNA synthesis is not identical to the mechanism of growth rate-dependent control. Journal of Bacteriology 182: 536-539.
28. Deana A, Belasco JG (2005) Lost in translation: the influence of ribosomes on bacterial mRNA decay. Genes & Development 19: 2526-2533.
29. Dreyfus M, Joyce S, editors (2003) The interplay between translation and mRNA decay in prokaryotes. Georgetown, TX: Landes Bioscience. 165–183 p.
30. Voulgaris J, French S, Gourse RL, Squires C, Squires CL (1999) Increased rrn gene dosage causes intermittent transcription of rRNA in Escherichia coli. Journal of Bacteriology 181: 4170-4175.
31. Churchward G, Bremer H, Young R (1982) Macromolecular-Composition of Bacteria. Journal of Theoretical Biology 94: 651-670.
32. Meyer BJ, Schottel JL (1991) A Novel Transcriptional Response by the Cat Gene During Slow Growth of Escherichia-Coli. Journal of Bacteriology 173: 3523-3530.
33. Nilsson G, Belasco JG, Cohen SN, Vongabain A (1984) Growth-Rate Dependent Regulation of Messenger-Rna Stability in Escherichia-Coli. Nature 312: 75-77.
34. Wagner LA, Gesteland RF, Dayhuff TJ, Weiss RB (1994) An Efficient Shine-Dalgarno Sequence but Not Translation Is Necessary for Lacz Messenger-Rna Stability in Escherichia-Coli. Journal of Bacteriology 176: 1683-1688.
35. Alon U (2006) An Introduction to Systems Biology. Boca Raton, FL: Chapman & Hall/Crc Mathematical and Computational Biology Series.
36. Steward KL, StPierre R, Linn T (1997) Transcription-frequency-dependent modulation of an attenuator in a ribosomal protein RNA polymerase operon requires an upstream site. Microbiology-Uk 143: 3501-3511.
37. Downing W, Dennis PP (1991) Rna-Polymerase Activity May Regulate Transcription Initiation and Attenuation in the Rplkajlrpobc Operon in Escherichia-Coli. Journal of Biological Chemistry 266: 1304-1311.

38. Dennis PP (1984) Site Specific Deletions of Regulatory Sequences in a Ribosomal Protein-Rna Polymerase Operon in Escherichia-Coli - Effects on Beta and Beta' Gene-Expression. Journal of Biological Chemistry 259: 3202-3209.
39. Dennis PP, Nene V, Glass RE (1985) Autogenous Posttranscriptional Regulation of Rna Polymerase-Beta and Beta' Subunit Synthesis in Escherichia-Coli. Journal of Bacteriology 161: 803-806.
40. Browning ST, Castellanos M, Shuler ML (2004) Robust control of initiation of prokaryotic chromosome replication: Essential considerations for a minimal cell. Biotechnology and Bioengineering 88: 575-584.
41. Hayward RS, Fyfe SK (1978) Non-Coordinate Expression of Neighboring Gene-Rpll and Gene-Rpob, Gene-C of Escherichia-Coli. Molecular & General Genetics 160: 77-80.
42. Dennis PP (1977) Regulation of Synthesis and Activity of a Mutant Rna-Polymerase in Escherichia-Coli. PNAS 74: 5416-5420.
43. Fukuda R, Nagasawafujimori H (1983) Mechanism of the Rifampicin Induction of Rna-Polymerase Beta-Subunit and Betas-Subunit Synthesis in Escherichia-Coli. Journal of Biological Chemistry 258: 2720-2728.
44. Cooper S, Helmstetter CE (1968) Chromosome Replication and Division Cycle of Escherichia Coli B/R. Journal of Molecular Biology 31: 519-540.
45. Passador L, Linn T (1989) Autogenous Regulation of the Rna-Polymerase Beta-Subunit of Escherichia-Coli Occurs at the Translational Level Invivo. Journal of Bacteriology 171: 6234-6242.
46. Donachie WD, Blakely GW (2003) Coupling the initiation of chromosome replication to cell size in Escherichia coli. Current Opinion in Microbiology 6: 146-150.
47. Bipatnath M, Dennis PP, Bremer H (1998) Initiation and velocity of chromosome replication in Escherichia coli B/r and K-12. Journal of Bacteriology 180: 265-273.
48. Donachie WD (1968) Relationship between Cell Size and Time of Initiation of DNA Replication. Nature 219: 1077-&.
49. Bremer H, Churchward G (1977) Examination of Cooper-Helmstetter Theory of DNA-Replication in Bacteria and Its Underlying Assumptions. Journal of Theoretical Biology 69: 645-654.
50. Bremer H, Churchward G, Young R (1979) Relation between Growth and Replication in Bacteria. Journal of Theoretical Biology 81: 533-545.
51. Speck C, Weigel C, Messer W (1999) ATP- and ADP-DnaA protein, a molecular switch in gene regulation. Embo Journal 18: 6169-6176.
52. Messer W (2002) The bacterial replication initiator DnaA. DnaA and oriC, the bacterial mode to initiate DNA replication. Fems Microbiology Reviews 26: 355-374.
53. Hansen FG, Atlung T, Braun RE, Wright A, Hughes P, et al. (1991) Initiator (Dnaa) Protein-Concentration as a Function of Growth-Rate in Escherichia-Coli and Salmonella-Typhimurium. Journal of Bacteriology 173: 5194-5199.
54. Tao H, Bausch C, Richmond C, Blattner FR, Conway T (1999) Functional genomics: Expression analysis of Escherichia coli growing on minimal and rich media. Journal of Bacteriology 181: 6425-6440.
55. Nierlich DP, Murakawa GJ (1996) The decay of bacterial messenger RNA. Progress in Nucleic Acid Research and Molecular Biology, Vol 52. pp. 153-216.
56. Lovmar M, Ehrenberg M (2006) Rate, accuracy and cost of ribosomes in bacterial cells. Biochimie 88: 951-961.
57. Minton AP (1998) Molecular crowding: Analysis of effects of high concentrations of inert cosolutes on biochemical equilibria and rates in terms of volume exclusion. Methods in Enzymology 295: 127-149.
58. Minton AP (1990) Holobiochemistry - the Effect of Local Environment Upon the Equilibria and Rates of Biochemical Reactions. International Journal of Biochemistry 22: 1063-1067.

59. Minton AP (1981) Excluded Volume as a Determinant of Macromolecular Structure and Reactivity. Biopolymers 20: 2093-2120.
60. Minton AP (2001) The influence of macromolecular crowding and macromolecular confinement on biochemical reactions in physiological media. Journal of Biological Chemistry 276: 10577-10580.
61. Ellis RJ (2001) Macromolecular crowding: an important but neglected aspect of the intracellular environment. Current Opinion in Structural Biology 11: 114-119.
62. Jarvis TC, Ring DM, Daube SS, von Hippel PH (1984) "Macromolecular Crowding": Thermodynamic Consequences for Protein-Protein Interactions within the T4 DNA Replication Complex. Journal of Biological Chemistry 265: 15160-15167
63. Banks DS, Fradin C (2005) Anomalous diffusion of proteins due to molecular crowding. Biophysical Journal 89: 2960-2971.
64. Golding I, Cox EC (2006) Physical nature of bacterial cytoplasm. Physical Review Letters 96.
65. Tokuriki N, Kinjo M, Negi S, Hoshino M, Goto Y, et al. (2004) Protein folding by the effects of macromolecular crowding. Protein Science 13: 125-133.
66. Maurizi MR (1992) Proteases and Protein-Degradation in Escherichia-Coli. Experientia 48: 178-201.
67. Minton AP (2000) Implications of macromolecular crowding for protein assembly. Current Opinion in Structural Biology 10: 34-39.
68. van den Berg B, Ellis RJ, Dobson CM (1999) Effects of macromolecular crowding on protein folding and aggregation. Embo Journal 18: 6927-6933.
69. Cayley S, Lewis BA, Guttman HJ, Record MT (1991) Characterization of the Cytoplasm of Escherichia-Coli-K-12 as a Function of External Osmolarity - Implications for Protein DNA Interactions Invivo. Journal of Molecular Biology 222: 281-300.
70. Record MT, Courtenay ES, Cayley DS, Guttman HJ (1998) Responses of E-coli to osmotic stress: Large changes in amounts of cytoplasmic solutes and water. Trends in Biochemical Sciences 23: 143-148.
71. Record MT, Courtenay ES, Cayley S, Guttman HJ (1998) Biophysical compensation mechanisms buffering E-coli protein-nucleic acid interactions against changing environments. Trends in Biochemical Sciences 23: 190-194.
72. Cayley S, Record MT (2004) Large changes in cytoplasmic biopolymer concentration with osmolality indicate that macromolecular crowding may regulate protein-DNA interactions and growth rate in osmotically stressed Escherichia coli K-12. Journal of Molecular Recognition 17: 488-496.
73. Wenner JR, Bloomfield VA (1999) Crowding effects on EcoRV kinetics and binding. Biophysical Journal 77: 3234-3241.
74. Minton AP (1983) The Effect of Volume Occupancy Upon the Thermodynamic Activity of Proteins - Some Biochemical Consequences. Molecular and Cellular Biochemistry 55: 119-140.
75. Zimmerman SB, Harrison B (1987) Macromolecular Crowding Increases Binding of DNA-Polymerase to DNA - an Adaptive Effect. PNAS 84: 1871-1875.
76. Harrison B, Zimmerman SB (1986) Stabilization of T4 Polynucleotide Kinase by Macromolecular Crowding. Nucleic Acids Research 14: 1863-1870.
77. Zimmerman SB, Trach SO (1988) Macromolecular Crowding Extends the Range of Conditions under Which DNA-Polymerase Is Functional. Biochimica Et Biophysica Acta 949: 297-304.
78. Phillies GDJ (1986) Universal Scaling Equation for Self-Diffusion by Macromolecules in Solution. Macromolecules 19: 2367-2376.
79. Dauty E, Verkman AS (2004) Molecular crowding reduces to a similar extent the diffusion of small solutes and macromolecules: measurement by fluorescence correlation spectroscopy. Journal of Molecular Recognition 17: 441-447.

80. Elowitz MB, Surette MG, Wolf PE, Stock JB, Leibler S (1999) Protein mobility in the cytoplasm of Escherichia coli. Journal of Bacteriology 181: 197-203.
81. Zimmerman SB, Minton AP (1993) Macromolecular Crowding - Biochemical, Biophysical, and Physiological Consequences. Annual Review of Biophysics and Biomolecular Structure 22: 27-65.
82. Berg OG (1990) The Influence of Macromolecular Crowding on Thermodynamic Activity - Solubility and Dimerization Constants for Spherical and Dumbbell-Shaped Molecules in a Hard-Sphere Mixture. Biopolymers 30: 1027-1037.
83. Guttman HJ, Anderson CF, Record MT (1995) Analyses of Thermodynamic Data for Concentrated Hemoglobin-Solutions Using Scaled Particle Theory - Implications for a Simple 2-State Model of Water in Thermodynamic Analyses of Crowding in-Vitro and in-Vivo. Biophysical Journal 68: 835-846.
84. Ellis RJ, Minton AP (2003) Cell biology - Join the crowd. Nature 425: 27-28.
85. Hall D, Minton AP (2003) Macromolecular crowding: qualitative and semiquantitative successes, quantitative challenges. Biochimica Et Biophysica Acta-Proteins and Proteomics 1649: 127-139.
86. Alon U (2005) Lecture notes.
87. Jiang XQ, Diwa A, Belasco JG (2000) Regions of RNase E important for 5 '-end-dependent RNA cleavage and autoregulated synthesis. Journal of Bacteriology 182: 2468-2475.
88. Beran RK, Prud'homme-Généreux A, Baker KE, Miao X, Simons RW, et al. (2003) mRNA decay in Escherichia coli: Enzymes, mechanisms and adaptation. In: Lapointe J, Brakier-Gingras L, editors. Translation mechanisms. 1 edition ed. Georgetown, TX: Landes Bioscience. pp. 148–164.
89. Jain C, Deana A, Belasco JG (2002) Consequences of RNase E scarcity in Escherichia coli. Molecular Microbiology 43: 1053-1064.
90. Kushner SR (2002) mRNA decay in Escherichia coli comes of age. Journal of Bacteriology 184: 4658-4665.
91. Jain C (2002) Degradation of mRNA in Escherichia coli. Iubmb Life 54: 315-321.
92. Deutscher MP (2006) Degradation of RNA in bacteria: comparison of mRNA and stable RNA. Nucleic Acids Research 34: 659-666.
93. Jiang XQ, Belasco JG (2004) Catalytic activation of multimeric RNase E and RNase G by 5 '-monophosphorylated RNA. PNAS 101: 9211-9216.
94. Redko Y, Tock MR, Adams CJ, Kaberdin VR, Grasby JA, et al. (2003) Determination of the catalytic parameters of the N-terminal half of Escherichia coli ribonuclease E and the identification of critical functional groups in RNA substrates. Journal of Biological Chemistry 278: 44001-44008.
95. Carrier TA, Keasling JD (1997) Mechanistic modeling of prokaryotic mRNA decay. Journal of Theoretical Biology 189: 195-209.
96. Sousa S, Marchand I, Dreyfus M (2001) Autoregulation allows Escherichia coli RNase E to adjust continuously its synthesis to that of its substrates. Molecular Microbiology 42: 867-878.
97. Jain C, Belasco JG (1995) Rnase-E Autoregulates Its Synthesis by Controlling the Degradation Rate of Its Own Messenger-Rna in Escherichia-Coli - Unusual Sensitivity of the Rne Transcript to Rnase-E Activity. Genes & Development 9: 84-96.
98. Regnier P, Arraiano CM (2000) Degradation of mRNA in bacteria: emergence of ubiquitous features. Bioessays 22: 235-244.
