# Supplementary material for: A Coarse-Grained Biophysical Model of E. coli and Its Application to Perturbation of the rRNA Operon Copy Number
Source: PLoS Comput Biol. 2008 May 2;4(5):e1000038. doi: 10.1371/journal.pcbi.1000038 (PMC2320978; doi:10.1371/journal.pcbi.1000038)
Supplement: Table S1 — Genetic parameters for E. coli growing at 1 and 2.5 doub/h, 37°C. (0.13 MB PDF) [file pcbi.1000038.s002.pdf]

Table S1. Genetic parameters for *E. coli* growing at 1 and 2.5 doub/h, 37°C. Values not in parentheses are for 2.5 doub/h. Values in parentheses are for 1 doub/h, with the rest of the values being identical to those at 2.5 doub/h. Note that all concentrations have been multiplied by the average cell volume $V_{cell}(\mu_0)$ of a WT cell growing in the given medium, i.e. μ_0=2.5 doub/h (and 1 doub/h). Cell volumes are given in Table S2. Using these units for $K_{m,i}$, $L_{m,i}$ and d_i when solving the equations of state for a genetically perturbed cell generates concentrations (e.g. $n_{RNAp,free}$, $n_{ribo,free}$ etc.) also in these units, i.e. concentration times $V_{cell}(\mu_0)$. Values in bold are estimates (see respective footnotes and main text).

Gene class		Units	r-protein[a]	bulk[b]	*rrn*[c]
m [d]	Map location	MU (min)	see footnote d	66 (417) uniformly distributed genes	C 84.5; D 72.1; G 56.1; H 5.1; B 89.8; E 90.5; A 86.5
$V_i^{\max}$ [e]	Maximum transcription initiation rate	ini/min	33	3.04 (1.71)	110
$U_i^{\max}$ [f]	Maximum translation initiation rate	ini/min	-	**80**	-
$K_{m,i}$ [g]	Promoter-RNAp holoenzyme binding affinity	molec/cell	501 (264)	501 (264)	797 (1440)
$L_{m,i}$ [h]	RBS-30S ribosome subunit binding affinity	molec/cell	-	**6144 (3226)**	-
$T_{1/2,i}^{fun}$ [i]	mRNA half-life	min	-	6.8 **(1.28)**	-
L_i [j]	Gene class length	base pairs	21252	1000	6623
c_p [k]	Peptide chain elongation rate	aa/sec	21 (16)	21 (16)	-
c_i [l]	RNA chain elongation rate	nuc/sec	55 (45)	1.37 (4.09)	85

[a] This class is analogous to the constitutive gene class of [28], where we consolidated all r-protein genes into a single operon (c.f. footnote d). Note that r-protein is assumed to be constitutive as transcription of most r-protein operons is not growth rate regulated [1].

[b] Bulk gene class gene dosage D_{bulk}, maximum transcription initiation rate, V^{max}_{bulk}, and transcription elongation rate, c_{bulk}, are a consolidation of repressable (r) and pause (ps) promoter classes of [28] such that the bulk gene class is mathematically equivalent to the sum of these two promoter classes in terms of transcription and translation (see below).

[c] *rrn* gene class is a consolidation of P1 and P2 *rrn* promoter classes in [28].

[d] To convert from MU (min) (0<MU<100 min) to units relative to oriC (0<m<1) see table 5 in [17]. *rrn* map locations were taken from [54]. Bulk protein genes are assumed to be uniformly distributed across the chromosome: $m = (0,1,\ldots, N_{bulk}-1)/N_{bulk}$. The number of bulk genes, N_{bulk}, was ascertained using Eq. S7 for the gene dosage: $D_{bulk}(\mu) = \sum_{i=0}^{N_{bulk}-1} 2^{\mu(C(1-m_i)+D)} = 2^{\mu(C+D)}\left(1-2^{-\mu C}\right)/\left(1-2^{-\mu C/N_{bulk}}\right)$, $m_i = i/N_{bulk}$, and solving for N_{bulk}, where $D_{bulk} = D_r + D_{ps}$ and D_r and D_{ps} are the gene dosage of repressable and pause promoter classes respectively, taken from tables 1 and 2 of [28]: D_{bulk}(2.5 doub/h) = 251 and D_{bulk}(1 doub/h) = 771. Gene concentrations $d_i(\mu)$ in Eq. 2 can be calculated using Eq. 3: $d_i(\mu) = \frac{1}{\ln 2 V_{ini}} \sum_j 2^{-m_j^{(i)} C\mu}$, where $(V_{ini}\ln 2)^{-1} = V_{cell}^{-1}(\mu_0) 2^{\mu_0(C+D)}$ (see Eq. S8) is evaluated for a high enough μ_0. For example, for μ_0 = 2.5 doub/h, $(V_{ini}\ln 2)^{-1} = 5.45(\mu m)^{-3}$ (see Table S2 for WT cell volumes). In practice, for genetic perturbation simulations it is convenient to use concentration in units of concentration$\cdot V_{cell}(\mu_0)$, where μ_0 is chosen to be the growth rate of a WT cell in the given environment. Thus $d_i(\mu)V_{cell}(\mu_0) = 2^{\mu_0(C+D)} \sum_j 2^{-m_j^{(i)} C\mu}$. The factor $2^{\mu_0(C+D)}$ is 6.54 for 2.5 doub/h and 2.43 for 1 doub/h (C and D are given in Table S2). The only exception to this rule is in the calculation of gene concentration for the r-protein gene class. Since the 19 operons of r-protein were concatenated on a *single* operon, in order to capture a more realistic growth rate dependence of the gene concentration, the following formula was used: $d_{r-protein}(\mu) = d_{r-protein}(\mu_0) \sum_{j=1}^{19} 2^{-m_j^{(r-protein)} C\mu} \Big/ \sum_{j=1}^{19} 2^{-m_j^{(r-protein)} C\mu_0}$ where $d_{r\text{-}protein}(\mu_0) = d_{rrn}(\mu_0)/7$, μ_0 = growth rate of WT cell in the given medium, $d_{rrn}(\mu_0) = D_{rrn}(\mu_0)/V_{cell}(\mu_0)$ and $D_{rrn}(\mu_0) = \sum_{j=1}^{7} 2^{\mu\left(C(1-m^{(rrn)})+D\right)}$. For example, $D_{rrn}(\mu_0$ = 2.5 doub/h) = 36.0 and $D_{rrn}(\mu_0$ = 1.0 doub/h) = 15.1 (also c.f. table 2 of [28] and table 3 of [17]); $D_{r\text{-}protein}(\mu_0$ = 2.5 doub/h) = 5.14 and $D_{r\text{-}protein}(\mu_0$ = 1.0 doub/h) = 2.16; r-protein map locations are: 74.27, 74.38, 74.15, 74.84, 90.02, 90.05, 71.33, 38.75, 0.45, 20.72, 59.14, 4.09, 95.33, 69.16, 72.77, 49.15, 82.11, 24.71, 83.68 min [1].

[e] Maximum transcription initiation rates for the r-protein and *rrn* gene classes are given by the constitutive and *rrn* promoter classes respectively in tables 1 and 2 of [28]. The maximum transcription initiation rate for the bulk gene class is given by

$V_{bulk}^{\max} = \left(D_r V_r^{\max} + D_{ps} V_{ps}^{\max}\right) / D_{bulk}$ where $V^{max}{}_r$ and $V^{max}{}_{ps}$ are the maximum transcription initiation rates of repressable and pause promoter classes respectively, taken from tables 1 and 2 of [28] and D_{bulk} is defined in footnote d.

[f] The maximum translation initiation rate for the bulk gene class, $U_{bulk}^{\max}$, was chosen to be an arbitrarily value above observed translation initiation rates and below the maximum physical limit set by close packing of ribosomes. See also S1.1.1 and main text.

[g] RNAp holoenzyme-promoter binding affinity for r-protein and for bulk gene classes are given by the constitutive and repressable/pause binding affinities given in tables 1 and 2 of [28]. The binding affinity for the *rrn* gene class, $K_{m,rrn}$, was calculated based on $i_{rrn} = V_{rrn}^{\max} n_{RNAp,free} / \left(n_{RNAp,free} + K_{m,rrn}\right)$, where $n_{RNAp,free}$ is the free RNAp concentration and i_{rrn} is the observed number of initiations per *rrn* operon, given in table 3 of [28]. K_m values are given in units of concentration times the volume of a WT cell in the given medium. To convert to μM: K_m(μM) = K_m(molec per cell)/V_{cell}((μm)^3)/602. V_{cell} is given in Table S2. Note that for constitutive promoters: K_m(1 doub/h; molec/cell) = K_m(2.5 doub/h; molec/cell)V_{cell}(1 doub/h)/V_{cell}(2.5 doub/h).

[h] Ribosome binding affinities were calculated as described in the main text and S1.1.1. To convert to μM use formula given in footnote g. It was assumed that bulk mRNA expression parameters, $U_{bulk}^{\max}$ and $L_{m,bulk}$, are fixed and growth rate independent and were based on estimation at 2.5 doub/h (c.f. S1.1.1). Therefore L_m(1 doub/h; molec/cell)= L_m(2.5 doub/h; molec/cell)V_{cell}(1 doub/h)/V_{cell}(2.5 doub/h).

[i] Since mRNA half-life can alter due to translation-degradation coupling (see main text and S2.7), the actual genetic parameter is not $T_{1/2,i}^{fun}$, but rather the mRNA half-life in the absence of ribosomes, $T_{1/2,i}^{fun,0}$. $T_{1/2,i}^{fun,0}$ is a combination of the Michaelis-Menten parameters of mRNA degradation ($W^{max}{}_i$, $J_{m,i}$) and the mRNA binding affinity of RNase E to its own mRNA, J_1 (c.f. S2.5.2 and S2.7). For mRNA decay via the 5' competition model (Eq. R3 in S2.5.2) this parameter is given by $T_{1/2,i}^{fun,0} = \left[J_{m,i} / \left(J_1 W_i^{\max}\right)\right] \ln 2$ and according to this model it can be calculated from $T_{1/2,i}^{fun,0} = T_{1/2,i}^{fun}(WT)\left(1 + n_{ribo,free}(WT)/L_{m,i}\right)^{-1}$, where $n_{ribo,free}$(WT) is the WT concentration of free ribosomes and $T_{1/2,i}^{fun}(WT)$ is the WT mRNA half-life of the *i*-th gene class (see S2.5.2, S2.6, S2.7 and main text for further details). Determination of $T_{1/2,i}^{fun}$: total intensity of all mRNA in *E. coli* MG1655 in LB broth at 37°C was measured to decay exponentially with a half-life of 6.8 min [40] (the exact growth rate of the culture was not given). This value was taken as the bulk mRNA half-life. This approximation is valid if r-protein half-life is close to the total mRNA half-life. To check this, using data published online (mRNA_half_lives.txt, [40] online), the average mRNA intensity weighed half-life of all genes was 8.6 min (where intensity was approximated by the average difference at t=0, 2-fold method; setting, as the authors, HL>40min to 40min [40]). The average intensity weighted half-life of all r-protein genes was 8.2 min, close to the average mRNA half-life of all genes. For 1 doub/h, $U_{bulk}^{\max}$ and $L_{m,bulk}$ were assumed to be fixed at their value at 2.5 doub/h, and $T_{1/2,i}^{fun}$ was estimated to minimize the error with respect to the wild-type cell state at 1 doub/h (see S1.1.1).

[j] For the r-protein gene class, $L_{r\text{-}protein}$ is taken to be the sum of all r-proteins gene lengths for MG1655. Genes are listed in [71] pp. 60-61. L7 is discarded since it is identical to L12 (only N-acetylated). L26 is discarded since it is identical to S20 and appears only once in the 70S ribosome [71] p. 61. Note that the number of amino acids in a 70S ribosome is slightly higher (=22332/3) since L7/L12 appears in four copies in the final ribosome [71] p. 61. Bulk gene class length is based on tables 1 and 2 of [28] (for comparison, according to [102] the average is 1100 bp). *rrn* gene length is calculated as follows: There are 4566 bp of rRNA in a 70S ribosome (table 1 in [17]); precursor rRNA length is 6000bp (table 1 in [17]); The length of the *rrn* gene class includes transfer RNA (tRNA) coding genes since rRNA and tRNA are stoichiometrically fixed at a ratio of 14:86 at all growth rates (table 1 of [17]; [3]). Although this fixed proportionally appears to be disrupted in perturbed cells [19], we currently do not take this correction into account. Since this gene class includes tRNA transcription and any overhead in the precursor rRNA, the total gene class length is: (total rRNA + total tRNA) + (precursor overhead) = 4566/0.86 + (6000 – 120 – 4566) = 6623bp where 120bp is the average tRNA gene length inside *rrn* operons (which is subtracted because it is already included in the 14% tRNA).

[k] See table 3 in [17]. By redefining c_p for the *i*-th gene class as: $c_{p,i} = c_p / \left(1 + c_p / \left(L_i U_i^{\max}\right)\right)$ one can take into account ribosomes bound to the RBS of the *i*-th gene class mRNA, which was neglected in Eq. 2v. For $U^{max}{}_i$ = 80 ini/min, for example, this correction is quite small (21→20 aa/sec and 16→15.4 aa/sec) but it may be consequential for low values of $U^{max}{}_i$. This correction was included in the simulations for the bulk protein gene class.

[l] r-protein mRNA chain elongation rate and rRNA chain elongation rate are given by the constitutive promoter class mRNA elongation rate and *rrn* promoter class rRNA chain elongation rate respectively in tables 1 and 2 in [28]. Bulk gene class mRNA chain elongation rate is given by $c_{bulk} = D_{bulk} V_{bulk}^{\max} \left(D_r V_r^{\max} / c_r + D_{ps} V_{ps}^{\max} / c_{ps}\right)^{-1}$ where D_{bulk} is defined in footnote d and $V^{max}{}_{bulk}$, $V^{max}{}_r$ and $V^{max}{}_{ps}$ are defined in footnote e and c_r and c_{ps} are the transcription elongation rate for repressable and pause genes respectively defined in tables 1 and 2 of [28]. By redefining c_i to be $c_i' = c_i / \left(1 + c_i / \left(L_i V_i^{\max}\right)\right)$ one can take into account RNAp bound to promoters, which was neglected in Eq. 2iv. This correction is substantial for repressable genes due to their low V^{max}. In the simulations this correction was implemented for the pause and repressable gene classes (c_r and c_{ps}). By correcting c_r and c_{ps} one obtains c_{bulk}=1.36 (3.58) nuc/sec for 2.5 (1.0) doub/h. We note that mRNA chain elongation rate in table 2 of [28] should be 55 nuc/sec and not 45 nuc/sec, due to a misprint.
