# Supplementary material for: A Coarse-Grained Biophysical Model of E. coli and Its Application to Perturbation of the rRNA Operon Copy Number
Source: PLoS Comput Biol. 2008 May 2;4(5):e1000038. doi: 10.1371/journal.pcbi.1000038 (PMC2320978; doi:10.1371/journal.pcbi.1000038)
Supplement: Table S2 — Cell state and additional parameters for various growth conditions. (0.12 MB PDF) [file pcbi.1000038.s003.pdf]

Table S2. Cell state and additional parameters (in gray) for various growth conditions. Measurements are for *E. coli* at 37°C according to data from [28] table 4 and from [17] tables 2 and 3. Number of molecules per cell are measured as average number per cell, i.e. number per unit of volume of culture divided by the number of cells in that culture, as defined by [17]. To convert to µM: $n(\mu\text{M}) \cong N(\text{molec/cell}) \big/ \left[602 \cdot V_{cell} [\mu m]^3\right]$. Mass and volume of bulk proteins and ribosomes: Bulk protein mass is calculated assuming 108 D/aa (Dalton per amino acid; table 2 in [17]), m_{bulk}=1000 bp/3*108=36 kD. Ribosome mass is calculated to include tRNA, and assuming 324 D/RNA nuc (table 2 in [17]), and with 4566 RNA nuc per 70S ribosome (table 1 in [17]). Thus: m_{ribo}=22332/3*108+4566/0.86*324=2.52MD (where 22332/3 is the number of amino acids present in a 70S ribosome- see Table S1 footnote j). Assuming a macromolecule specific density of 1 ml/g [46] we find that the specific volumes of bulk proteins and ribosomes (including tRNA overhead) are v_{bulk}=5.98 *10^{-8} $(\mu m)^3$ and v_{ribo}= 4.18*10^{-6} $(\mu m)^3$ respectively.

State variable	Meaning	Units			
μ	Growth rate	doub/h	2.5	2.0	1.0
N_{bulk} [a]	Average number of bulk proteins per cell	molec/cell	$5.76 \cdot 10^6$	$4.57 \cdot 10^6$	$2.29 \cdot 10^6$
N_{RNAp} [c]	Average total number of RNAp core enzymes (mature and immature) per cell	molec/cell	11400	8000	2800
$N_{RNAp,free}$ [b]	Average number of free functional RNAp core enzymes per cell	molec/cell	886	389	144
N_{ribo} [c]	Average total number of ribosomes per cell (mature and immature)	molec/cell	72000	45100	13500
$N_{ribo,free}$ [d]	Average number of free functional ribosomes per cell	molec/cell	4719	4100	1942
V_{cell} [e]	Cell volume	$(\mu m)^3$	1.2	0.97	0.63
C [c]	C period	min	42	43	50
D [c]	D period	min	23	24	27

[a] $< N_{bulk} >= (1-\alpha_r-\alpha_p)3\text{P}_c / L_{bulk}$, where P_c is the amount of protein per cell (in amino acid residues) (table 2 in [17]), α_r is the percent of r-protein per total protein (table 3 in [17]), α_p is the percent of RNAp per total protein, (table 3 in [17]) and L_{bulk} is the length of the bulk protein gene class (in base pairs), given in Table S1.

[b] Data for 1 and 2.5 doub/h is taken from [28]. For 2 doub/h see calculation in S1.2. Note that the σ^{70} subunit is present at 20% to 40% of the level of core polymerase [29,74] ($\beta\beta'\alpha_2\sigma^{70}$ is the major form of holoenzyme in exponential growth responsible for transcription initiation of all rRNA, tRNA and most mRNA operons [29]) but it is most likely in excess over free core, and thus newly formed core is expected to be rapidly converted into holoenzyme [74]. This can be seen from the following calculation (also c.f. [74]): by measurement of the β and β' subunit concentration in minicells (minicells are DNA-less portions of bacterial cytoplasm pinched off during aberrant cell divisions in certain bacterial mutants [28]) it has been estimated that the concentration of cytoplasmic β and β' subunits of RNAp is about 17% of the value in whole cells [74]. At 2.5 doub/h the percentage of immature RNAp is about $\left(1-e^{-\alpha\tau_{RNAp}}\right)=8.3\%$ (see Eq. S13) leaving 17%-8.3%=8.7%=991 free core enzymes per cell at this growth rate (also see [28]; the amount of core enzyme is limited by the concentration of β and β' since α subunit is in excess [17]). Since σ^{70} is released prior to the start of RNA chain elongation, the number of σ^{70} bound to promoters has an upper limit which is the number of RNAp molecules bound to the promoter. For 2.5 doub/h this number is estimated to be 216 RNAp molecules (table 2 in [28]). Therefore the number of free σ^{70} units is (20% to 40%)*11400 – 216 = 2064 to 4344 molec/cell, in excess of the number of free core polymerase = 991 molec/cell.

[c] See table 3 in [17]

[d] $N_{ribo,free} = N_{ribo} - N_{ribo,bound} - N_{ribo,imm} = (2^{-\mu\tau_{ribo}} - \beta_r)N_{ribo}$, where β_r is the fraction of active ribosomes (table 3 in [17]), $N_{ribo,bound}$ is the number of ribosomes per cell that are bound to mRNA, τ_{ribo} is the average assembly time of a ribosome (~5 min [17]) and $N_{ribo,imm}$ is the number immature ribosomes per cell (c.f. S2.5.1). Free ribosome concentration at 2.5, 2 and 1 doub/h correspond to 6.5, 7, 5.1 μM respectively. [90] estimated (neglecting nonfunctional ribosome units) an upper bound of 8.5 μM. Also as a comparison, in glucose-amino acid medium, it has been estimated that there are approximately 750 free ribosomes [32]. Note that a free 30S subunit can associate with a free 50S subunit to form a 70S subunit, and only free 30S subunits can initiate translation [71] pp. 203-204. Therefore free 70S ribosomes can in principle sequester free 30S units. However such sequestering is limited by the initiation factor IF3. IF3, binding of which – along with IF1 and IF2 – is a perquisite for subsequent attachment of the 30S subunit to mRNA and fMet-tRNA$_f$, prevents association of the 30S-IF3 complex with the free 50S subunit [71] p. 203, 206. It has been proposed that IF3 (in fact all three factors) are present at high enough concentration to saturate the free 30S subunits [103]. To see how this may be so we note the following: At 2.5 doub/h there is approximately one IF3 molecule per five ribosomes (and this ratio is more or less growth rate independent [15]). At 2.5 doub/h this amounts to 72000/5=14400 IF3 molec/cell. From this we should subtract the number of IF3 molecules bound to mRNA. The number of IF3 molecules bound to mRNA can be estimated as follows: If p is the probability that an mRNA RBS is occupied, then the number of RBSs that engage 30S subunits would be $p \cdot M_{tot}$, where M_{tot} is the total number of mRNA molecules per cell. Since a ribosome occupies the RBS for a time U^{-1}, where U is the average translation initiation rate of total mRNA, and since an IF3 molecule is ejected before peptide chain elongation commences [71] after some average time $T<U^{-1}$, then the average number of IF3 molecules bound at any given time to any mRNA would be $N_{IF3,bound}$,$\sim U \cdot T \cdot p \cdot M_{tot}$, and where U(2.5 doub/h) = 45 ini/min ([1] figure 3d), and $T \sim 30^{-1}$ sec ([71] p.208). $M_{tot} = T_{1/2}^{chem} / \ln 2 \cdot r_{mRNA}$ where r_{mRNA} is the mRNA synthesis rate. At 2.5 doub/h r_{mRNA}=*23.4·10*^{5} *nucl/min/cell/1000bp=39 mRNA/sec/cell* [17] table 3 (assuming 1000 bp per mRNA). For $T_{1/2}^{chem} \approx 6.8$ min (Table S1), $M_{tot} \approx 23000$ molec/cell and for p~0.5 we have $N_{IF3,bound} \sim 300$ per cell. Therefore the number of free IF3 molecules is ~14400-300=14100 per cell > free 30S subunits per cell ≈4700. This noted, it has also been estimated that *in vivo* there are about 8% 70S single (free) ribosomes present [104].

[e] Data for 1 and 2.5 doub/h is taken from [28]. For 2 doub/h volume was interpolated based on the method proposed in [28]: V_{cell}(2 doub/h)=V_M(2 doub/h)*M_C(2 doub/h) where V_M is volume per mass and M_C is mass per cell (in OD_{460} per cell) from [17] table 2. V_M , which changes only moderately with growth rate, was linearly interpolated for 2 doub/h given that V_M(μ=1.33 doub/h)= 0.32 mm^3 per OD_{460} and V_M(μ=2.14 doub/h)=0.25 mm^3 per OD_{460} [28].
