# Supplementary material for: A Coarse-Grained Biophysical Model of E. coli and Its Application to Perturbation of the rRNA Operon Copy Number
Source: PLoS Comput Biol. 2008 May 2;4(5):e1000038. doi: 10.1371/journal.pcbi.1000038 (PMC2320978; doi:10.1371/journal.pcbi.1000038)
Supplement: Table S3 — Reconstruction of the WT cell state for 1 and 2.5 doub/h, 37°C. (0.09 MB PDF) [file pcbi.1000038.s004.pdf]

Table S3. Reconstruction of the WT cell state for 1 and 2.5 doub/h, 37°C. Variables denoted in bold were allowed to vary in order to minimize the MSE with respect to the WT cell state. The error is cell state was not greater than 6%. All concentrations are given in molec/cell, where V_{cell} is 1.2 and 0.63 (μm)^3 at 2.5 and 1 doub/h respectively (Table S2). n_0=2.77 mM. The nonlinear least squares problem was solved using the Levenberg-Marquardt method implemented in Matlab 7. See main text and S1.1.1 in Text S1 for further explanations.

U_{bulk}^{max} (ini/min)	$L_{m,bulk}$ ($\cdot 10^2$)	c_{ribo}	$T_{1/2,bulk}^{fun}$ (min)	U_{bulk} (ini/min)	μ doub/h	n_{bulk} ($\cdot 10^6$)	n_{ribo} ($\cdot 10^4$)	n_{RNAp} ($\cdot 10^3$)	$n_{RNAP,free}$	$n_{ribo,free}$ ($\cdot 10^3$)
40	**9.57 (1.3 μM)**	**49**	6.8	33	2.5	**5.60**	**7.33**	11.4	**916**	**4.72**
80	**61.4 (8.5 μM)**	**53**	6.8	35	2.5	**5.85**	**7.33**	114	**916**	**4.72**
40	5.03 (1.3 μM)	**85**	**1.2**	32	1	**2.15**	**1.30**	2.8	**143**	**1.94**
80	32.3 (8.5 μM)	**90**	**1.3**	30	1	**2.23**	**1.30**	2.8	**143**	**1.94**
