# Supplementary material for: A Coarse-Grained Biophysical Model of E. coli and Its Application to Perturbation of the rRNA Operon Copy Number
Source: PLoS Comput Biol. 2008 May 2;4(5):e1000038. doi: 10.1371/journal.pcbi.1000038 (PMC2320978; doi:10.1371/journal.pcbi.1000038)
Supplement: Table S4 — Transcription related parameters for 2 doub/h, 37°C. (0.07 MB PDF) [file pcbi.1000038.s005.pdf]

Table S4. Transcription related parameters for 2 doub/h, 37°C. Transcription related parameters as defined in [28], obtained here for 2 doub/h. Values in bold were estimated. See S1.2 in Text S1 for further explanations.

Genetic parameter	Units	P1 [a]	P2 [a]	constitutive [a]	repressable [a]	pause [a]
$D_i = V_{cell}(2\text{ doub/h}) \cdot d_i$	average copies per cell	27 [b]	27 [b]	40.4 [c]	**408.4** [d]	161.8 [e]
$V_i^{\max}$	ini/min	110 [f]	110 [f]	33 [g]	1.5 [h]	3.3 [i]
c_i	nuc/sec	85 [j]	85 [j]	52 [j]	52 [j]	**0.89** [d]
$K_{m,i}$	molec/cell	1240 [l]	2531 [k]	405 [k]	405 [k]	405 [k]
L_i	base pairs	6000 [m]	6000 [m]	2000 [m]	1000 [m]	1000 [m]

[a] Promoter classes as defined by [28].
[b] The average number of copies of the *rrn* operon per cell, $D_{rrn}(\mu)$, is given by Eq. S7: $D_{rrn}(\mu) = \sum_{j=1}^{7} 2^{\mu\left(C(1-m_j^{(rrn)})+D\right)}$, where $m_j^{(rrn)}$ are the *rrn* operon map locations given in Table S1.
[c] According to [28], $[P_{constitutive}]=1.5[P_{rrn}]$, in order to fit to (1) transcription of all r-proteins and (2) mRNA synthesis rate. This is consistent with the length of the r-protein gene class given in Table S1: the total DNA per chromosome associated with this gene class is roughly 40.4/(27/7=gene dosage per gene)*(2000 bp) = 20,948 = total length of constitutive class coding genes, compared with $L_{r\text{-}protein}$= 21252 (Table S1).
[d] Estimated value - see S1.2.
[e] According to [28], based on known fractions of intermittently inactive RNAp in the cell $[P_{pause}] = 6[P_{rrn}]$.
[f] Estimated in [3]. Assumed to be growth rate independent [28].
[g] As measured for the spc ribosomal promoter, which is a representative promoter for this class (see [28]).
[h] As measured for β-lactamase promoter, which is taken to be a representative promoter for this class (see [28]).
[i] Maximum initiation rate was set to be ten fold lower than the V^{max} for the constitutive gene class, assuming pause genes are blocked 90% of the time [28].
[j] See table 3 in [17].
[k] Constitutive promoter binding affinities scale according to cell volume with respect to their values at 2.5 doub/h and are taken from [28]. Volumes are given in Table S2.
[l] Taken from table 5 of [105]: $K_{P1}:K_{P2}$ at (2.14 doub/h) = 0.49:1, i.e. 1240=0.49*2531.
[m] See tables 1 and 2 in [28].
