# Supplementary material for: A Coarse-Grained Biophysical Model of E. coli and Its Application to Perturbation of the rRNA Operon Copy Number
Source: PLoS Comput Biol. 2008 May 2;4(5):e1000038. doi: 10.1371/journal.pcbi.1000038 (PMC2320978; doi:10.1371/journal.pcbi.1000038)
Supplement: Table S5 — Genetic parameters for E. coli growing at 2 doub/h, 37°C. (0.08 MB PDF) [file pcbi.1000038.s006.pdf]

Table S5. Genetic parameters for *E. coli* growing at 2 doub/h, 37°C. See also footnotes in Table S1 and S1.2 in Text S1 for further explanations.

Gene class		Units	r-protein	bulk	*rrn*
m [h]	Map location	MU (min)	see footnote	191 uniformly distributed genes	see footnote
V_i^{max}	Maximum transcription initiation rate	ini/min	33 [a]	2.01 [d]	110 [a]
U_i^{max}	Maximum translation initiation rate	ini/min	-	80 [a]	-
$K_{m,i}$	Promoter-RNAp holoenzyme binding affinity	molec/cell	405 [c]	405 [c]	708 [i]
$L_{m,i}$	RBS-30S ribosome subunit binding affinity	molec/cell	-	13261 [g]	-
$T_{1/2,i}^{fun}$	mRNA half-life	min	-	6.8 [e]	-
L_i	Gene class length	base pairs	21252 [a]	1000 [a]	6623 [a]
c_p	Peptide chain elongation rate	aa/sec	20 [b]	20 [b]	-
c_i	RNA chain elongation rate	nuc/sec	52 [b]	1.87 [f]	85 [b]

[a] See footnote e Table S1. r-protein and *rrn* maximum transcription initiation rates are given in Table S4.

[b] See table 3 in [17]. By redefining c_p to include 30S subunits bound to the RBS we obtain 20→19.1 aa/sec. Also see footnote k in Table S1.

[c] K_m(2 doub/h; molec/cell) = K_m(2.5 doub/h; molec/cell)V_{cell}(2 doub/h)/V_{cell}(2.5 doub/h), where values for K_m and V_{cell} are taken from Tables S1 and S2 respectively.

[d] V^{max} for the bulk promoter is calculated according to data from Table S4- see footnote e in Table S1 for formula.

[e] Based on total mRNA half-life measurement for LB broth at 37°C [40] (see Table S1 footnote i).

[f] Bulk mRNA chain elongation rate, c_{bulk}, was calculated according to data from Table S4- see footnote l in Table S1 for formula. By redefining c_r and c_{ps} to include RNAp bound to the promoter we obtain c_{bulk}=1.78 nuc/sec. See footnote l, Table S1.

[g] 30S ribosome subunit binding affinity was estimated by finding the c_{ribo} and $L_{m,bulk}$ that minimize the mean square error between the predicted and observed WT cell state at 2 doub/h (Table S2), given $n_0 = 2.80*10^6$ molec/WT cell (see S1.1.1 for example at 2.5 doub/h). The estimated cost was $c_{ribo} \approx 38$ bulk protein per ribosome. n_0 was chosen so that the predicted cost is the cost that gives the best fit for the data of Asai et al.. See main text for further explanations regarding c_{ribo}.

[h] r-protein and *rrn* map locations are given in Table S1. The number of bulk genes was calculated as explained in footnote d of Table S1 (with D_r and D_{ps} for the calculation of D_{bulk} given in Table S4). Gene concentrations are calculated according to the formulae given in Table S1 footnote d with μ_0=2.0 doub/h, D_{rrn} (2 doub/h) = 27 copies per cell (Table S4), $D_{r\text{-}protein}$(2 doub/h) = 27/7 copies per cell (c.f. Table S4) and D_{bulk}(2 doub/h) ≅ 571 copies per cell.

[i] The binding affinity for the *rrn* gene class, $K_{m,rrn}$, was calculated as explained in Table S1 footnote g, where free RNAp concentration, $n_{RNAp,free}$, is given in Table S2 and i_{rrn}, the number of initiations per *rrn* operon for 2 doub/h, is given in table 3 of [28].
