# Supplementary material for: A Coarse-Grained Biophysical Model of E. coli and Its Application to Perturbation of the rRNA Operon Copy Number
Source: PLoS Comput Biol. 2008 May 2;4(5):e1000038. doi: 10.1371/journal.pcbi.1000038 (PMC2320978; doi:10.1371/journal.pcbi.1000038)
Supplement: Table S6 — Lineage of the rrn inactivation strains. (0.09 MB PDF) [file pcbi.1000038.s007.pdf]

Table S6. Lineage of the *rrn* inactivation strains. The following table traces the lineage of all *rrn* inactivation strains and indicates the length of each inactivated *rrn* operon that was transcribed, and in cases where the inactivated operon expressed a protein (*cat* or *lacZ* insertions), the length of the translated portion is also specified. A *Δn* strain indicates that *n* operons were inactivated. See S1.3 in Text S1 for further details.

Δ0 = *rrn*$^+$ =TA563	source: ΔTX11 from [106]

Δ1 = ΔAc = TA566	
**A transcription**: **2147bp** (with P_1P_2-CAT fusion) [1]. **translation**: cat$^+$: **657bp**	source : P_1P_2-CAT fusion BAG1 in [54][2]

Δ2 = ΔEAc = TA567	
**E** Completely deleted	source TXΔ11 [106]. See also fig. 1 in [91]
**A transcription**: **2147bp** (with P_1P_2-CAT fusion; see remarks in Δ1) . **translation**: cat$^+$: **657bp**	source : P_1P_2-CAT fusion BAG1 in [54]

Δ3 = ΔEBAc = TA568	
**E** Completely deleted	source TXΔ11 [106]. See also fig. 1 in [91]
**B transcription**: 2287(PCR:16S-23S)+119(5S) [3] =**2406 bp** [4]	source: pMA101, SalI-SalI deletion [7]
**A transcription**: **2147bp** (with P_1P_2-CAT fusion; see remarks in Δ1) . **translation**: cat$^+$: **657bp**	source : P_1P_2-CAT fusion BAG1 in [54]

Δ4 = ΔEBHAc = TA430	
**E** Completely deleted	source TXΔ11 [106]. See also fig. 1 in [91]
**B transcription**: 2287(PCR:16S-23S)+119(5S)=**2406 bp** (see remarks in Δ3)	source: pMA101, SalI-SalI deletion [7]
**H transcription**: 1290(PCR:16S-23S) + 248(5S+distal tRNA) = **1538 bp** [5]	source: pMA103, SacII-SacII deletion [7]
**A transcription**: **2147bp** (with P_1P_2-CAT fusion; see remarks in Δ1) . **translation**: cat$^+$: **657bp**	source : P_1P_2-CAT fusion BAG1 in [54]

Δ5 = ΔEBHGzAc = TA476	
**E** Completely deleted	source TXΔ11 [106]. See also fig. 1 in [91]
**B transcription**: 2287(PCR:16S-23S)+119(5S)=**2406 bp** (see remarks in Δ3)	source: pMA101, SalI-SalI deletion [7]
**H transcription**: 1290(PCR:16S-23S) + 248(5S+distal tRNA) = **1538 bp** (see remark in Δ4)	source: pMA103, SacII-SacII deletion [7]
**G transcription**: 2928(16S-23S) + 3075(*lacZ*$^+$)+212(5S) = **6215** [6]. **translation**: *lacZ*$^+$ **3075**	source pNY30, smaI-HpaI deletion in [7]
**A transcription**: **2147bp** (with P_1P_2-CAT fusion; see remarks in Δ1) . **translation**: cat$^+$: **657bp**	source : P_1P_2-CAT fusion BAG1 in [54]

Δ6 = ΔEBHGzADc = TA516	
**E** Completely deleted	source TXΔ11 [106]. See also fig. 1 in [91]
**B transcription**: 2287(PCR:16S-23S)+119(5S)=**2406 bp** (see remarks in Δ3)	source: pMA101, SalI-SalI deletion [7]
**H transcription**: 1290(PCR:16S-23S) + 248(5S+distal tRNA) = **1538 bp** (see remark in Δ4)	source: pMA103, SacII-SacII deletion [7]
**G transcription**: 2928 (16S-23S) + 3075(*lacZ*$^+$)+212(5S) = **6215** (see remark in Δ5) . **translation**: *lacZ*$^+$ **3075**	source pNY30, smaI-HpaI deletion in [7]
**A transcription**: 1290 (*rrnH* PCR:16S-23S) + 213(5S) = **1503bp**[7]	source: pNY34, SacII-SacII deletion [7]
**D transcription**: **2147bp** (with P_1P_2-CAT fusion) . **translation**: cat$^+$: **657bp**	source: W1485ΔD in [19][8]

---

[1] Measured from figure 1 in [54].

[2] Linage is traced as follows:

(i) BAG1 from [54] is designated CC164 in [19] and the A was inserted into the final strain.

(ii) A originated from the A1 strain of [54].

(iii) A1 was taken from [7] where it was designated W1485ΔA. W1485ΔA was formed by cutting *rrnA* at the first smaI restriction enzyme site (see figure 1 in [19]) and attaching to it a promoterless CAT gene from plasmid pKK232-8 [107]. pKK232-8 was also cut at smaI ([7] p. 4184, [107] p. 157). This is the first smaI site (see figure 1 in [19]) since according to [54] the CAT was inserted at position 612 bp from the start of 16S. According to figure 1 of [19] the first smaI is located at 605 bp position (assuming WT 16S-23S is 4719 bp [19]). This confirms that the scaling for this figure is correct.

(iv) pKK232-8 was designed by [107] and they used a CAT cassette based on [108].

(v) In [108] it is explained that CAT are derivatives of the Cm^r gene of the bacterial transposon Tn9 and contains the complete CAT polypeptide coding sequence. According to [108] this is 660 bp. This is confirmed in NCBI site for pKK232-8 (=657bp). Next we need to ascertain the length of the transcribed segment. The actual length of the CAT cassette in pKK232-8 is longer then 660 bp since (a) the coding region actually taken in [108] was 780bp and (b) pHH232-8 was cut at smaI which is upstream of the CAT cassette (figure 7 in [107]). Since the exact location of smaI was uncertain, the length of the transcribed gene was estimated from figure 1 in [54].

[3] Measured from end of 23S

[4] PCR length from [19] + 5S length taken from the NCBI site**.**

[5] PCR length from [19] + 5S+tRNA length taken from the NCBI site.

[6] Assuming a length of 4876 bp for the 16S-23S segment of *rrnG* (NCBI site) and from scaling of figure 1 in [19]. The first smaI was chosen, as described in figure 1 of [91]. LacZ length is from EcoCyc site, 5S length is from the NCBI site.

[7] Assumed to be like *rrnH* PCR fragment since the 16S-23S sequence length of *rrnA* and *rrnH* are both 4892 bp and the same restriction enzymes were used; 5S length was taken from the NCBI site.

[8] W1485ΔD in [19] was obtained by the same procedure as W1485ΔA described above: a promotorless CAT gene was inserted into the first smaI site at 612 bp; (see Δ1).
