# Supplementary material for: A Coarse-Grained Biophysical Model of E. coli and Its Application to Perturbation of the rRNA Operon Copy Number
Source: PLoS Comput Biol. 2008 May 2;4(5):e1000038. doi: 10.1371/journal.pcbi.1000038 (PMC2320978; doi:10.1371/journal.pcbi.1000038)
Supplement: Table S7 — Genetic parameters for the rrn inactivation strains at 2 doub/h, 37°C. (0.17 MB PDF) [file pcbi.1000038.s008.pdf]

Table S7. **Genetic parameters for the *rrn* inactivation strains at 2 doub/h, 37°C.** Strain names are indicated in bold. A *Δn* strain indicates that *n rrn* operons were inactivated. The table for the Δ0 (i.e. wild-type) strain is simply Table S5. Changes with respect to the previous strain are denoted in gray. See S1.3 in Text S1 for further details.

Δ0 = ΔTX11

**Gene class**		**Units**	**r-protein**	**bulk**	***rrn***
m	Map location	MU (min)	see Table S5		C(84.5), D(72.1), G(56.1), H(5.1), B(89.8), E(90.5), A(86.5)
V_i^{max}	Maximum transcription initiation rate	ini/min	33	2.01	110
U_i^{max}	Maximum translation initiation rate	ini/min	-	80	-
$K_{m,i}$	Promoter-RNAp holoenzyme binding affinity	molec/cell	405	405	708
$L_{m,i}$	RBS-30S ribosome subunit binding affinity	molec/cell	-	13261	-
$T_{1/2,i}^{fun}$	mRNA half-life	min	-	6.8	-
L_i transcr.	Gene class length transcribed	base pairs	21252	1000	6623
L_i transl.	Gene class length translated	base pairs	21252	1000	0
c_p	Peptide chain elongation rate	aa/sec	20	20	-
c_i	RNA chain elongation rate	nuc/sec	52	1.87	85

Δ1 = ΔAc = TA566

**Gene class**	***rrn***	**Load 1**
m	C(84.5), D(72.1), G(56.1), H(5.1), B(89.8), E(90.5)	A(86.5)
V_i^{max}	110	110
U_i^{max}	-	80
$K_{m,i}$	708	708
$L_{m,i}$	-	13261
$T_{1/2,i}^{fun}$	-	1.75
L_i transcr.	6623	2147
L_i transl.	0	657
c_p	-	20
c_i	85	85

Δ2 = ΔEAc = TA567

Gene class	*rrn*	Load 1	Load 2
m	C(84.5), D(72.1), G(56.1), H(5.1), B(89.8)	A(86.5)	E(90.5)
V_i^{max}	110	110	110
U_i^{max}	-	80	-
$K_{m,i}$	708	708	708
$L_{m,i}$	-	13261	-
$T_{1/2,i}^{fun}$	-	1.75	-
L_i transcr.	6623	2147	0
L_i transl.	0	657	0
c_p	-	20	-
c_i	85	85	85

Δ3 = ΔEBAc = TA568

Gene class	*rrn*	Load 1	Load 2	Load 3
m	C(84.5), D(72.1), G(56.1), H(5.1)	A(86.5)	E(90.5)	B(89.8)
V_i^{max}	110	110	110	110
U_i^{max}	-	80	-	-
$K_{m,i}$	708	708	708	708
$L_{m,i}$	-	13261	-	-
$T_{1/2,i}^{fun}$	-	1.75	-	-
L_i transcr.	6623	2147	0	2406
L_i transl.	0	657	0	0
c_p	-	20	-	-
c_i	85	85	85	85

Δ4 = ΔEBHAc = TA430

Gene class	*rrn*	Load 1	Load 2	Load 3	Load 4
m	C(84.5), D(72.1), G(56.1)	A(86.5)	E(90.5)	B(89.8)	H(5.1)
V_i^{max}	110	110	110	110	110
U_i^{max}	-	80	-	-	-
$K_{m,i}$	708	708	708	708	708
$L_{m,i}$	-	13261	-	-	-
$T_{1/2,i}^{fun}$	-	1.75	-	-	-
L_i transcr.	6623	2147	0	2406	1538
L_i transl.	0	657	0	0	0
c_p	-	20	-	-	-
c_i	85	85	85	85	85

Gene class	Δ5 = ΔEBHGzAc = TA476	*rrn*	Load 1	Load 2	Load 3	Load 4	Load 5
m		C(84.5), D(72.1)	A(86.5)	E(90.5)	B(89.8)	H(5.1)	G(56.1)
$V_i^{\max}$		110	110	110	110	110	110
$U_i^{\max}$		-	80	-	-	-	80
$K_{m,i}$		708	708	708	708	708	708
$L_{m,i}$		-	13261	-	-	-	13393
$T_{1/2,i}^{fun}$		-	1.75	-	-	-	1.5
L_i transcr.		6623	2147	0	2406	1538	6215
L_i transl.		0	657	0	0	0	3075
c_p		-	20	-	-	-	20
c_i		85	85	85	85	85	85

Gene class	Δ6 = ΔEBHGzADc = TA516	*rrn*	Load 1	Load 2	Load 3	Load 4	Load 5	Load 6
m		C(84.5)	A(86.5)	E(90.5)	B(89.8)	H(5.1)	G(56.1)	D(72.1)
$V_i^{\max}$		110	110	110	110	110	110	110
$U_i^{\max}$		-	-	-	-	-	80	80
$K_{m,i}$		708	708	708	708	708	708	708
$L_{m,i}$		-	-	-	-	-	13393	13261
$T_{1/2,i}^{fun}$		-	-	-	-	-	1.5	1.75
L_i transcr.		6623	1503	0	2406	1538	6215	2147
L_i transl.		0	0	0	0	0	3075	657
c_p		-	-	-	-	-	20	20
c_i		85	85	85	85	85	85	85
