# Supplementary figures and images for: A Coarse-Grained Biophysical Model of E. coli and Its Application to Perturbation of the rRNA Operon Copy Number
Source: PLoS Comput Biol. 2008 May 2;4(5):e1000038. doi: 10.1371/journal.pcbi.1000038 (PMC2320978; doi:10.1371/journal.pcbi.1000038)

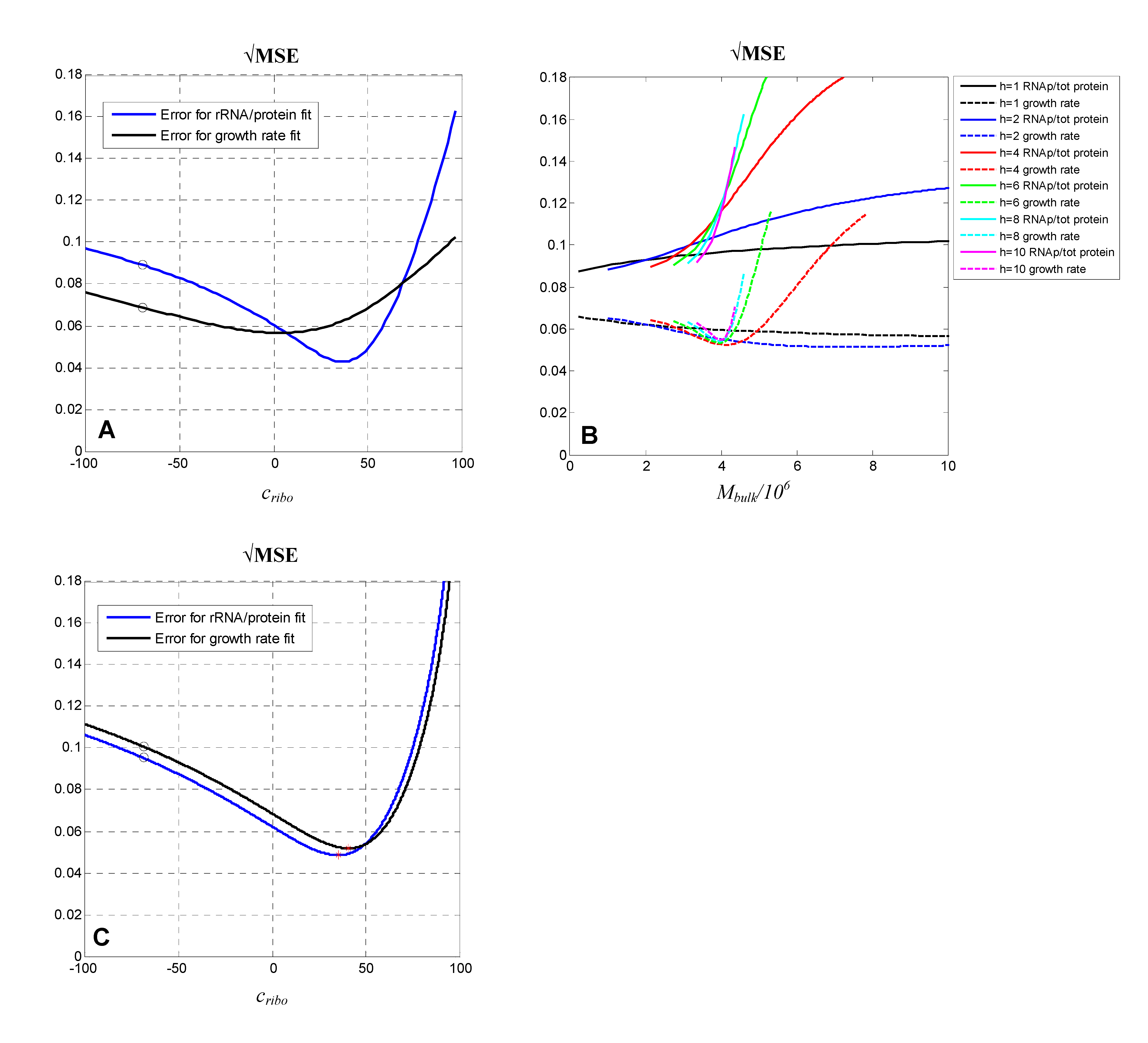

Supplement: Figure S1 — Mean square errors with respect to the Squires data. (A) Unconstrained CGGR MSE. Square root of the mean square error (MSE) as a function of cribo in estimation of the growth rate and the rRNA to total protein ratio measured by Asai et al. [19]. This graph was computed as follows: for a given n0, optimal Lm,bulk and cribo that minimize the square error between an estimated WT cell state and the observed WT cell state were obtained (see S1.1.1 in Text S1). Next, for those optimal Lm,bulk and cribo values, the growth rate curve and the rRNA/total protein curve were calculated for the various rrn inactivation strains (c.f. S1.3 in Text S1) and the MSEs were calculated between these two curves and the data points, yielding two errors for a given n0 (or equivalently cribo). Next, n0 is increased and the process is repeated. The minimum MSE for the rRNA to total protein ratio (which displayed more sensitivity to cribo than the growth rate) was obtained for cribo = 37.6 (n0 = 2.8·106 molec/WT cell). Circles mark the cost for which Φ would be fixed in an unconstrained CGGR model (i.e. when ci = −vi/vbulk, which is equivalent to the constrained CGGR model with h = 0). (B) Constrained CGGR MSE. Square root of the MSE in estimation of the growth rate and the rRNA to total protein ratio as a function of Mbulk and the Hill coefficient h, for a model where Φ is assumed to be fixed, and . This graph was computed as follows: for a given and h, optimal Lm,bulk and Mbulk that minimize the square error between the estimated WT cell state and the observed WT cell state were obtained. Note that this square error included the error between the estimated WT cp and the observed WT value of cp at 2 doub/h (20 aa/sec). The error in prediction of the WT cell state was on the order of a few percent (data not shown). Next, for those optimal Lm,bulk and Mbulk values, the growth rate curve and the rRNA/total protein curve were calculated for the various rrn inactivation strains and the MSE was ca [file pcbi.1000038.s009.tif]

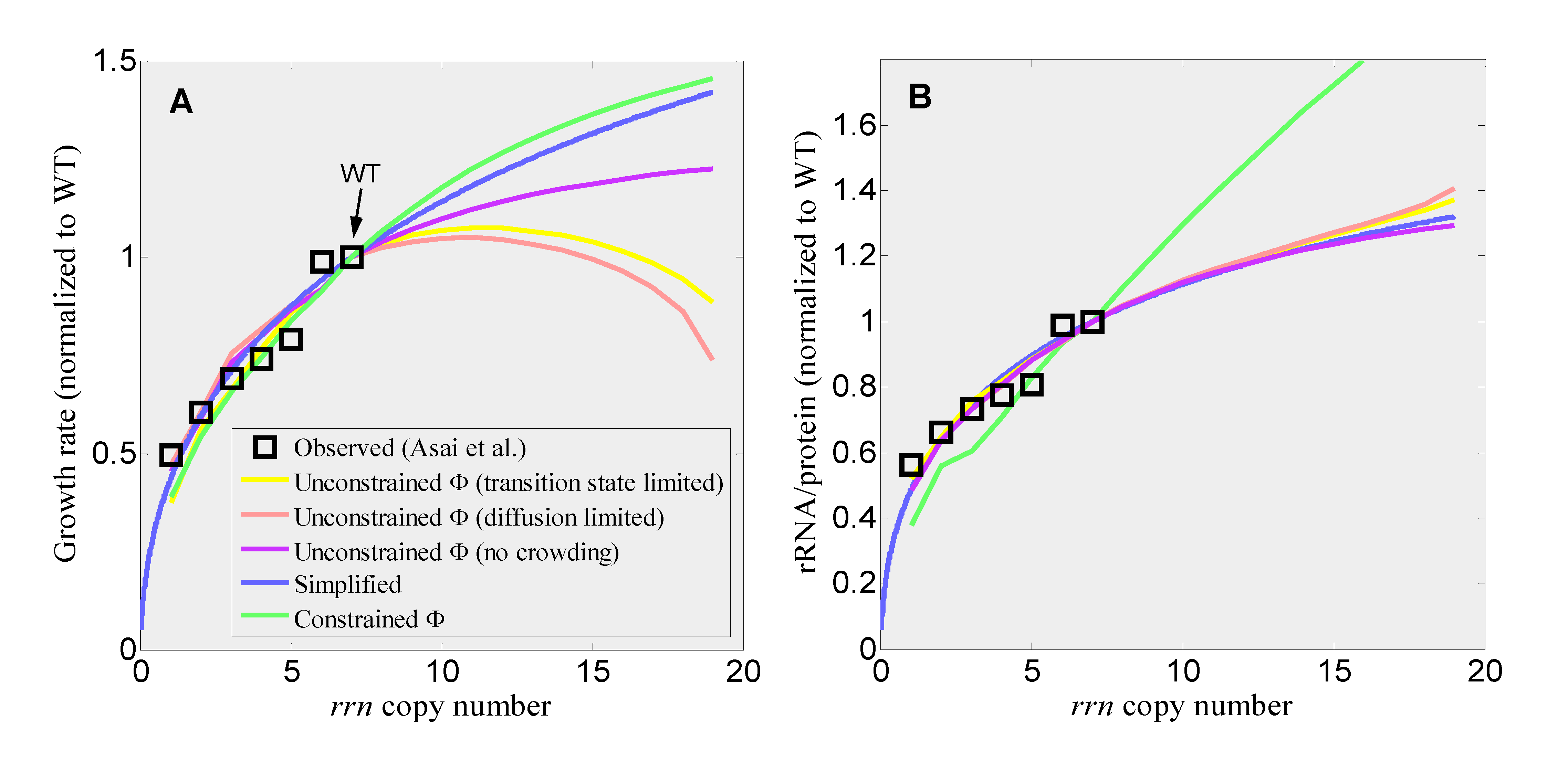

Supplement: Figure S2 — Fit for the constrained CGGR model with Hill coefficient h = 1. Comparison of the constrained CGGR model with Hill coefficient h = 1 to (A) growth rate measurements and (B) rRNA to total protein ratio measurements of Asai et al [19]. Mbulk was chosen such that the product of growth rate error and rRNA to total protein error was minimal, yielding Mbulk = 5.7·106 molec/WT cell (). For MSE see Figure S1B. Note that for h = 1, growth rate diverges with copy number. rRNA chain elongation rate, crrn, was assumed to be constant in this simulation. (0.27 MB TIF) [file pcbi.1000038.s010.tif]

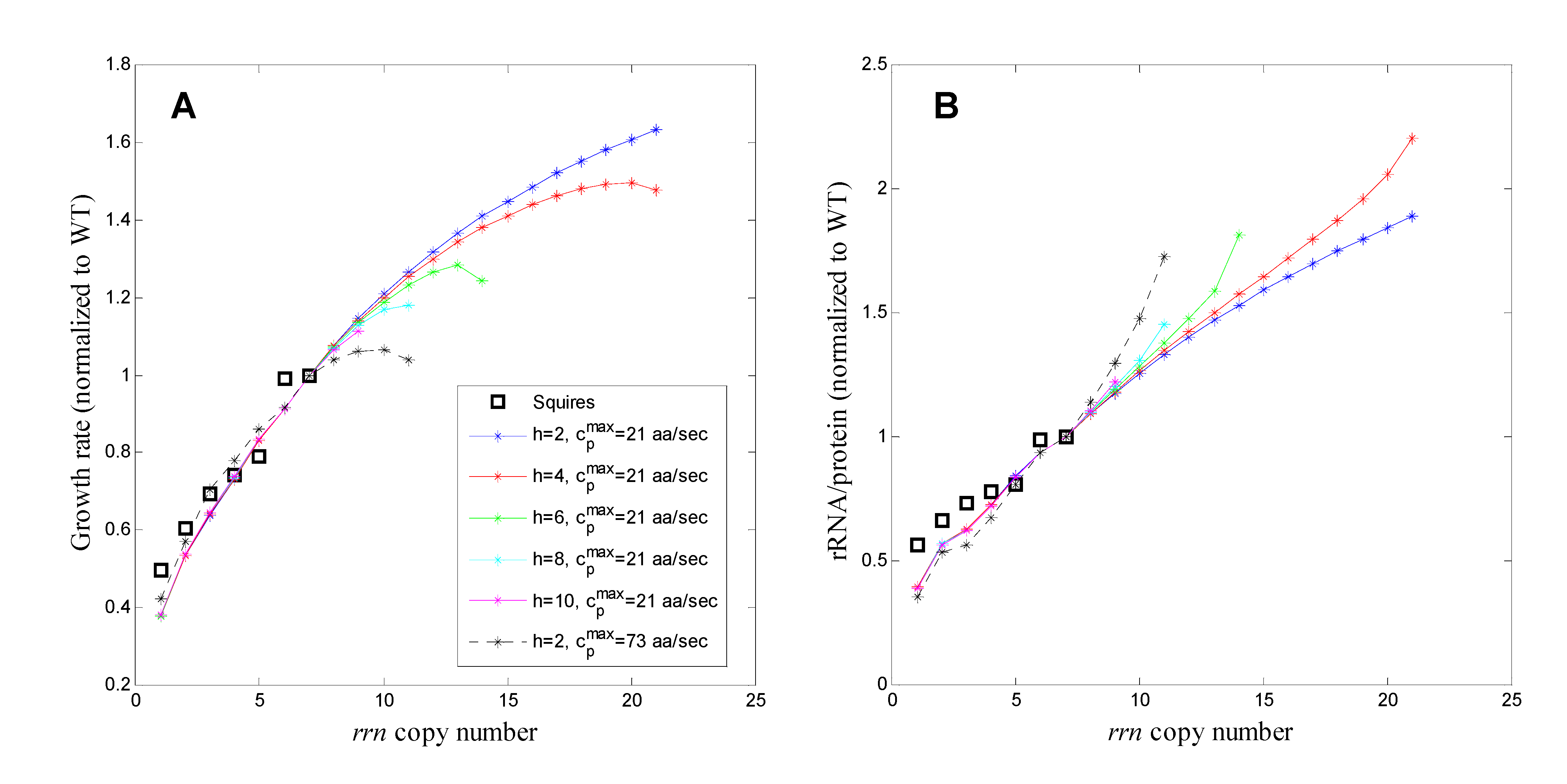

Supplement: Figure S3 — Fit for the constrained CGGR model with higher Hill coefficients. Comparison of the constrained CGGR model with Hill coefficients of 2, 4, 6, 8, and 10 to (A) growth rate measurements and (B) rRNA to total protein ratio measurements of Asai et al [19]. We show the h = 2 case for both (Mbulk = 7.4·106 molec/WT cell; as in Figure 2) and . For all other cases, was set to 21 aa/sec and corresponds to the minimum possible value for Mbulk., a value that according to Figure S1B minimizes the MSE for the rRNA to total protein ratio. This figure demonstrates that all solutions with diverge in growth rate for rrn copy numbers greater that 7. Higher Hill coefficients (>10) appear to be numerically unstable or insolvable for high copy numbers. Legend to both figures is given in (A). (0.22 MB TIF) [file pcbi.1000038.s011.tif]

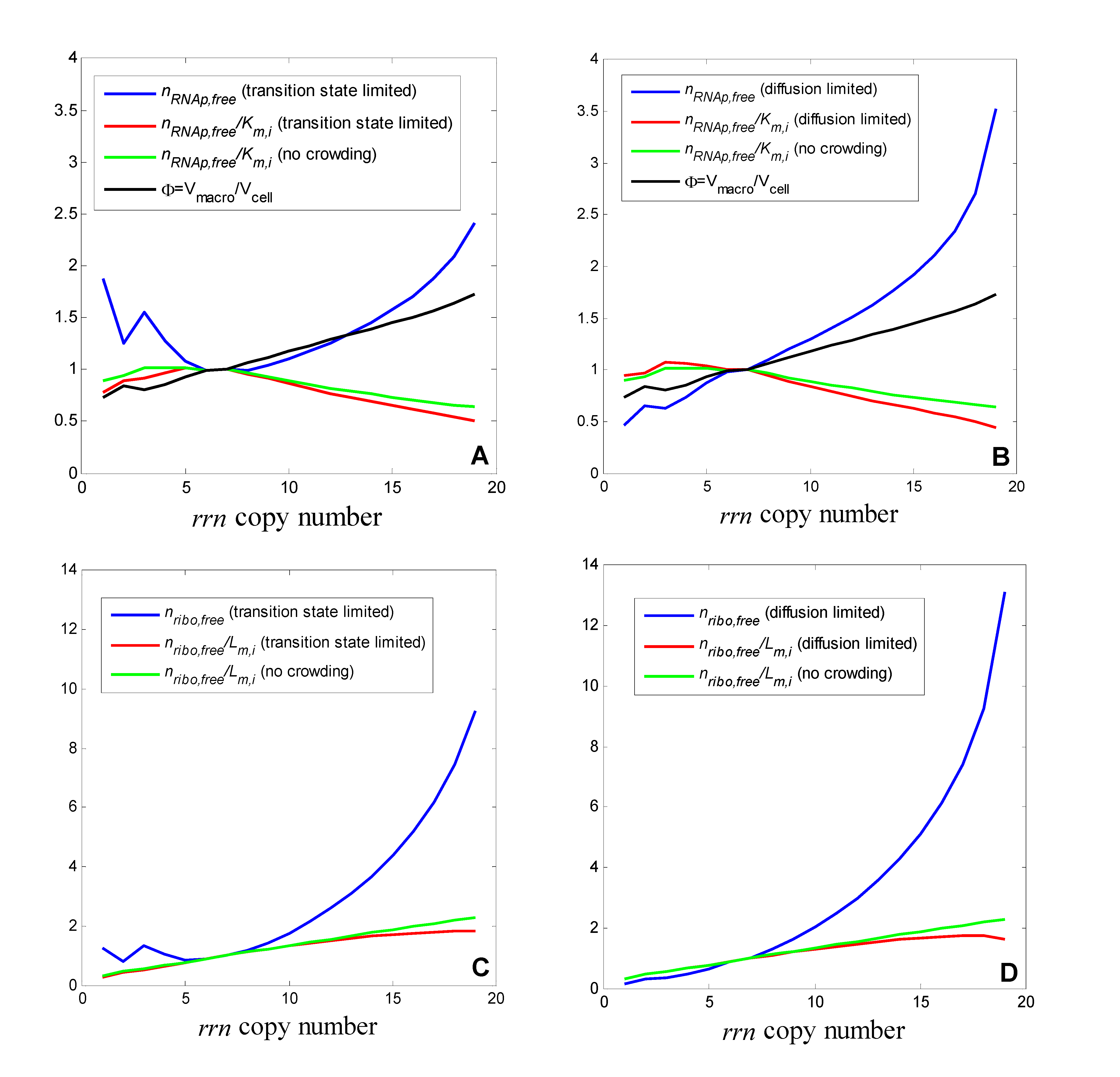

Supplement: Figure S4 — Free RNAp and free ribosomes with respect to corresponding binding affinities for various crowding scenarios. (A) Model prediction for nRNAp,free, nRNAp,free/Km,i and Φ for the transition state limited and no crowding scenarios as a function of the rrn operon copy number. In the no crowding scenario the plots for nRNAp,free and nRNAp,free/Km,i coincide. (B) Same as (A) but for the diffusion limited scenario. (C) Model prediction for nribo,free and nribo,free/Lm,i for the transition state limited and no crowding scenarios as a function of the rrn operon copy number. (D) Same as (C) but for the diffusion limited scenario. All curves are normalized to WT values at copy number 7. Note that in the diffusion limited scenario, when rRNA operons are inactivated, free RNAp concentration actually decreases. The reasons for this are that first, although the rRNA operons are inactivated, they continue to be partly transcribed (c.f. S1.3 in Text S1). Second, as rRNA operons are inactivated, growth rate is reduced (Figure 2A), which tends to slightly increase gene concentrations via Eq. 3 (c.f. Figure S7B). Finally, there is the contribution of increased transcription initiation. When rRNA operons are increased beyond seven copies per chromosome, free RNAp concentration increases mainly because transcription initiation is reduced due to diminished binding affinities. See main text and S1.6 in Text S1 for further explanations. (0.39 MB TIF) [file pcbi.1000038.s012.tif]

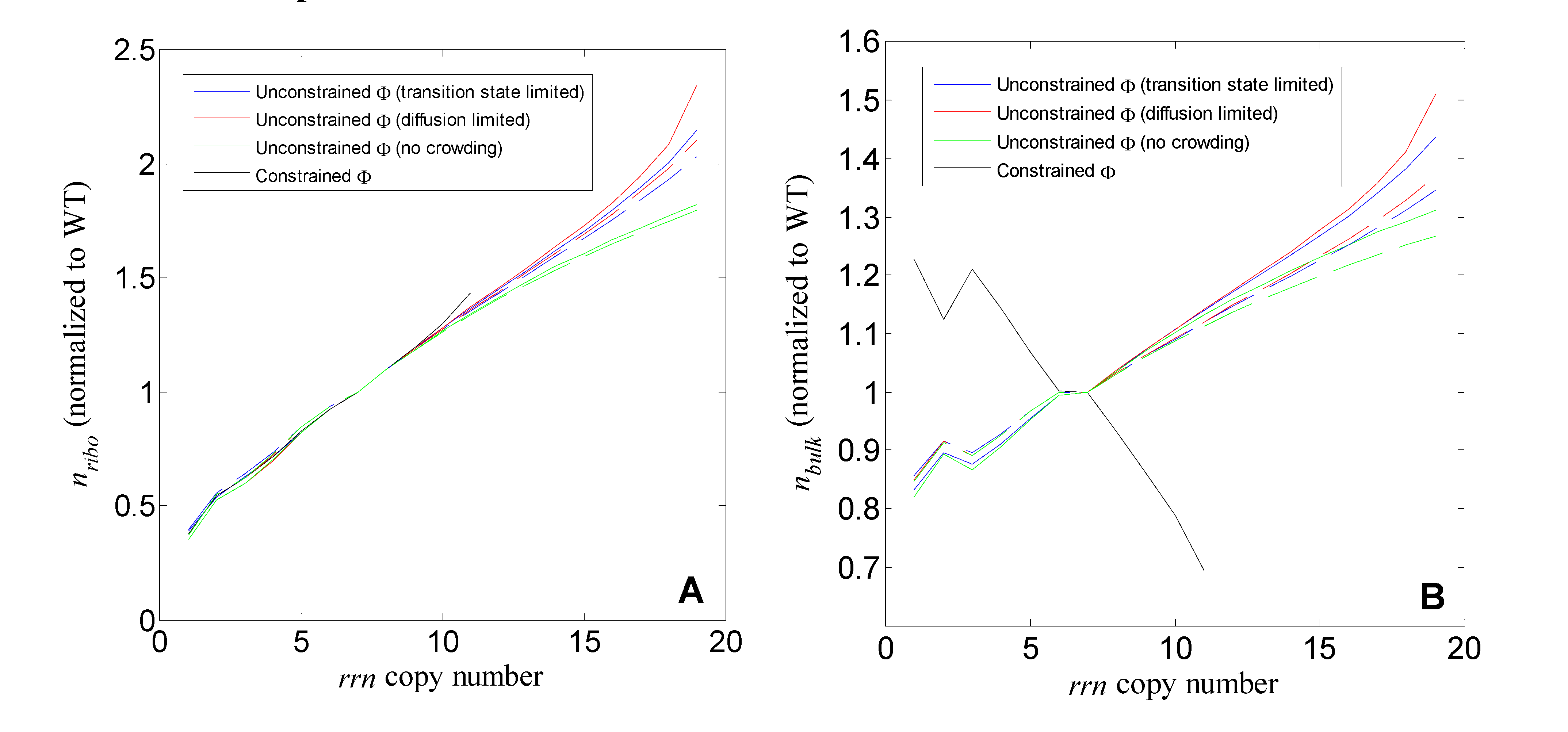

Supplement: Figure S5 — Predictions for bulk protein and ribosome concentrations as a function of the rrn operon copy number. (A) Total concentration of ribosomes (ribosomes per unit volume) in the constrained and unconstrained CGGR models as a function of the rrn operon copy number. (B) Concentration of bulk protein (proteins per unit volume) in the constrained and unconstrained CGGR models as a function of the rrn operon copy number. Solid lines are for fixed rrn chain elongation rate, crrn = const, and dashed lines are for crrn≠const, as described in the main text. All curves are normalized to WT cell state values (at copy number = 7). (0.22 MB TIF) [file pcbi.1000038.s013.tif]

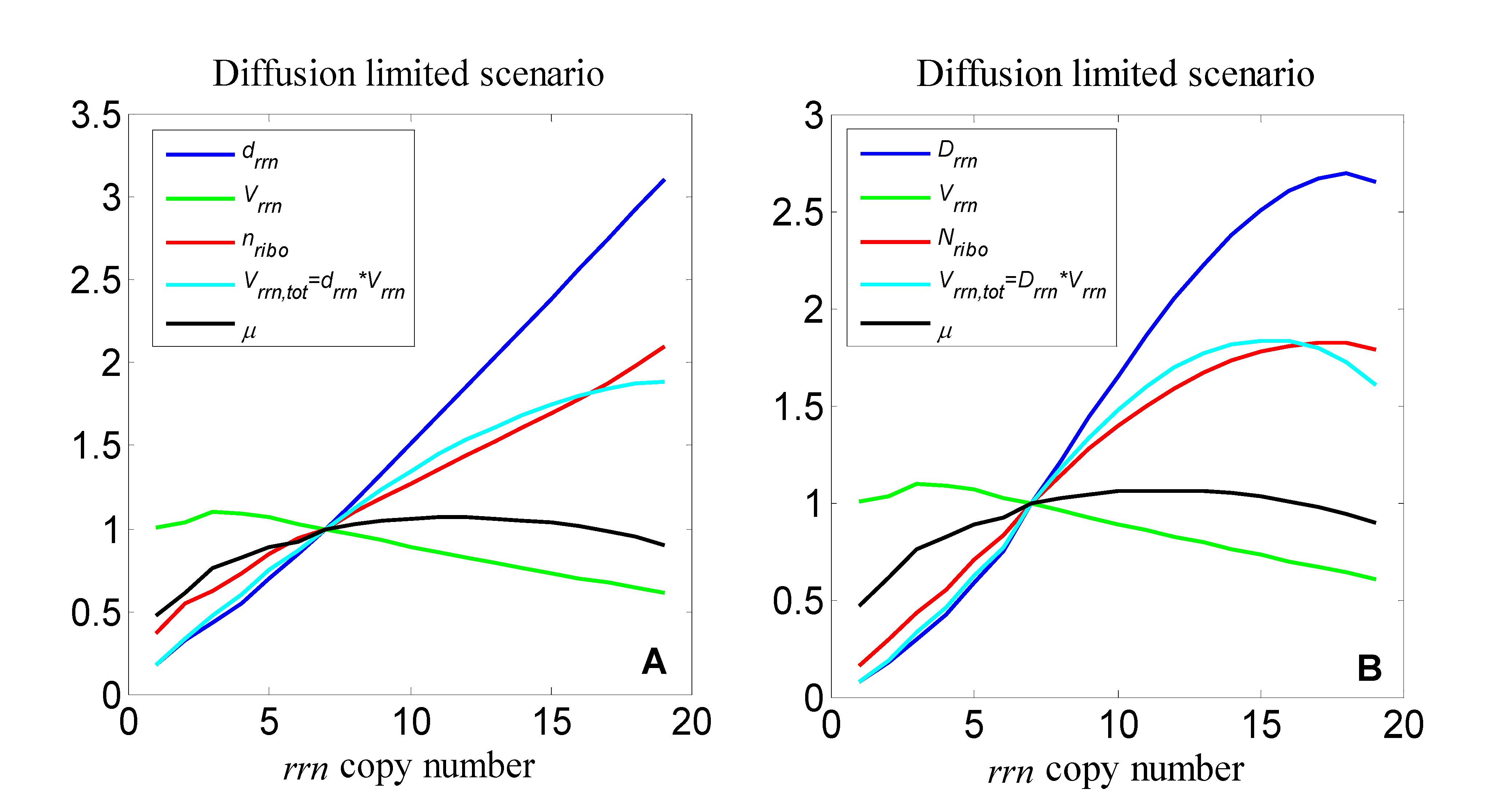

Supplement: Figure S6 — Breakdown of the ribosome synthesis equation to components for the diffusion limited scenario. (A) Variables in units of concentration. drrn - rrn gene concentration (total rRNA operon copy number per unit volume); Vrrn - rrn initiation rate per operon (init/min/operon); nribo - ribosome concentration (ribosomes per unit volume), and μ - growth rate. These parameters are tied by Eq. 2iii: α = drrn·Vrrn/nribo. (B) Variables in units of molec/cell. Drrn - rrn gene dosage (total rRNA operon copy number per cell); Nribo - number of ribosomes per cell. These parameters are tied by Eq. 2iii: α = Drrn·Vrrn/Nribo. This simulation is for the diffusion limited scenario assuming that the rRNA chain elongation rate, crrn, is variable, as described in the main text. All curves are normalized to WT cell state values (at copy number = 7). (0.22 MB TIF) [file pcbi.1000038.s014.tif]

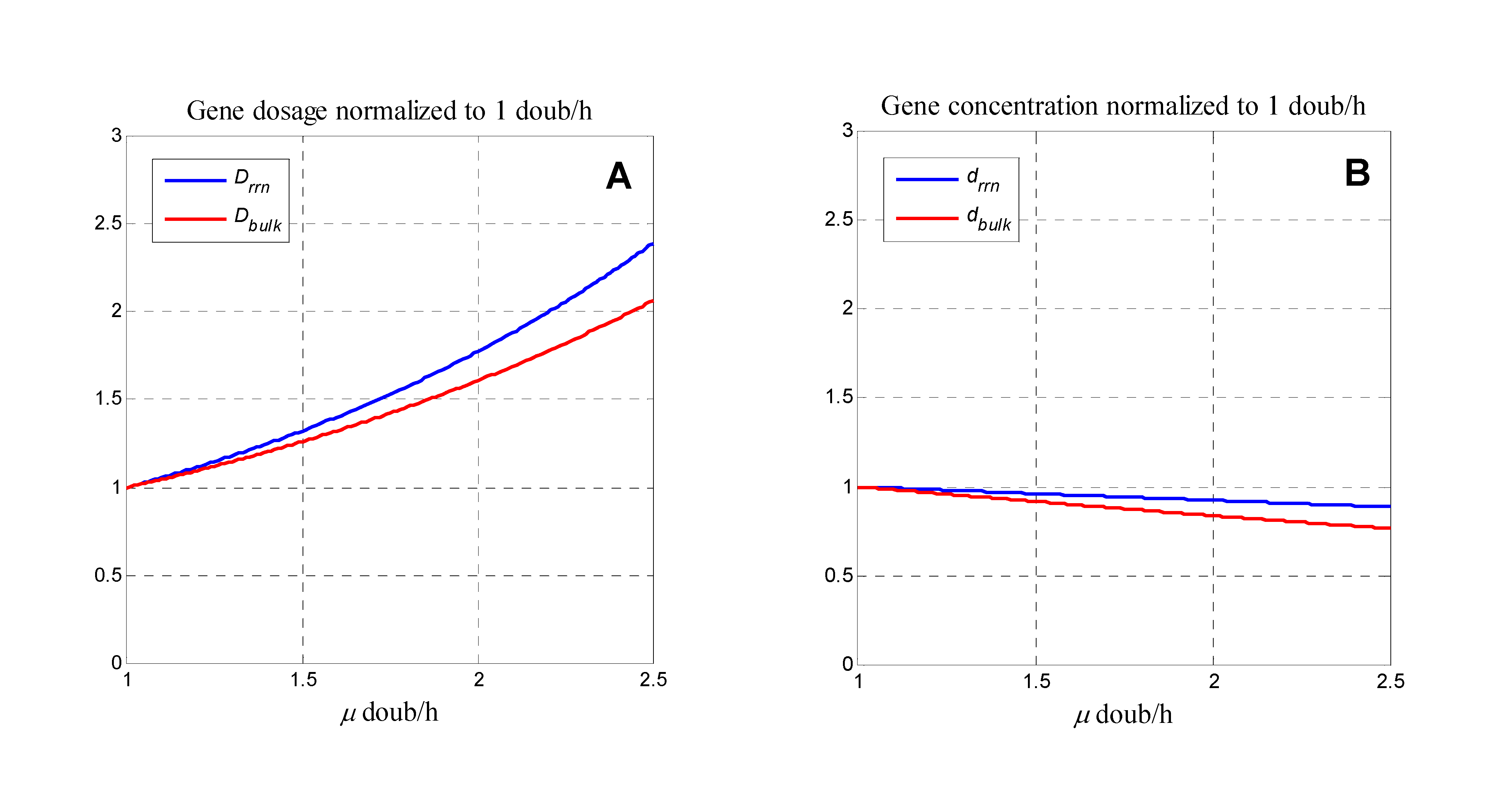

Supplement: Figure S7 — Gene dosage and gene concentration as a function of growth rate. (A) Gene dosage and (B) gene concentration for the rrn gene class and bulk gene class. C and D periods were interpolated based on data from table 2 of [17] as a second order polynomial in μ −1. For this simulation we assumed that 66 evenly distributed bulk genes are expressed (c.f. map locations in Table S1). The initiation volume, Vini, was assumed to be fixed [41],[43],[100]. See also main text and S2.2 in Text S1 for further explanations. (0.48 MB TIF) [file pcbi.1000038.s015.tif]

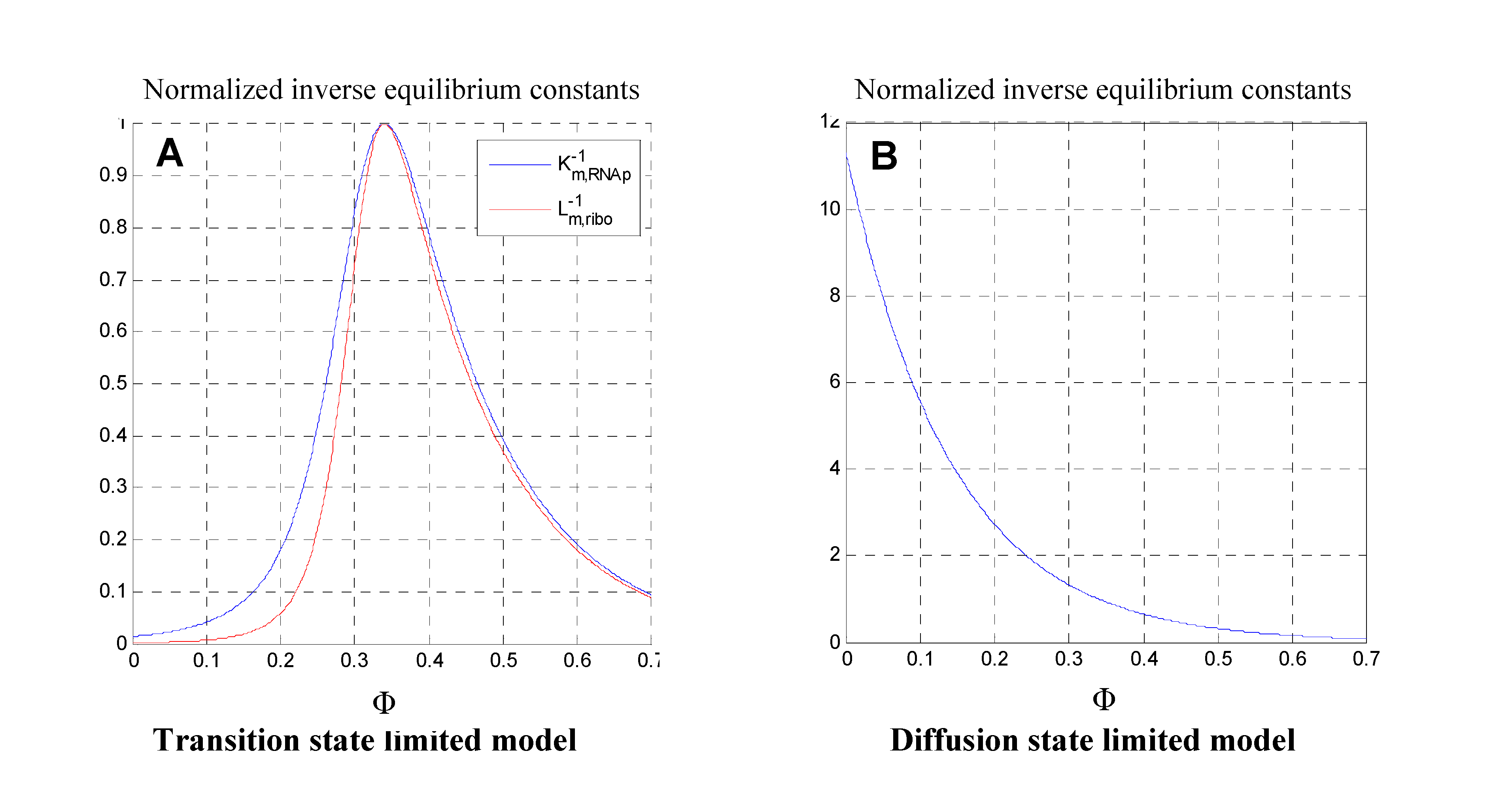

Supplement: Figure S8 — Dependence of binding affinities on the volume fraction Φ for the various crowding scenarios. (A) Normalized inverse equilibrium constants, Km −1 and Lm −1 (in units of 1/M), for the RNAp holoenzyme (radius 5.57 nm) and the 30S ribosome subunit (radius 6.92 nm), respectively, in the transition state limited model. The water molecule radius was taken to be 0.138 nm [101] and the radius of the background crowding agent was taken to be 3.06 nm [46]. (B) Normalized Km −1 and Lm −1 for the diffusion limited model (curves overlap). All curves were normalized to values at the WT volume fraction of Φ = 0.34. See S2.4 in Text S1 for more details. (0.46 MB TIF) [file pcbi.1000038.s016.tif]
